# Supplementary material for: Proteomic analysis reveals USP7 as a novel regulator of palmitic acid-induced hepatocellular carcinoma cell death
Source: Cell Death Dis. 2022 Jun 22;13(6):563. doi: 10.1038/s41419-022-05003-4 (PMC9217975; doi:10.1038/s41419-022-05003-4)

**Proteomic analysis revealed USP7 as a novel regulator of palmitic acid-induced hepatocellular carcinoma cell death**

Sandhini Saha^1^, Rohit Verma^2^, Chandan Kumar^3^, Bhoj Kumar^1^, Amit Kumar Dey^1^,

#### Milan Surjit^2^, Sivaram V S Mylavarapu^3^ and Tushar Kanti Maiti^1*^

^1^Functional Proteomics Laboratory, Regional Centre for Biotechnology, NCR Biotech Science Cluster, Faridabad, 121001, India.

^2^Virology Laboratory, Translational Health Science and Technology Institute, NCR Biotech Science Cluster, Faridabad, Haryana, India

^3^Laboratory of Cellular Dynamics, Regional Centre for Biotechnology, NCR Biotech Science Cluster, Faridabad, 121001, India.

^*^To whom correspondence should be addressed. E-mail: [tkmaiti@rcb.res.in](mailto:tkmaiti@rcb.res.in)

**Running title:** USP7 mediates cellular death in lipotoxic conditions.

**Supplementary Figures legends:**

**Supplementary Fig. 1**: PA-induced cytotoxic effects in HepG2 cells.

**Supplementary Fig. 2:** Overview of differential proteomics.

**Supplementary Fig. 3:** Malfunction of metabolic activity evoked upon lipotoxicity.

**Supplementary Fig. 4:** PA-induced USP7/Mdm2/p53 axis destabilization.

**Supplementary Fig. 5:** Phosphoproteome analysis of HepG2 cells treated with PA.

**Supplementary Fig. 6:** PA potentiates to induce AIF-mediated caspase-independent cell death.

**Supplementary Table 1:** Lists of differentially expressed proteins.

**Supplementary Table 2:** Annotation of differentially regulated network dynamics connected cell cycle and death through ubiquitin-dependent protein catabolic process

**Supplementary Data 1:** Expression pattern of differentially expressed candidates within two biological replicates.

**Supplementary Data 2:** List of metabolic activity that enriched in our mass spectroscopy dataset.

**Supplementary Data 3:** Gene ontology analysis of differentially expressed proteins.

**Supplementary Data 4:** KEGG pathway enrichment analysis.

**Supplementary Data 5:** Reactome pathway enrichment analysis.

**Supplementary Data 6:** Overview of phosphoproteins analysis.

**Supplementary Data 7:** Kinase-Substrate enrichment with Phosphosites in different time points.

**Supplementary Data 8:** Top 10 Reactome pathway executed through phosphoproteome.

**Supplementary Data 9:** Overview of AIF interatomic proteome in PA-induced condition.

**Supplementary Movie 1:** Confocal fluorescence time-lapse movie in control vehicle (CV) treated HepG2 cells transiently expressing H2B-mCherry (red) within a chromosome, showing the duration of mitosis.

**Supplementary Movie 2:** Confocal fluorescence time-lapse movie upon 0.5mM PA treatment in HepG2 cells transiently expressing H2B-mCherry (red), showing the duration of mitosis. The chromosomes showed congression defects at metaphase and lagging chromosomes during metaphase to anaphase transition.

**Supplementary Movie 3:** Confocal fluorescence time-lapse movie represents 0.5mM PA-induced chromatin condensation and fragmentation (chromatinolysis) due to incapacitation of successful mitosis.

**Supplementary Movie 4:** Confocal fluorescence time-lapse movie showing 0.5mM PA-induced chromosomal mis-congression that ultimately bypasses the metaphase. Further, unequal chromosomal segregation during anaphase leads to chromatin fragmentation, condensation, and finally preparing the cell for death through mitotic catastrophe.

**Supplementary Figures legends:**

**Supplementary Fig. 1**: PA-induced cytotoxic effects in HepG2 cells. MTT test was performed with the HepG2 cells upon treatment of 0.25 mM, 0.5 mM, 0.75 mM, 1.0 mM, 2.0 mM and 3.0 mM concentration of PA at 6 h, 12 h, 18 h , 24 h, 36 h and 48 h of exposure (A). PA concentrations up to 0.75 mM did not show much toxic in the early time points. However, cells showed significant sensitivity upon exposure of 0.75 mM concentration at 18 h, and a drastic reduction in cell viability was observed at 24 h (pValue <0.0001). More than 80 % cells were viable at 18 h with 0.5 mM PA concentration and 50% of cells were viable at 24 h of PA exposure. PA toxicity is primed with lipid accumulation within the hepatocytes as lipid droplets accumulation in HepG2cells analyzed by oil red-O assay. Representative photomicrographs of HepG2 cells (×60). Control cells vs. cells treated with 0.5 mM PA at 18 h (B). We perceived a significant increase of red intensity with 0.5 mM PA treated condition compared to the control vehicle at 18 h. PA-induced death assessments were done by annexin V-FITC assay. The dot plot denotes the distribution of the cells (in percentage) among all four quadrants (C) and their quantifiable data is represented via a bar graph (D). We did not detect much change in cell death with BSA treatment throughout all time points. However, we found a reduction of healthy cell population upon PA exposure at 12 h time points onwards. In PA exposure, a significant population was shifted in the early and late quadrant at 24 h, 36 h, and 48 h. The representative statistical result was performed using the Two-way ANOVA test followed by Tukey's multiple comparisons test and data shown as means ± SEM from three independent experiments. *p<0.05, **p<0.01, ***p<0.001, ****p<0.0001 and ns=non-significant.

**Supplementary Fig. 2:** The probability distribution curve across the total expressed proteins and the cut-off range for DEPs were selected based on the standard distribution curve (A). Venny diagram showed common proteins within two biological replicates that are efficiently identified with 1% FDR (B). Bar plot representing the differentially expressed proteins between two biological replicates Criteria P-value<0.05, fold difference:>1.3 upregulated and <0.7 down-regulated (C). Over-represented GO analysis of differentially expressed proteins that enlisted in PA treated HepG2 cells were done by WEB-based GEne SeT AnaLysis Toolkit. Bar diagram denoted positive (red) and negative related (green) biological processes according to their normalized enriched score (NES). The Colour gradient of red and green bar represented the FDR value. (Darker the color ≤0.05 FDR) (D). Enriched cellular compartmentalization (GO-CC) (E) and molecular functions (GO-MF) (F) were described by bubble plot. Protein quality control associated cellular compartment was highlighted in blue color dot. According to the NES, positive related molecular functions were shown by red color dots and blue dots were denoted by negative one. In Gene Set Enrichment Analysis (GSEA) of no Redundant geneontology studies (GOBP and GOMF), significance level (FDR) was set at <0.5 and. A minimum 20 number of ID were chosen in a particular one category. Where Over Representation Analysis (ORA) of GOCC was executed in BH FDR (<0.05) method.

**Supplementary Fig. 3:** Malfunction of metabolic activity evoked upon lipotoxicity. Bar plot showing anomalous metabolic processes operated upon PA-induced condition based on STRING (v11.5) analysis. The data has represented based on the gene count, and dual-color denoted the FDR ratio (-Log10) (A). The representative three bar plot showed the translating gene lists into three major domains of biological processes such as regulation of cell death, cell cycle, and ubiquitin-dependent catabolic process networks in lipotoxic conditions (B-D). Screening of individual domain comprised a large sub-node BPs (Biological Process noRedundant) in FDR≤0.05 with a significant enrichment ratio.

**Supplementary Fig. 4:** PA-induced USP7/Mdm2/p53 axis destabilization. PA promotes USP7, p53, and Mdm2 destabilization and degradation through proteasomal machinery. HepG2 cells were co-incubated with PA (0.5 mM) and MG-132 with an increasing concentration ranging from 5 μM to 20 μM for 24 h. Immunoblot against USP7, Mdm2 and p53 were shown in the figure (Fig. A), where GAPDH was used as a loading control. Normalized densities of each condition were shown by bar graph (B-D). Ordinary one-way ANOVA followed by Tukey's multiple comparisons test was applied for statistical analysis. Error bar represents mean±SEM; n= 3; ns=no significant. The control vehicle maintains cell cycle integrity throughout the all-time points. HepG2 cells were harvested after exposure to the control vehicle for cell cycle analysis (F), and the percentage of cell distribution through all phases was presented in the bar graph(G). Cell cycle distribution mapping was done every 12 h intervals till 48h. Two-way ANOVA followed by Tukey's multiple comparisons test was applied for statistical analysis, but no significant changes were observed within the same phases of different time points. Error bar represents mean±SEM; n= 3.

**Supplementary Fig. 5:** Phosphoproteome analysis of HepG2 cells treated with PA. Schematic representation of temporal-phospho-proteomics analysis of HepG2 cells. Upon exposure to palmitic acid (0.5 mM) cells were harvested at 0, 6, 12, 18, and 24 h time points. The experimental workflow accompanied with each significant step like protein extraction, acetone precipitation, tryptic digestion, and phoshphopeptide enrichment by TiO2 resin beads to gain more phosphoproteins coverage, and Nano-LC-MS/MS followed by data analysis and phosphoprotein and kinases identification(A). Phosphoproteins along with phosphopeptides were represent by bar graph for all time points (B). Identified phosphoproteins in different time points were presented by venn diagram (C). True positive phospho signals were evaluated on the basis of significant normal distribution of the PEP score against all phoshpho-peptides and finally the cut-off value were implicated (>10 PEP score) (D). We retrieved the lists of modified phosphosites from phosphopeptides with the help of PhosphoSitePlus. A web-based tool, Kinase Enrichment Analysis 2 (KEA2) were used for the prediction of kinases activity using phosphosites of the respective phosphoproteins for all time points (0-24 h) (E-F). The X-axis were denoted the total number of hits and y-axis denoted the pValue (-log10) of enriched kinases.

**Supplementary Fig. 6:** PA potentiates to induce AIF mediated caspase independent cell death. Mitochondrial membrane potential (MMP) was lowered down upon 24h of PA exposure and measured by flow cytometry (A). The cells treated with CCEP (100 µM) were used as a positive control. Ordinary one-way ANOVA followed by Dunnett's multiple comparisons test was applied for statistical analysis. Palmitic acid induces AIF translocation to the nucleus. Subcellular fractionation of PA treated lysates were subjected for AIF, CypA localization within mitochondrial and nuclear fractions at 0, 12, 24, and 48 h time points. CoxIV and Histone3 were used as a positive loading control for mitochondria and nucleus, and β-actin was used as a negative loading control for cytoplasmic contamination (B). PA promoted AIFM1 accumulation in the nucleus. AIFM1 (green signal) distribution were visualized within cytosol (red, MITO TRACKER) and nucleus (blue, DAPI) in 0.5 mM PA and control vehicle treated HepG2 cells at 24 and 48 h time points (C). The Pearson coefficient were quantified based on co-localization of AIFM1 with DNA damage marker γH2A.X (Ser139) (pink) (D). The cellular distribution ratio of AIF (nucleus/ cytosol) were represented in the bar graph (E). Ordinary one-way ANOVA followed by Tukey's multiple comparisons test was applied for statistical analysis. Further, PA induced complete degradosome formation were confirmed by colocalization of CypA (green) along with DNA damage marker γH2A.X (Ser139) (pink). MitoTracker was used to stain mitochondria in red and co-staining with DAPI to stain nucleus (F). The overlay channel for all merged conditions were shown on the right panel with 10 µm scale bar. Quantifications were performed with co-localization Pearson’s value for CypA and pH2AX (Ser139) (G). A two-tailed unpaired t-test was applied for statistical analysis. All values were plotted against mean ± S.E. (error bars), n ≥ 3. *p<0.05, **p<0.01, ***p<0.001, ****p<0.0001, and ns=non-significant.

**Supplementary Figure 1:**


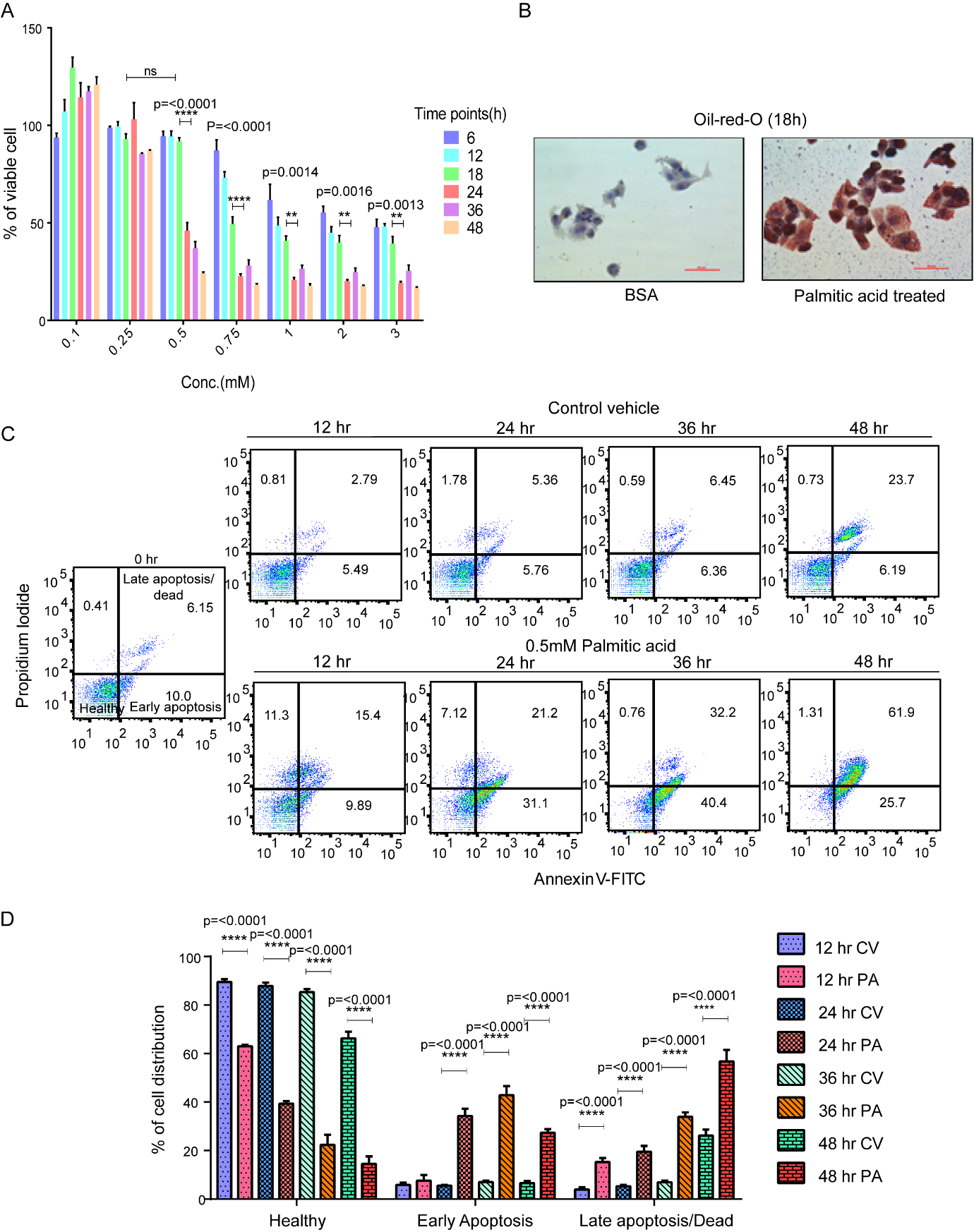


**Supplementary Figure 2:**


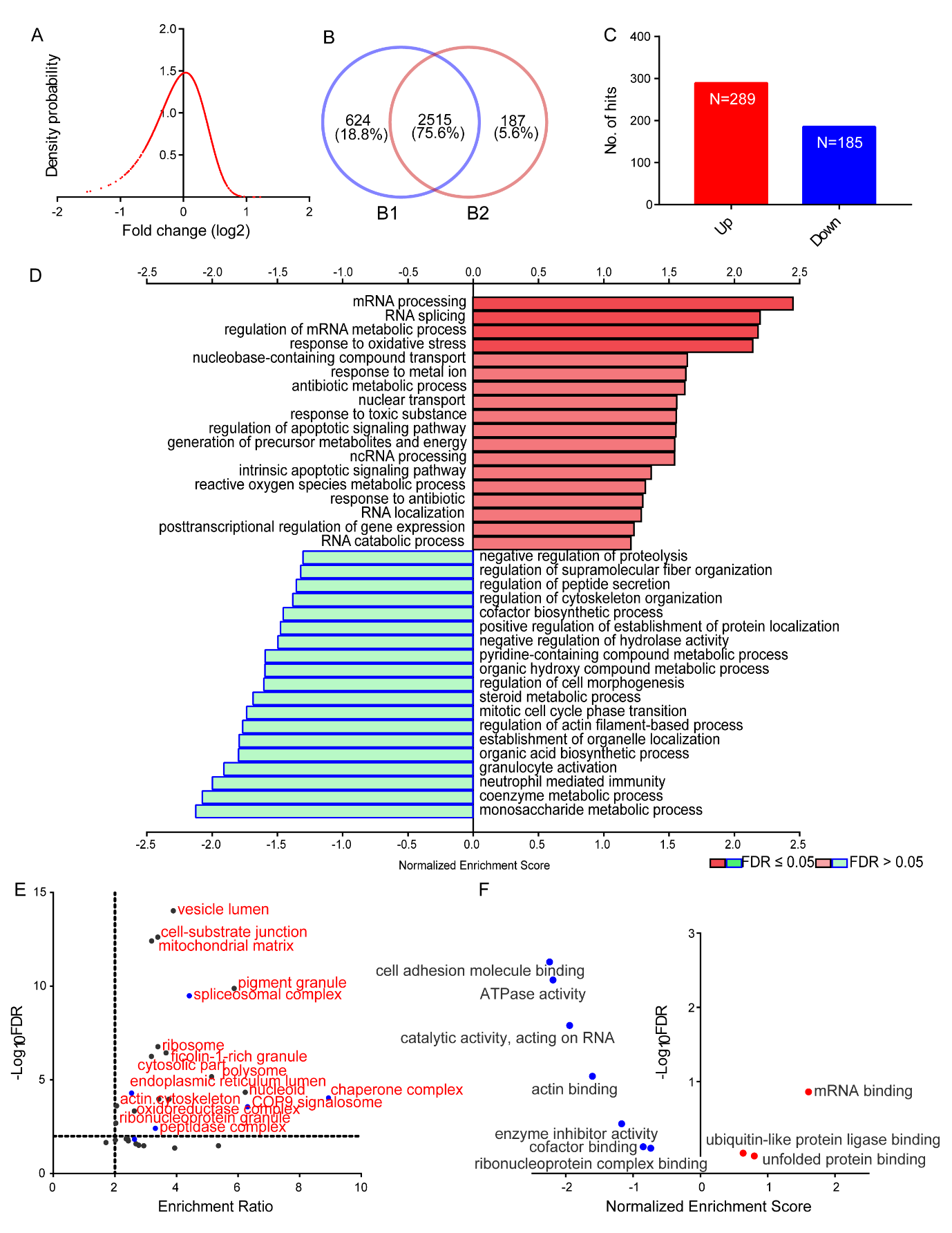


**Supplementary Figure 3:**


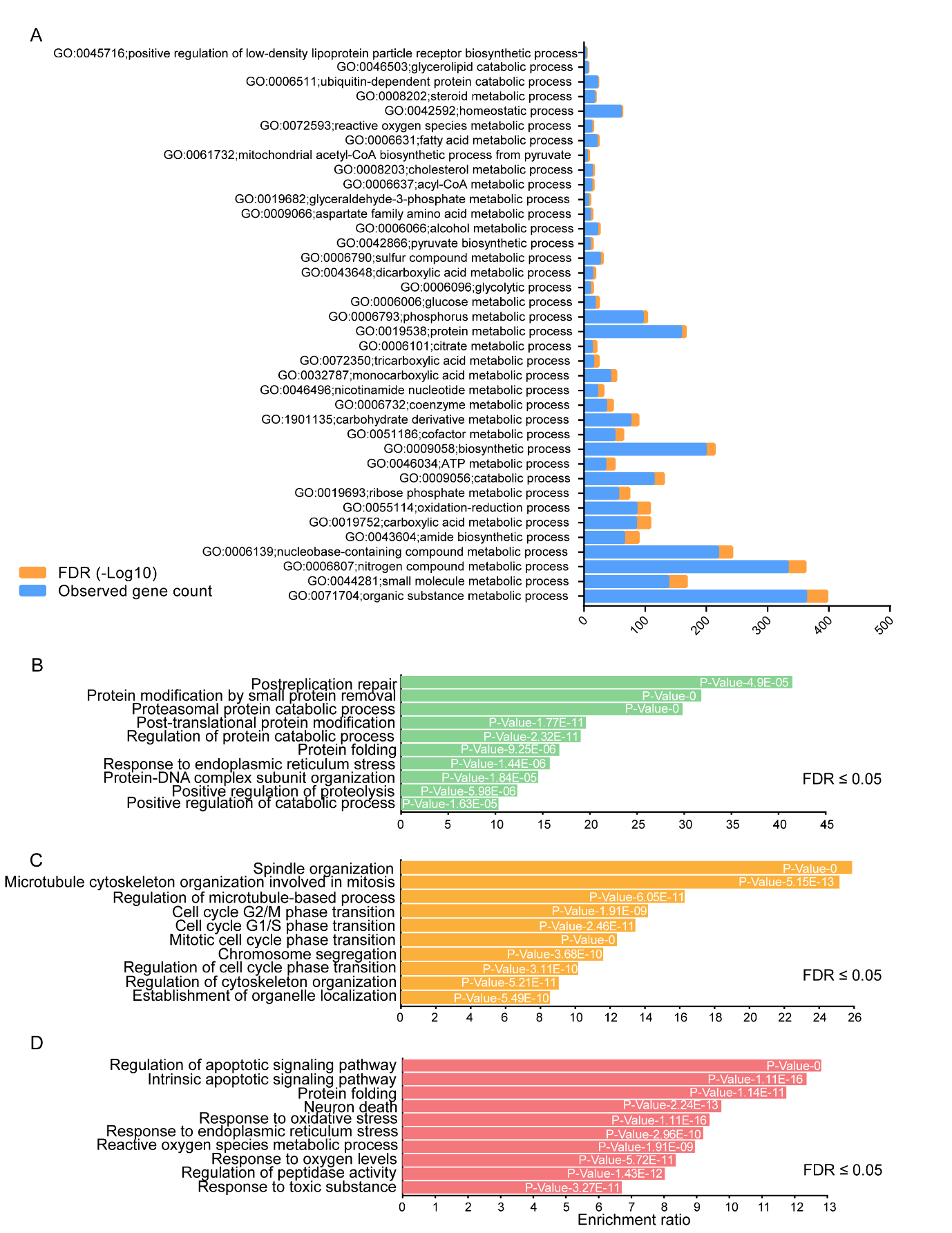


**Supplementary Figure 4:**


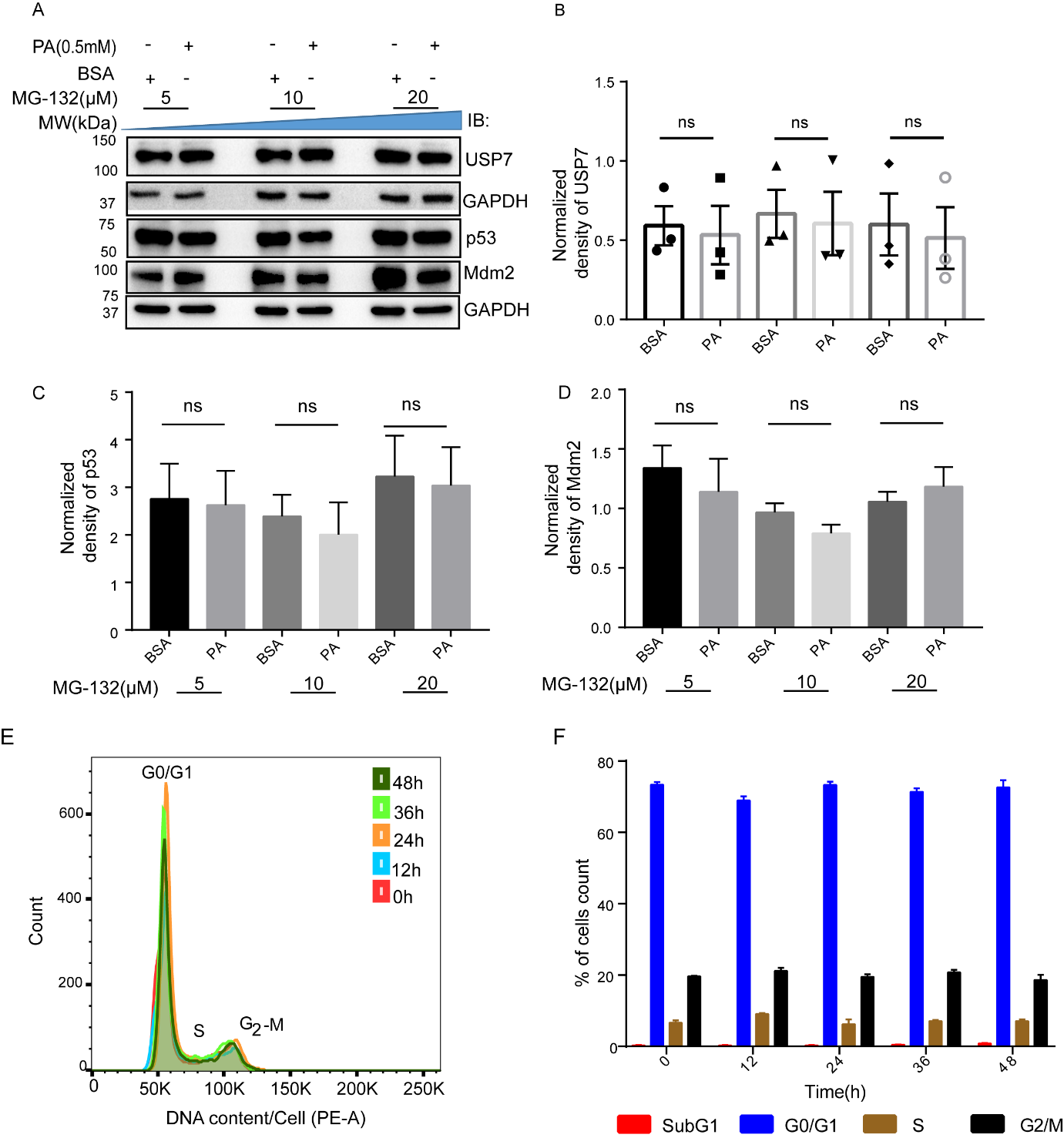


**Supplementary Figure 5:**


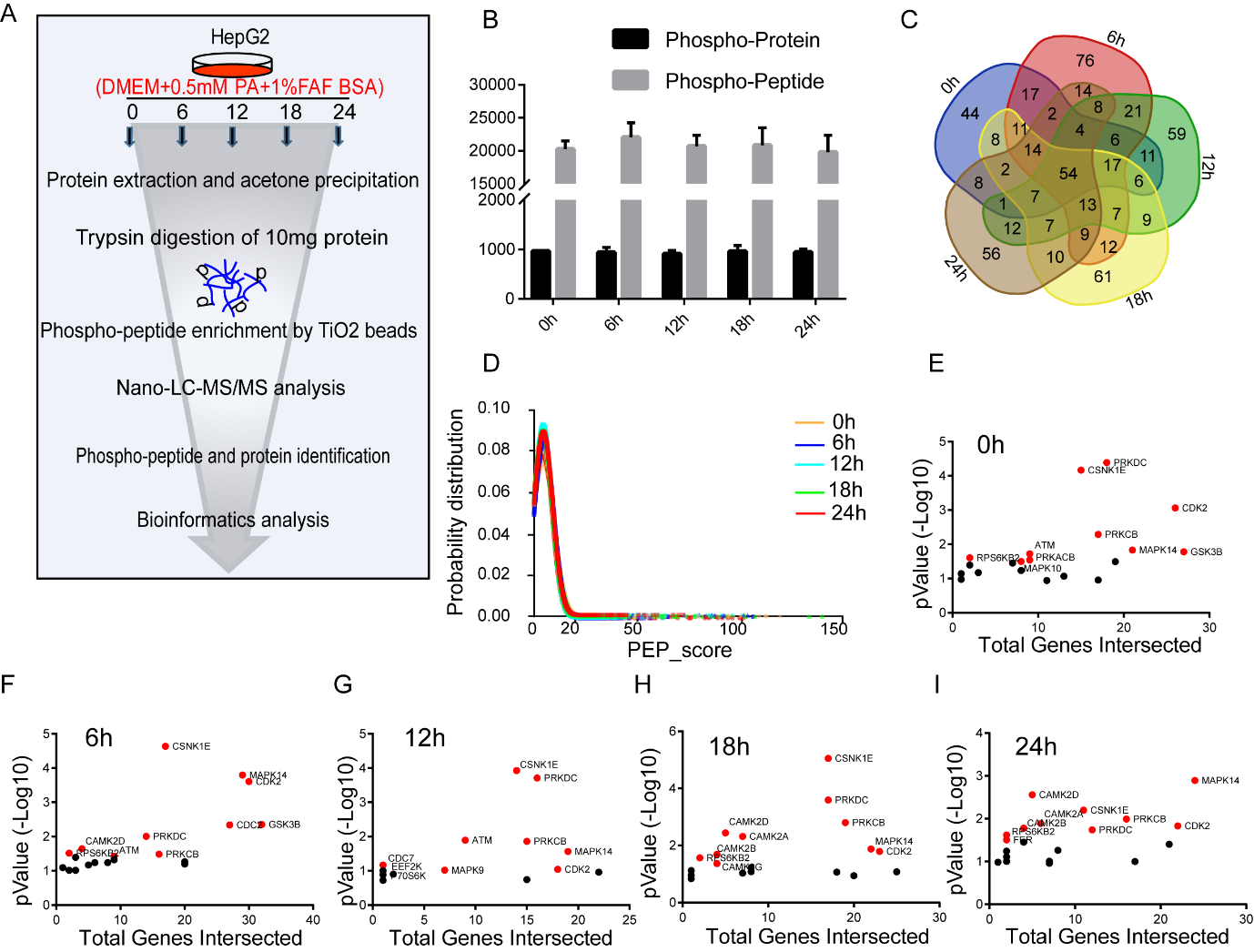


**Supplementary Figure 6:**


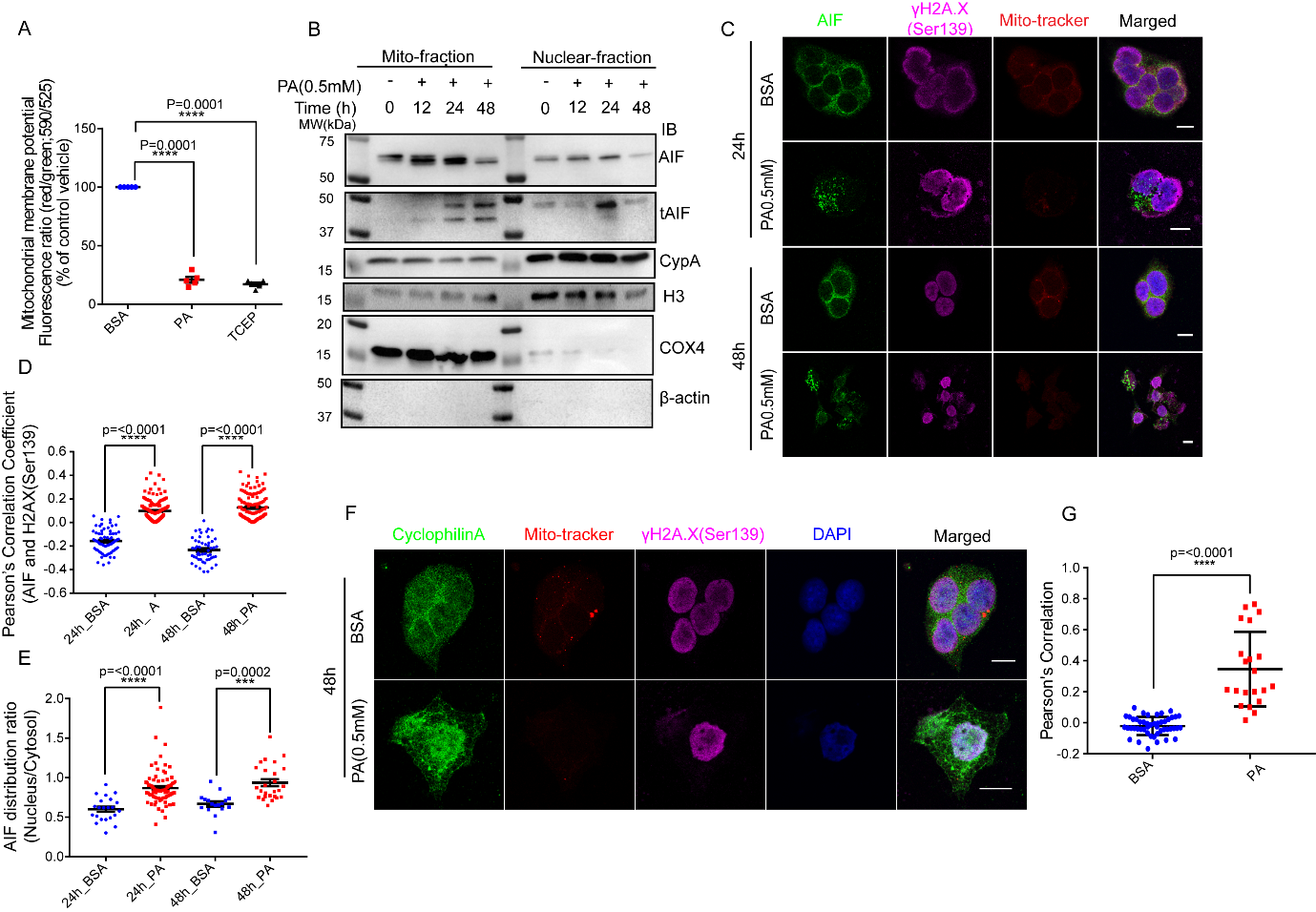

Supplement: Supplementary file 5 — Supplementary_CDD [file 41419_2022_5003_MOESM5_ESM.docx]
