# Supplementary material for: Proteomic analysis reveals USP7 as a novel regulator of palmitic acid-induced hepatocellular carcinoma cell death
Source: Cell Death Dis. 2022 Jun 22;13(6):563. doi: 10.1038/s41419-022-05003-4 (PMC9217975; doi:10.1038/s41419-022-05003-4)
Supplement: Supplementary file 7 — Western_blot-raw image_Main Figure [file 41419_2022_5003_MOESM7_ESM.pdf]

Proteomic analysis revealed USP7 as a novel regulator of palmitic acid-induced hepatocellular carcinoma cell death

Figure: 2B

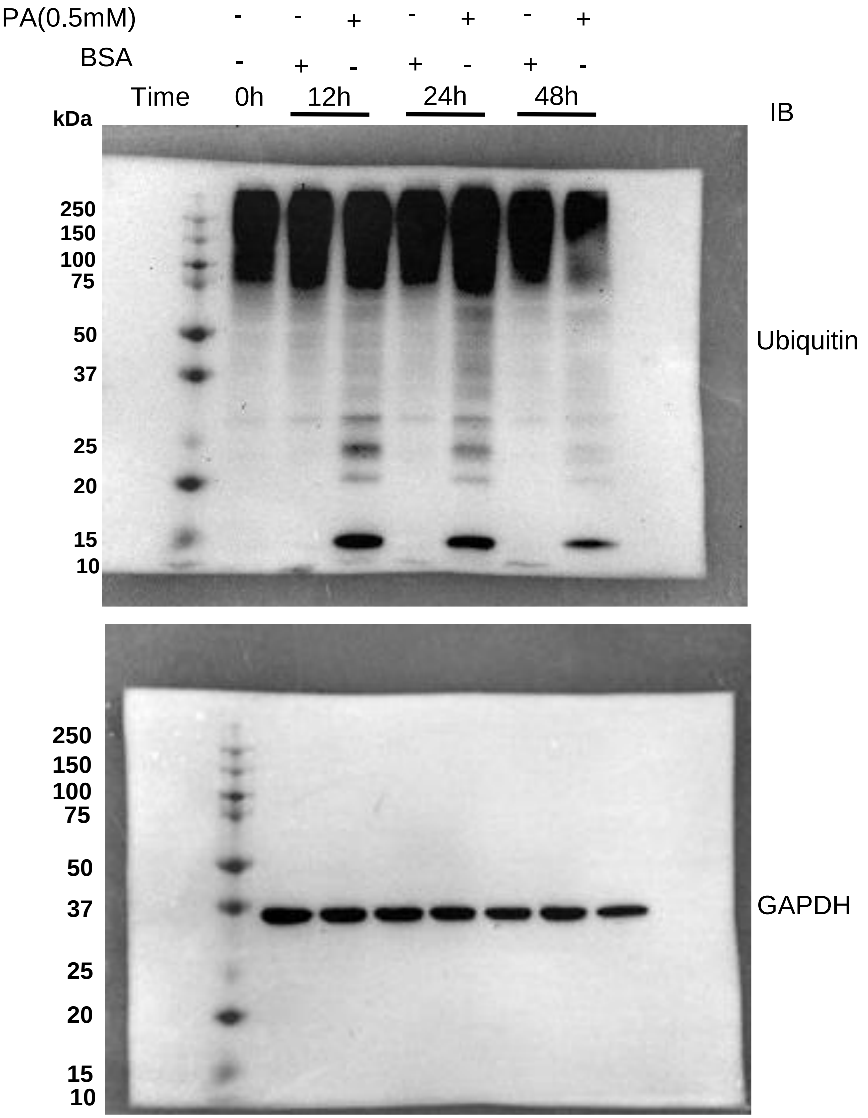

Figure: 2C

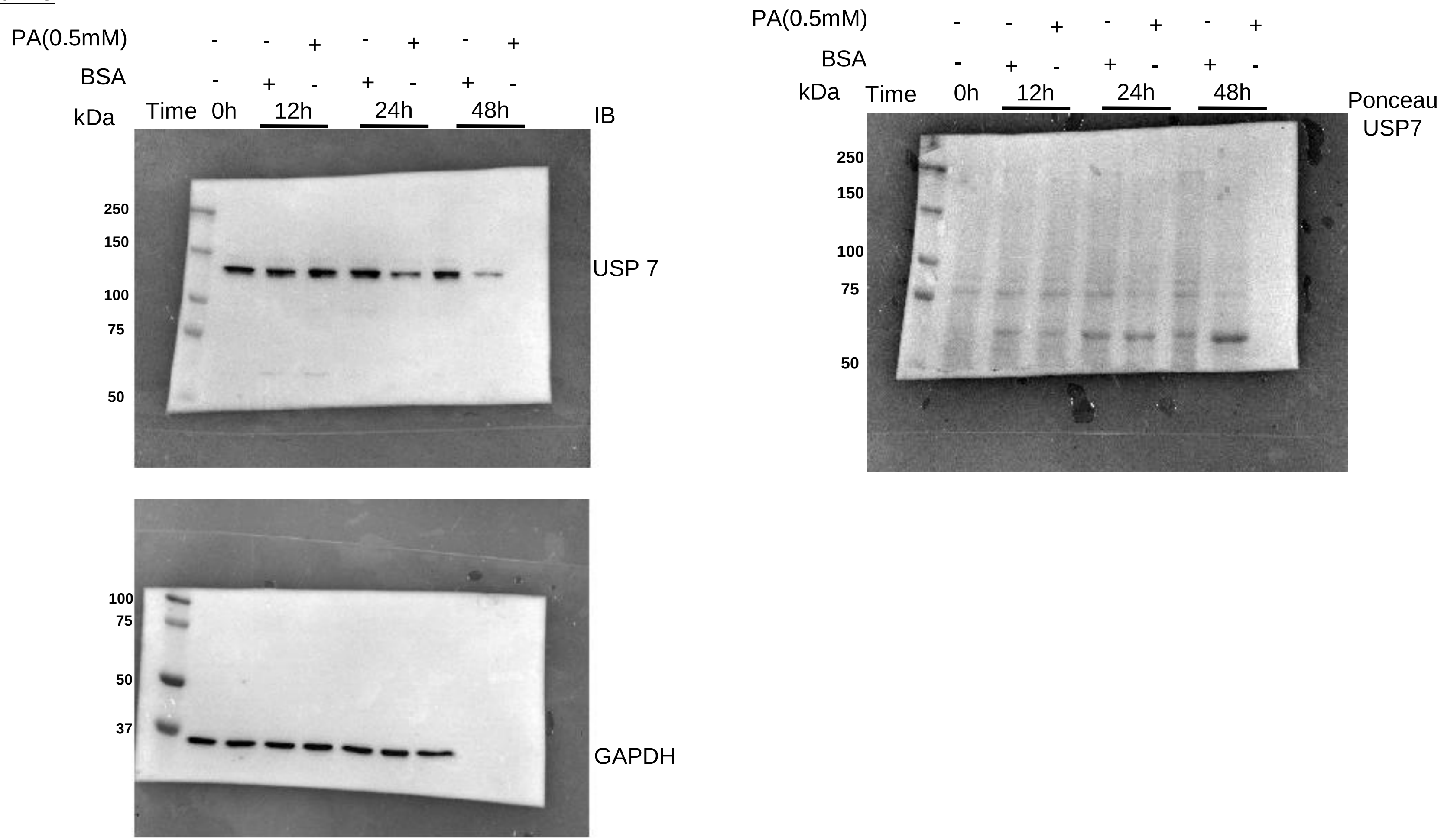
PA(0.5mM) - - + - + - +
BSA - + - + - + -
kDa Time 0h 12h 24h 48h
IB
250
150
100
75
50
USP 7
100
75
50
37
GAPDH
PA(0.5mM) - - + - + - +
BSA - + - + - + -
kDa Time 0h 12h 24h 48h
Ponceau
USP7
250
150
100
75
50

Figure: 3A

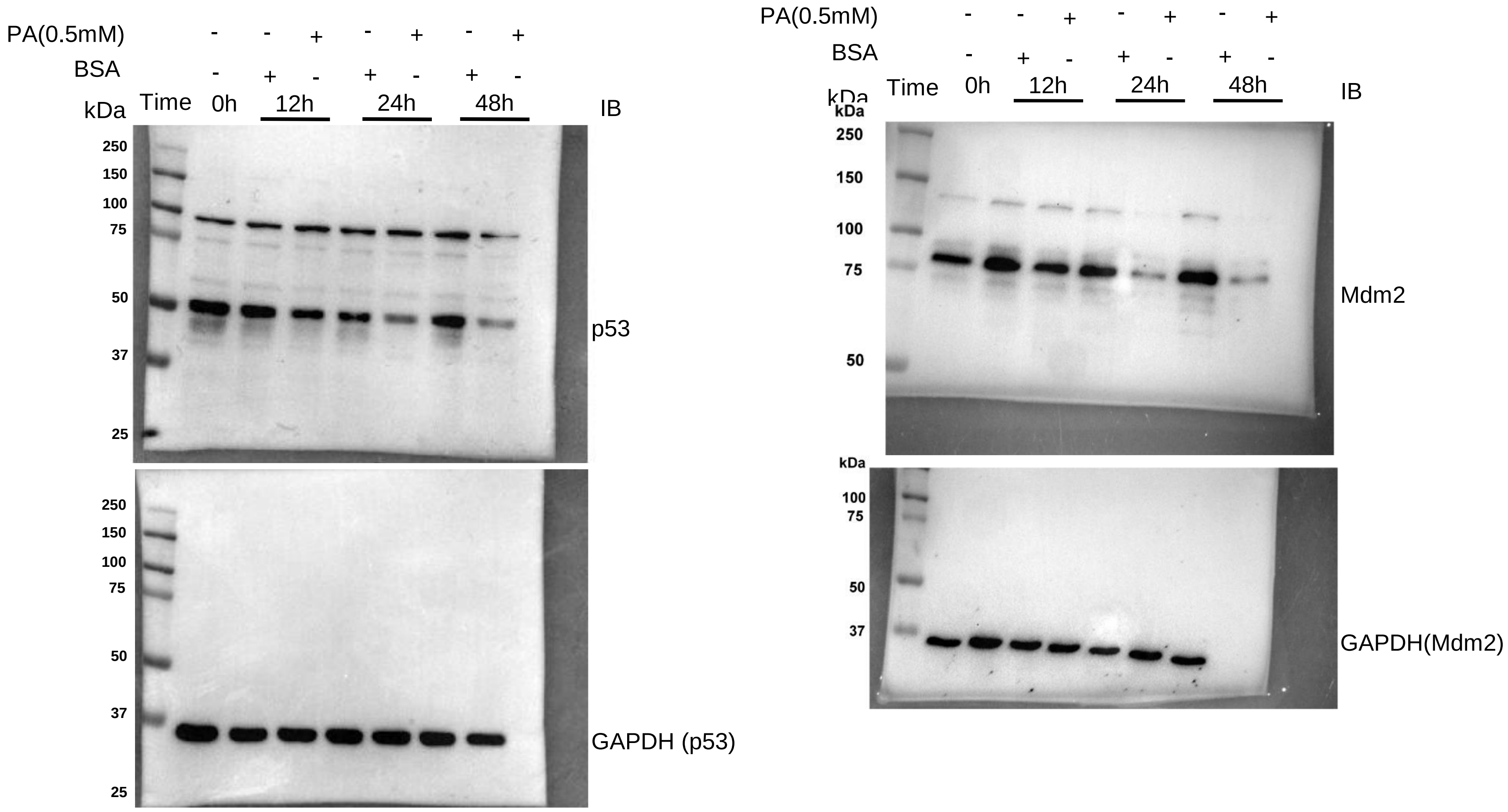
PA(0.5mM)
BSA
kDa
Time
0h
12h
24h
48h
IB
250
150
100
75
50
37
25
p53
GAPDH (p53)
Mdm2
GAPDH(Mdm2)

Figure: 3D

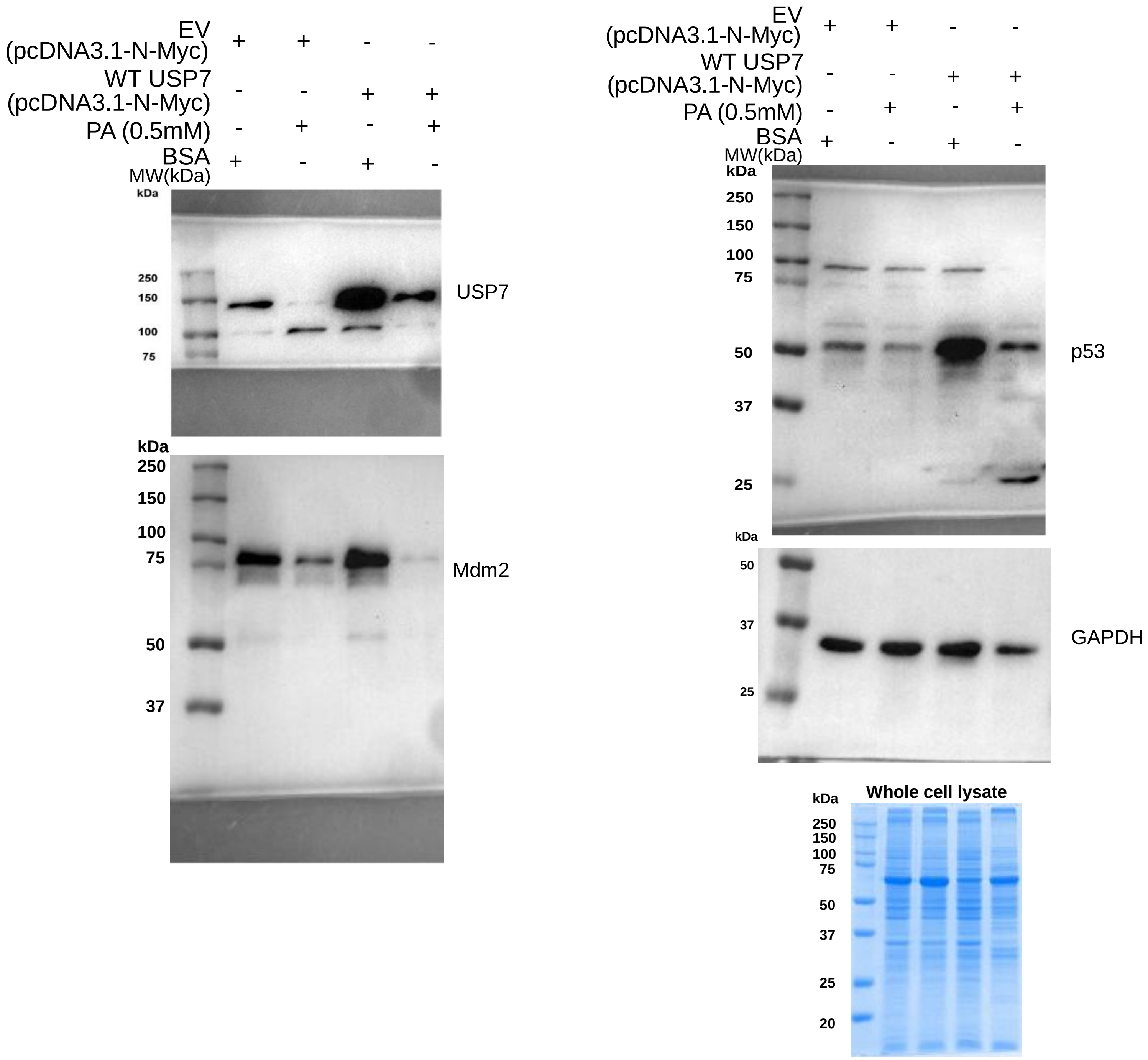
EV (pcDNA3.1-N-Myc) + + - -
WT USP7 (pcDNA3.1-N-Myc) - - + +
PA (0.5mM) - + - +
BSA + - + -
MW(kDa)
kDa
250
150
100
75
USP7
kDa
250
150
100
75
50
37
Mdm2
EV (pcDNA3.1-N-Myc) + + - -
WT USP7 (pcDNA3.1-N-Myc) - - + +
PA (0.5mM) - + - +
BSA + - + -
MW(kDa)
kDa
250
150
100
75
50
37
25
p53
kDa
50
37
25
GAPDH
Whole cell lysate
kDa
250
150
100
75
50
37
25
20

Figure: 3E

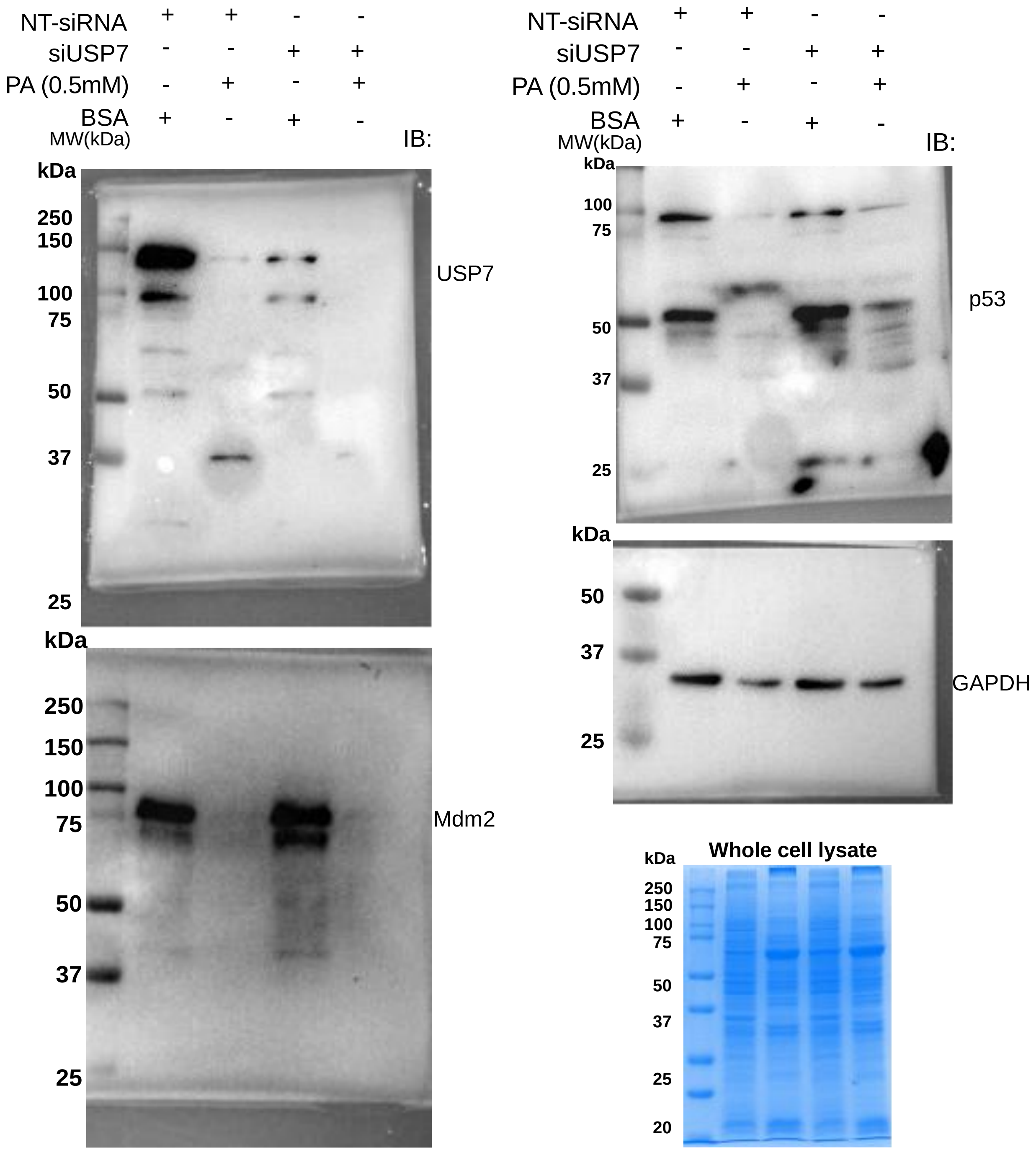
NT-siRNA + + - -
siUSP7 - - + +
PA (0.5mM) - + - +
BSA + - + -
MW(kDa)
IB:
kDa
250
150
100
75
50
37
25
USP7
kDa
250
150
100
75
50
37
25
Mdm2
NT-siRNA + + - -
siUSP7 - - + +
PA (0.5mM) - + - +
BSA + - + -
MW(kDa)
IB:
kDa
100
75
50
37
25
p53
kDa
50
37
25
GAPDH
Whole cell lysate
kDa
250
150
100
75
50
37
25
20

Figure: 3F

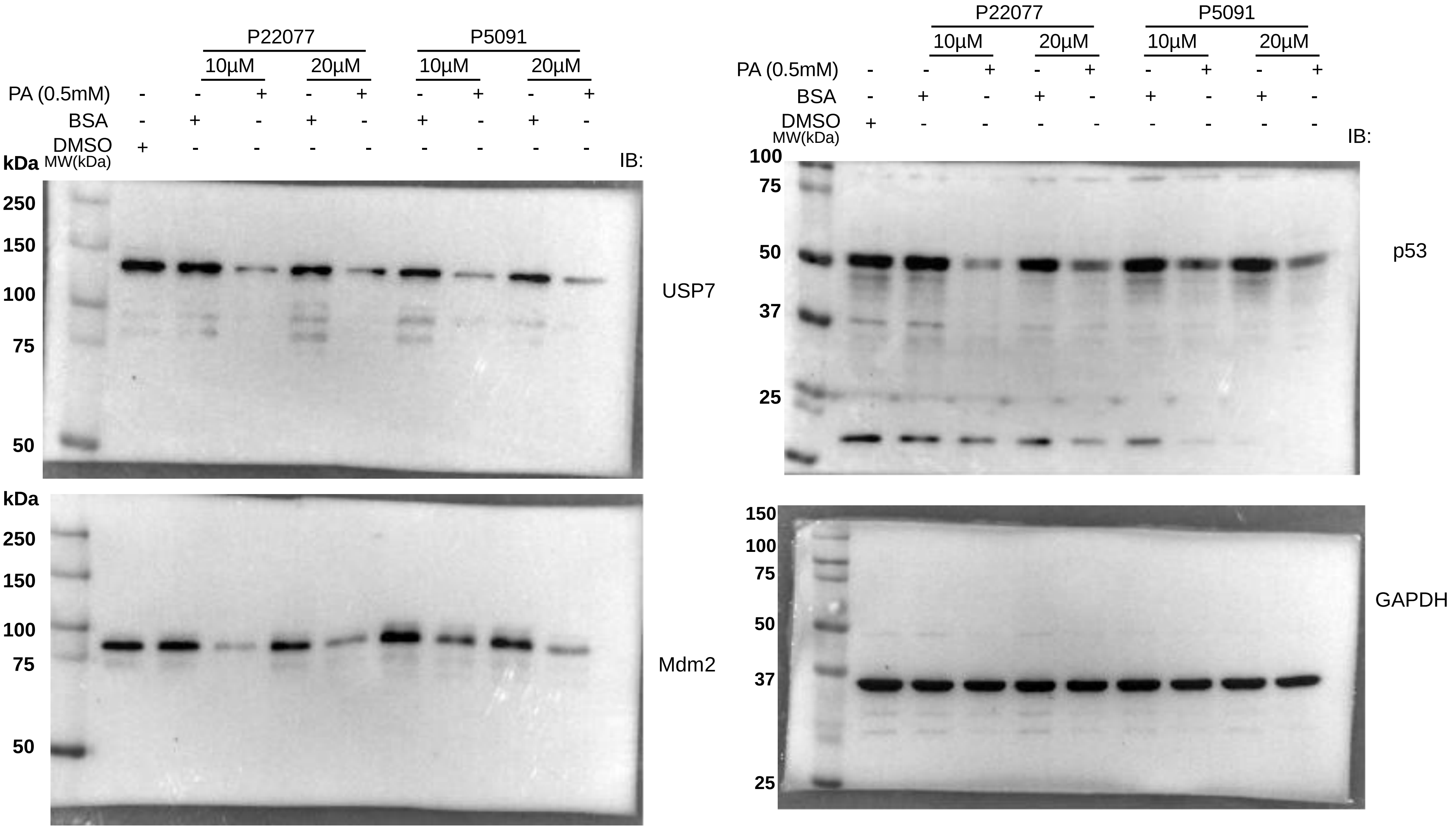
P22077
P5091
10µM
20µM
10µM
20µM
PA (0.5mM)
BSA
DMSO
kDa
MW(kDa)
IB:
250
150
100
75
50
USP7
Mdm2
p53
GAPDH
37
25

Figure: 3G

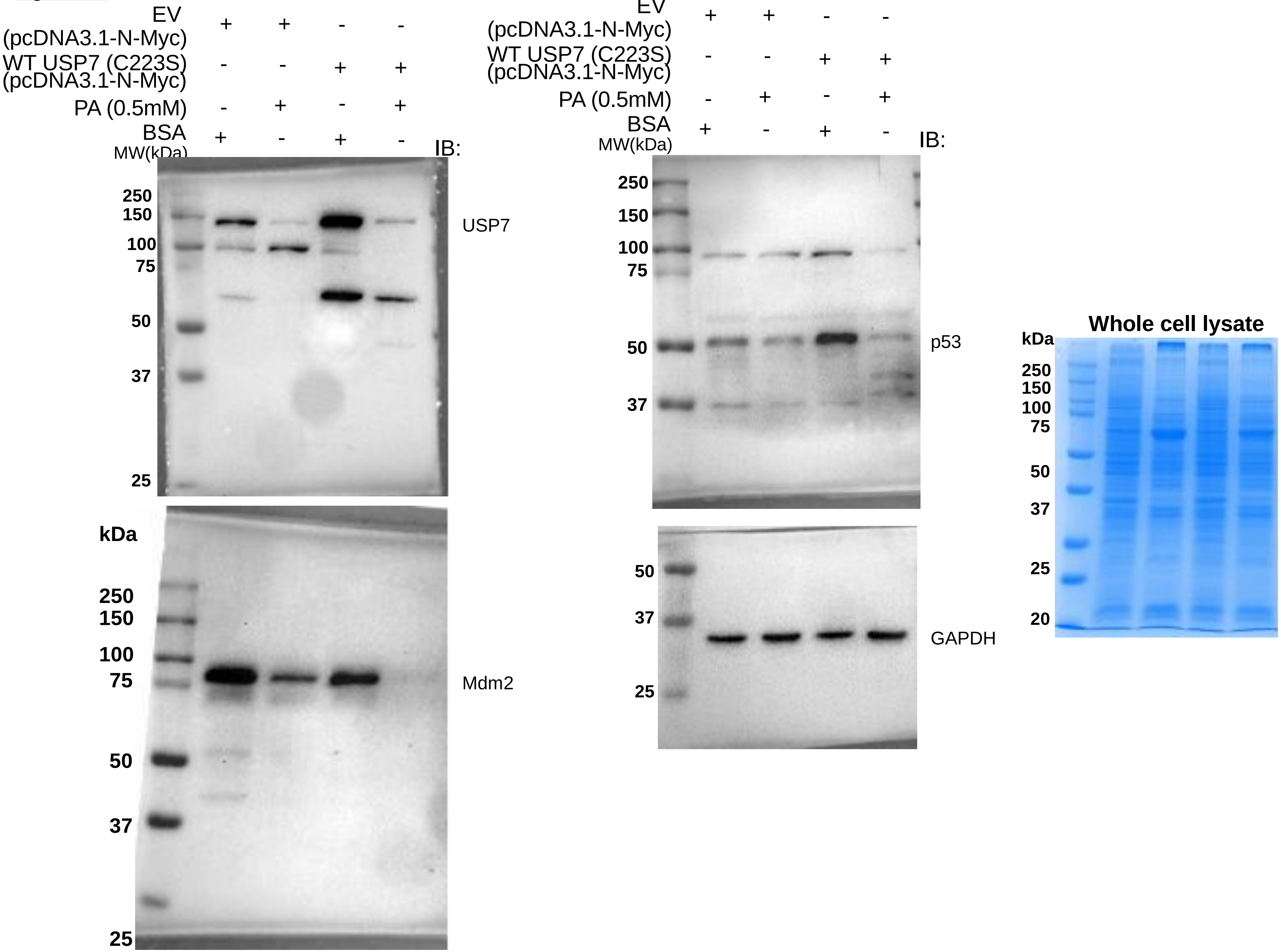
EV (pcDNA3.1-N-Myc) + + - -
WT USP7 (C223S) (pcDNA3.1-N-Myc) - - + +
PA (0.5mM) - + - +
BSA + - + -
MW(kDa)
IB:
250
150
100
75
50
37
25
USP7
kDa
Mdm2
p53
GAPDH
Whole cell lysate
20

Figure:4A

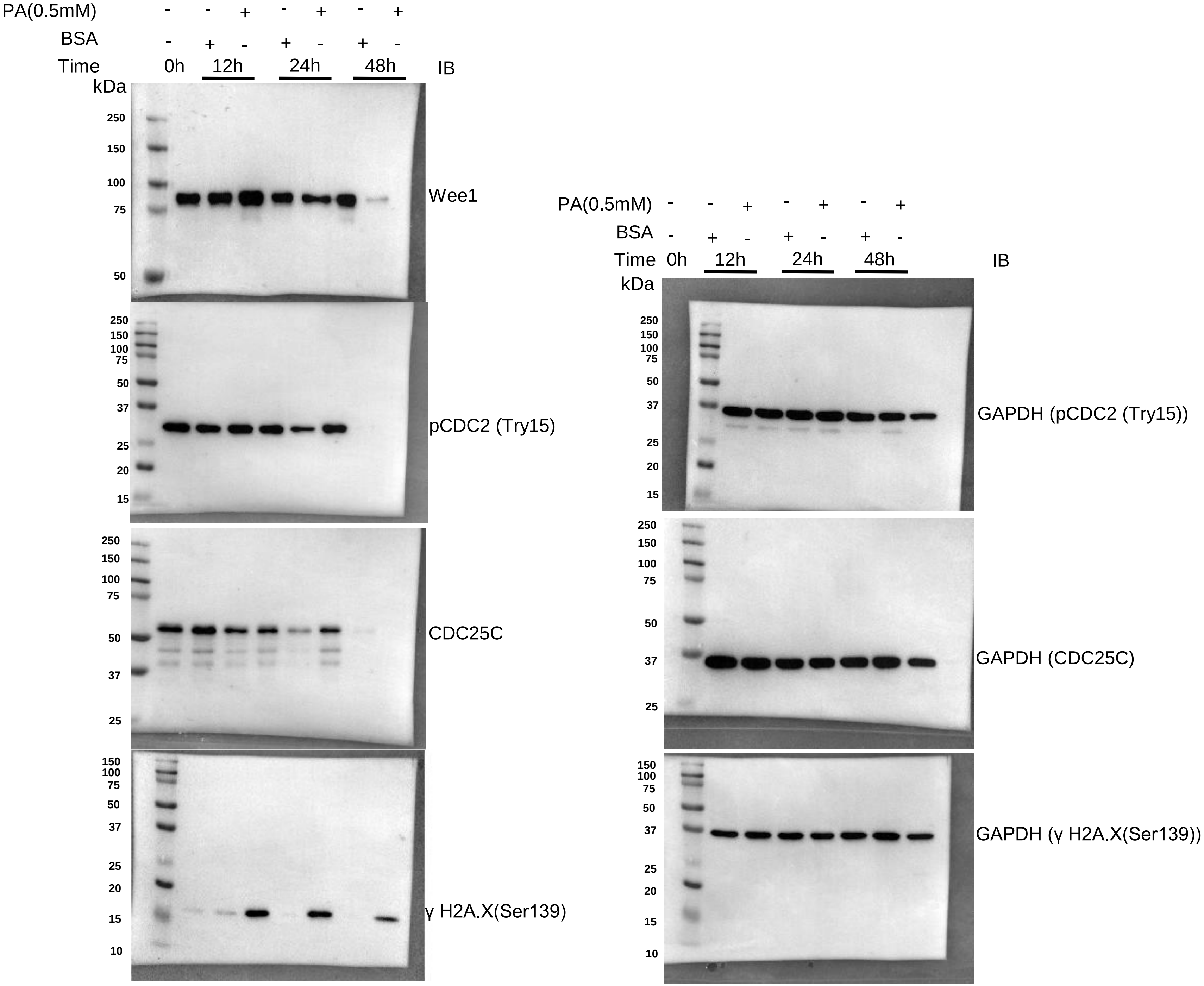
PA(0.5mM) - - + - + - +
BSA - + - + - + -
Time 0h 12h 24h 48h
IB
kDa
250
150
100
75
50
Wee1
37
25
20
15
pCDC2 (Try15)
CDC25C
10
γ H2A.X(Ser139)
GAPDH (pCDC2 (Try15))
GAPDH (CDC25C)
GAPDH (γ H2A.X(Ser139))

Figure:4A

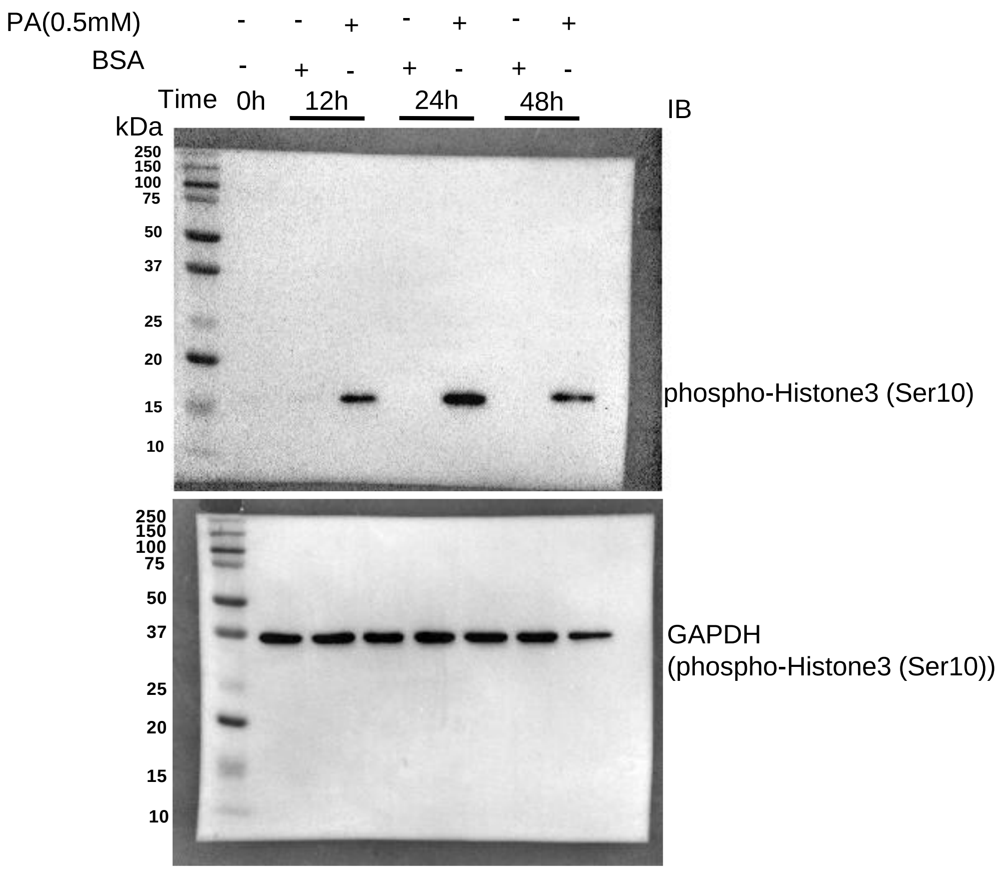
PA(0.5mM) - - + - + - +
BSA - + - + - + -
Time 0h 12h 24h 48h
IB
kDa
250
150
100
75
50
37
25
20
15
10
phospho-Histone3 (Ser10)
GAPDH
(phospho-Histone3 (Ser10))

Figure:7B

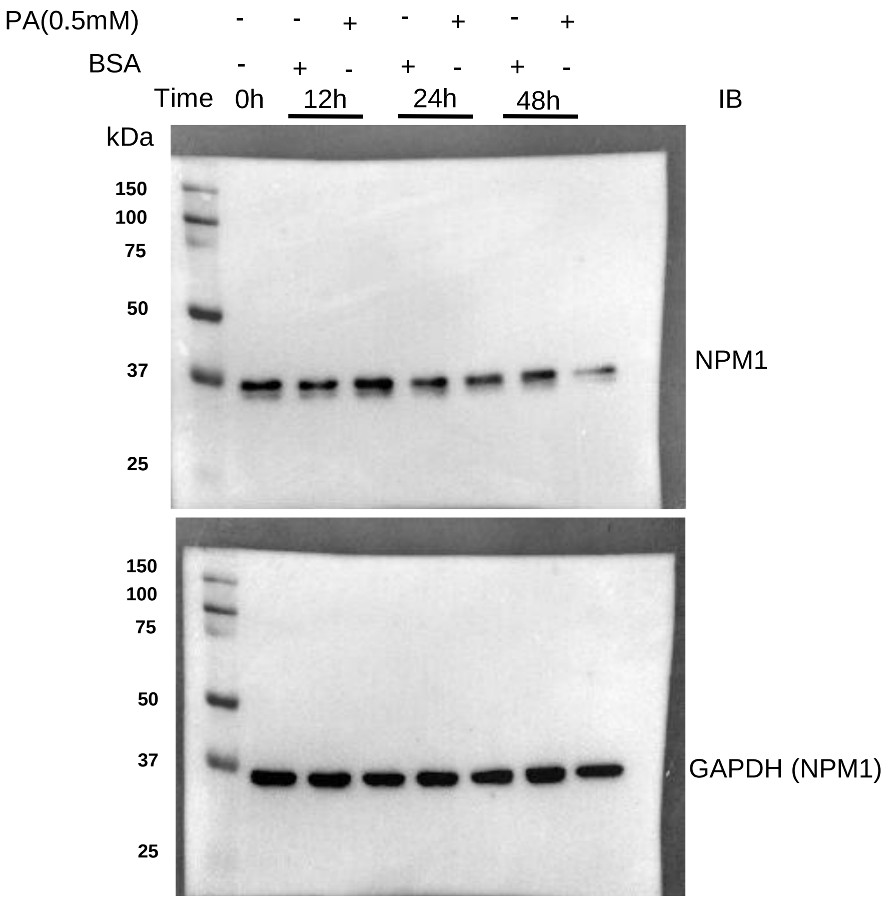

PA(0.5mM) - - + - + - +
BSA - + - + - + -
Time 0h 12h 24h 48h
IB
kDa
150
100
75
50
37
25
NPM1
150
100
75
50
37
25
GAPDH (NPM1)

Figure:8A

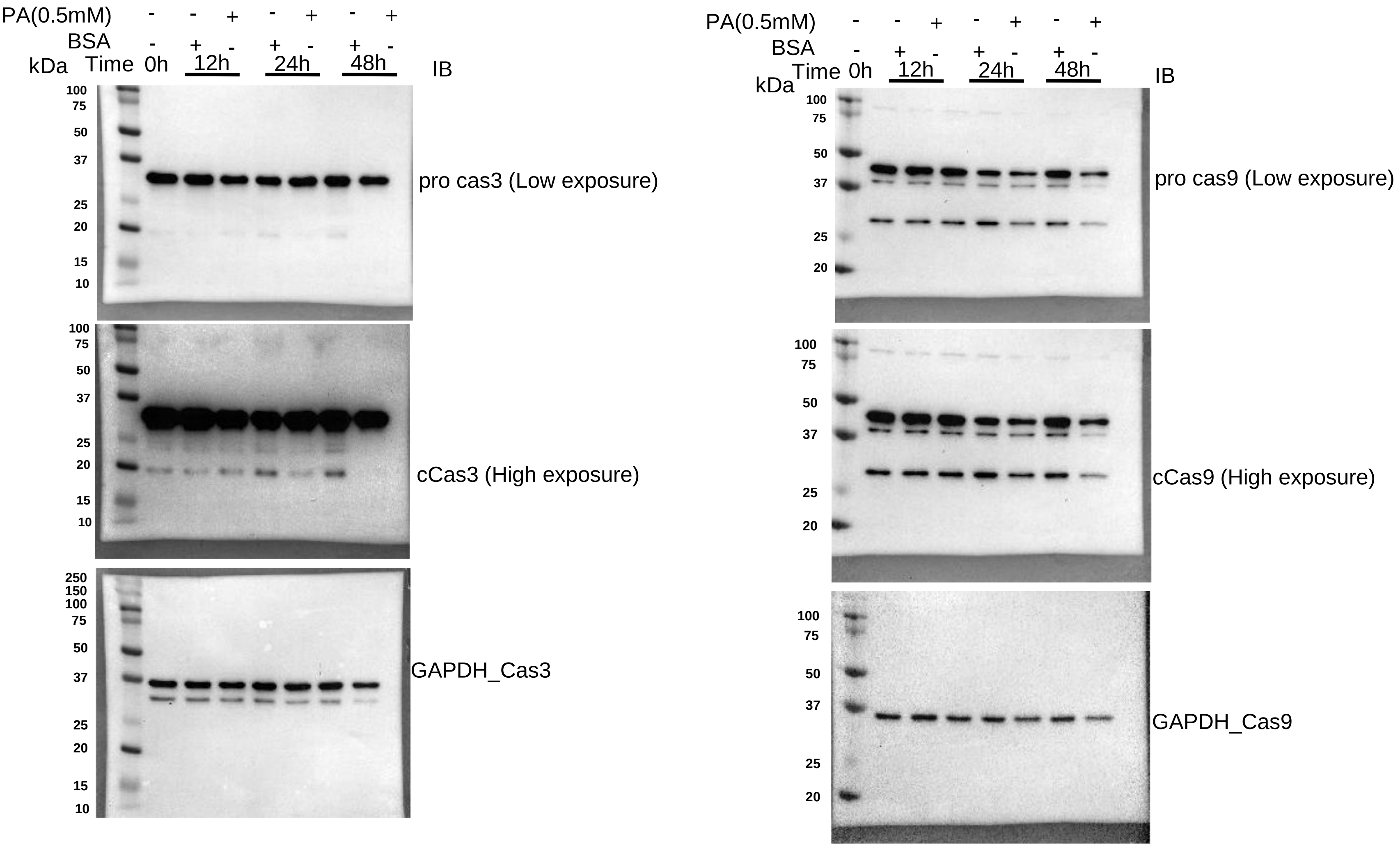

PA(0.5mM)
BSA
kDa
Time
0h
12h
24h
48h
IB
pro cas3 (Low exposure)
cCas3 (High exposure)
GAPDH_Cas3
pro cas9 (Low exposure)
cCas9 (High exposure)
GAPDH_Cas9
250
150
100
75
50
37
25
20
15
10

Figure:8A

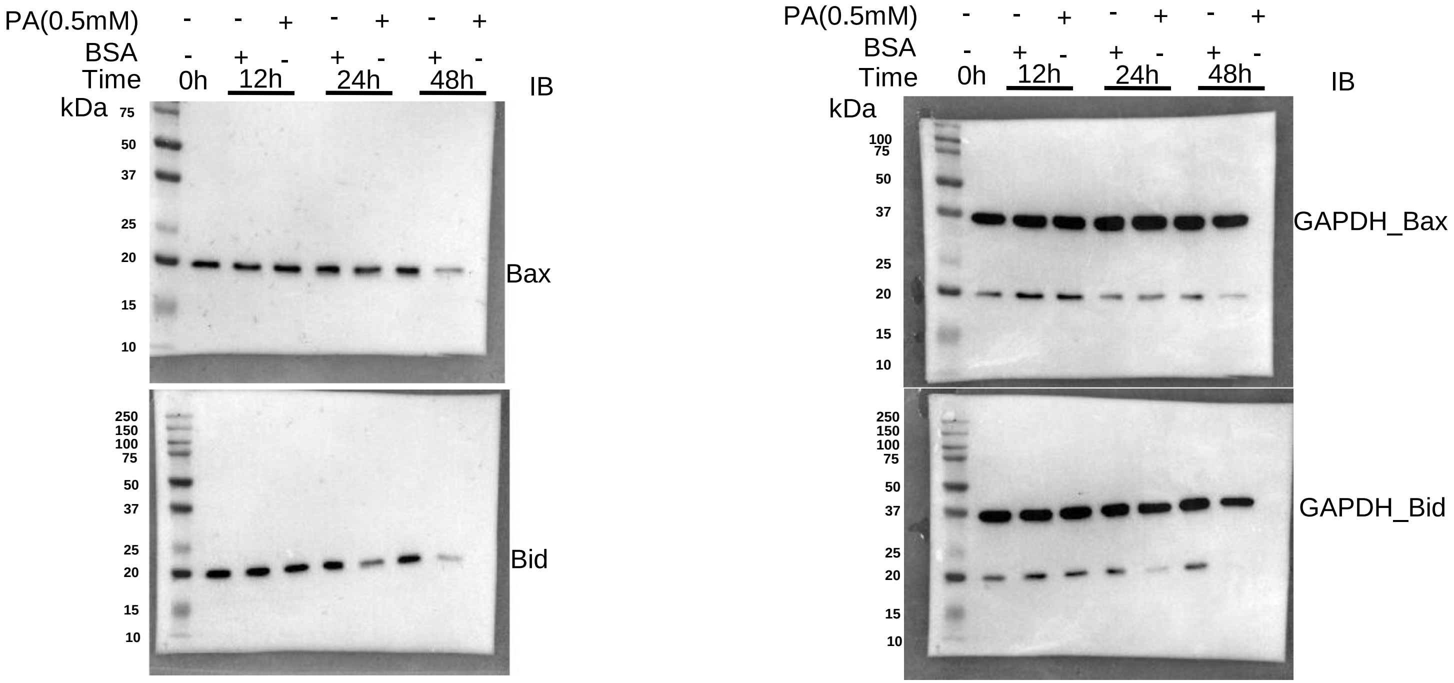
PA(0.5mM) - - + - + - +
BSA - + - + - + -
Time 0h 12h 24h 48h
IB
kDa
75 50 37 25 20 15 10
Bax
250 150 100 75 50 37 25 20 15 10
Bid
PA(0.5mM) - - + - + - +
BSA - + - + - + -
Time 0h 12h 24h 48h
IB
kDa
100 75 50 37 25 20 15 10
GAPDH_Bax
250 150 100 75 50 37 25 20 15 10
GAPDH_Bid

Figure:8A

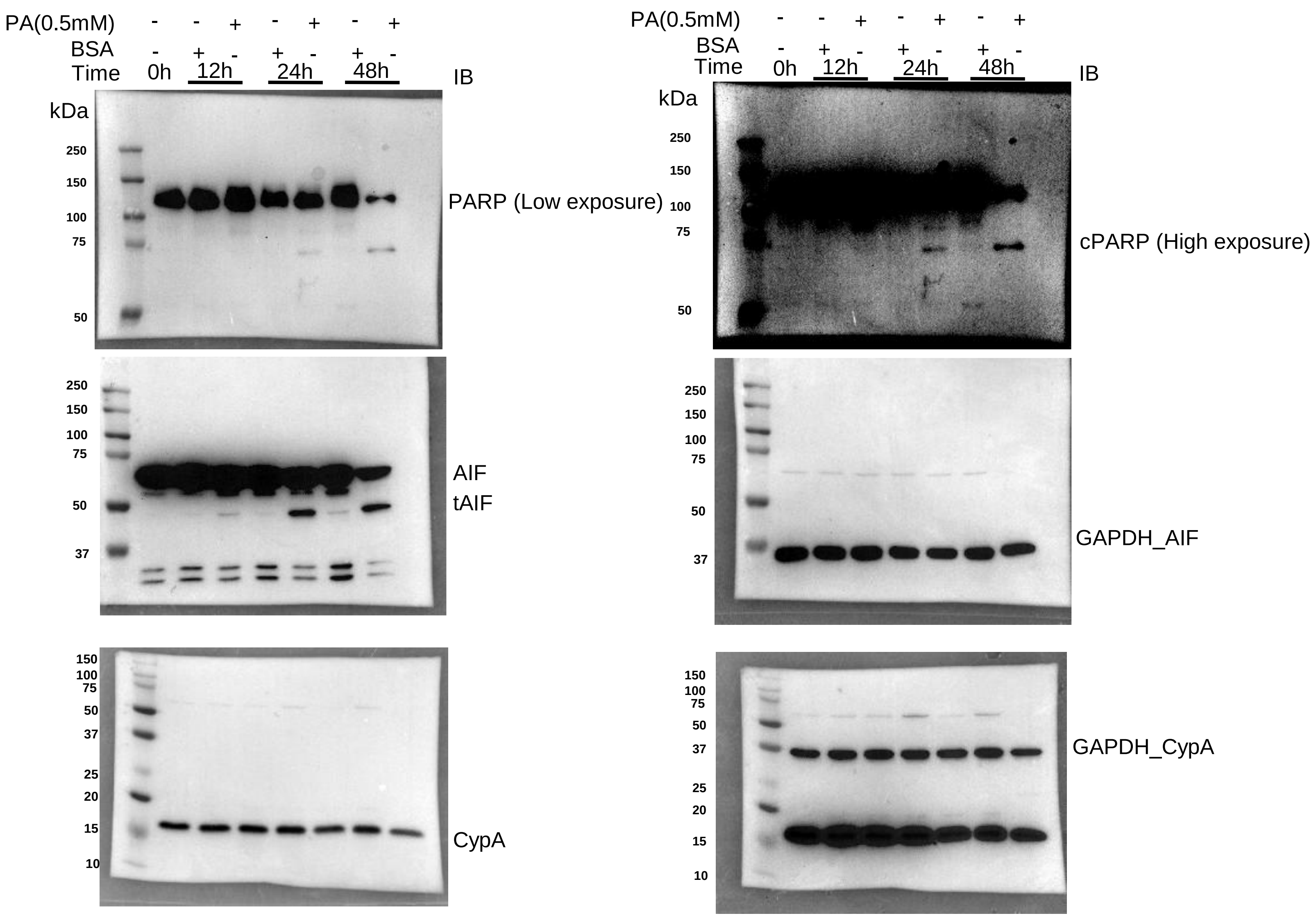
PA(0.5mM) - - + - + - +
BSA - + - + - + -
Time 0h 12h 24h 48h
IB
kDa
250
150
100
75
50
PARP (Low exposure)
cPARP (High exposure)
37
AIF
tAIF
GAPDH_AIF
25
20
15
10
CypA
GAPDH_CypA

Figure:8F

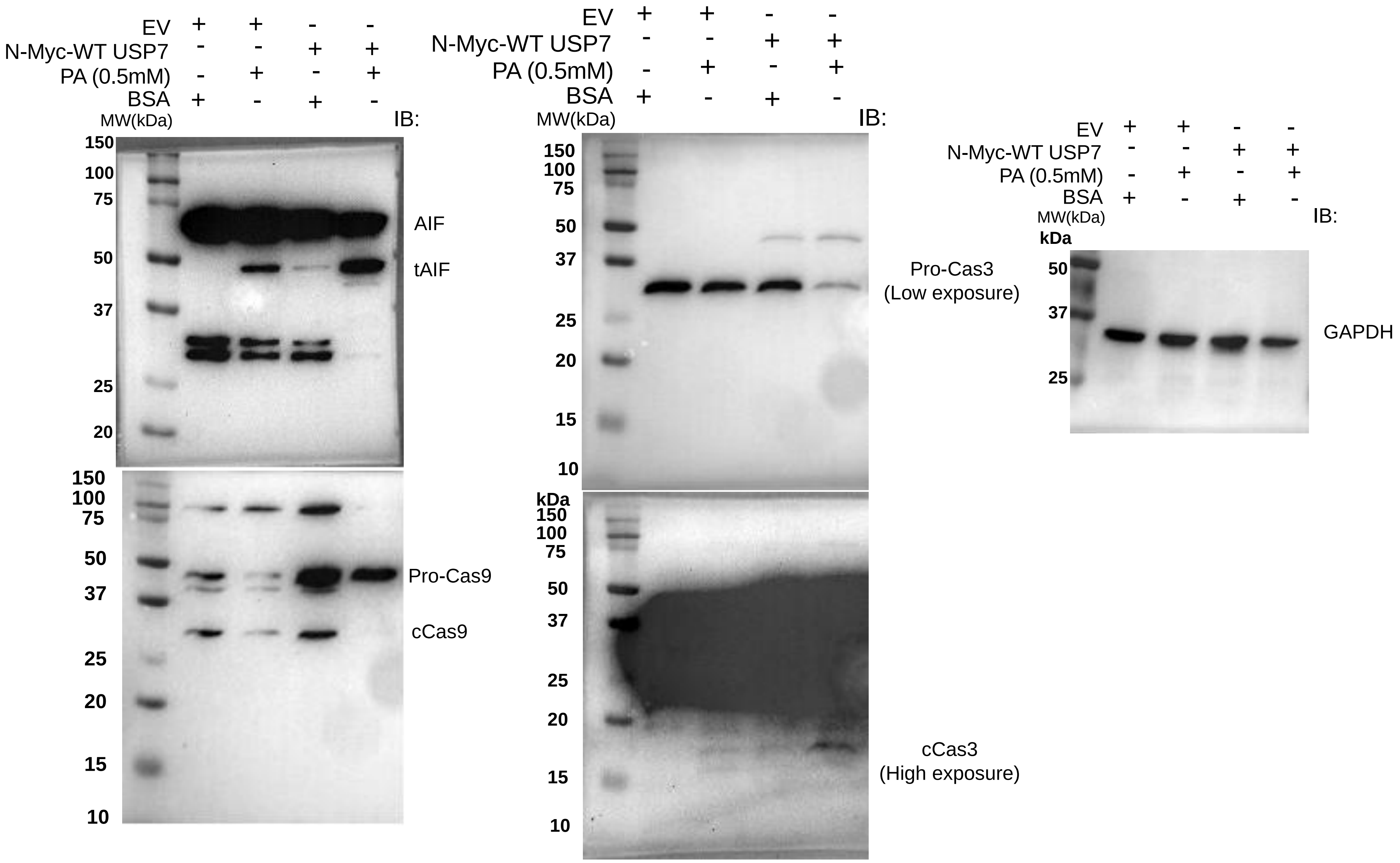
EV + + - -
N-Myc-WT USP7 - - + +
PA (0.5mM) - + - +
BSA + - + -
MW(kDa)
IB:
150
100
75
50
37
25
20
AIF
tAIF
150
100
75
50
37
25
20
15
10
Pro-Cas9
cCas9
EV + + - -
N-Myc-WT USP7 - - + +
PA (0.5mM) - + - +
BSA + - + -
MW(kDa)
IB:
150
100
75
50
37
25
20
15
10
Pro-Cas3
(Low exposure)
kDa
150
100
75
50
37
25
20
15
10
cCas3
(High exposure)
EV + + - -
N-Myc-WT USP7 - - + +
PA (0.5mM) - + - +
BSA + - + -
MW(kDa)
IB:
kDa
50
37
25
GAPDH

Figure:8G

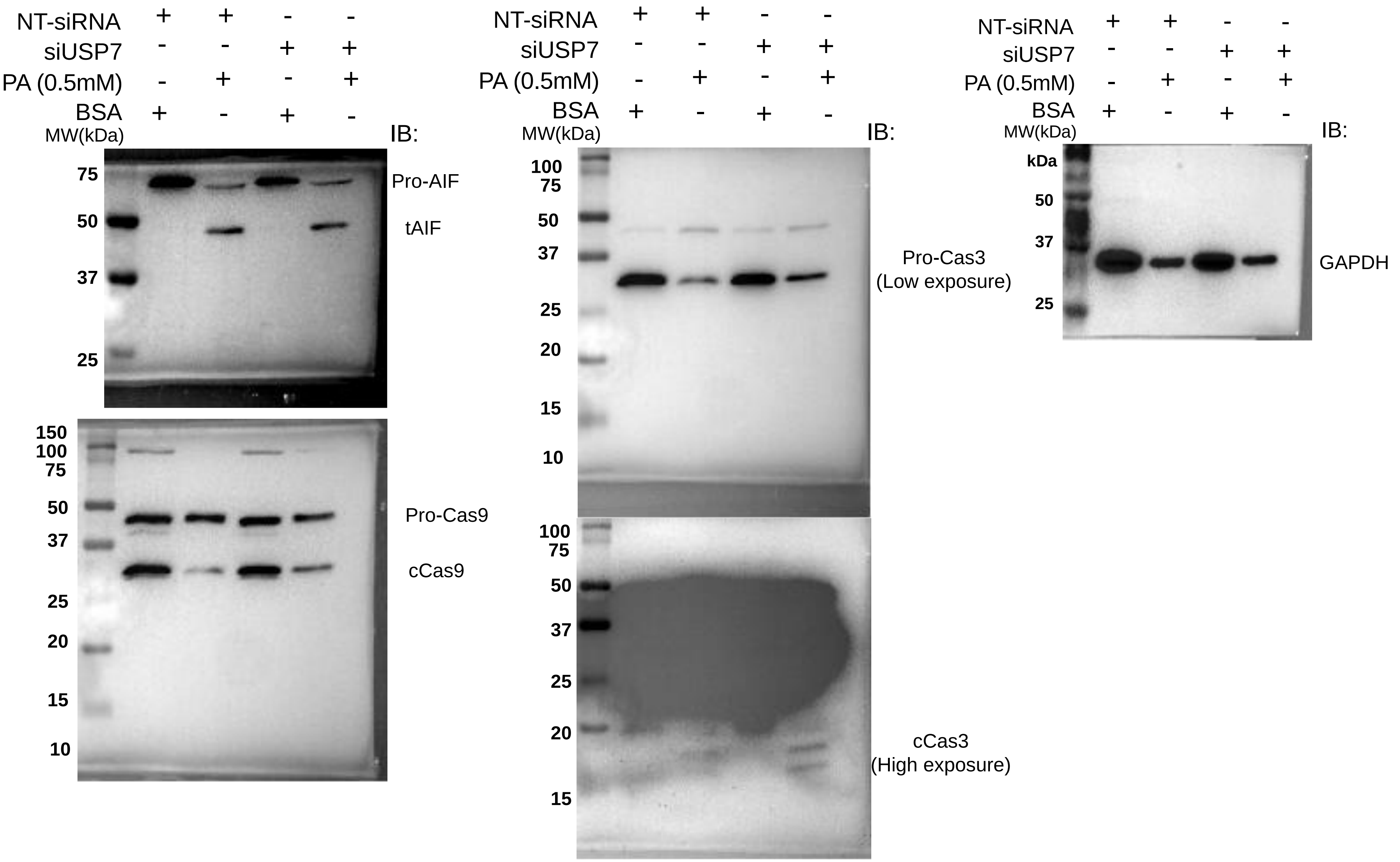
NT-siRNA + + - -
siUSP7 - - + +
PA (0.5mM) - + - +
BSA + - + -
MW(kDa)
IB:
75
50
37
25
Pro-AIF
tAIF
150
100
75
50
37
25
20
15
10
Pro-Cas9
cCas9
NT-siRNA + + - -
siUSP7 - - + +
PA (0.5mM) - + - +
BSA + - + -
MW(kDa)
IB:
100
75
50
37
25
20
15
10
Pro-Cas3
(Low exposure)
100
75
50
37
25
20
15
cCas3
(High exposure)
NT-siRNA + + - -
siUSP7 - - + +
PA (0.5mM) - + - +
BSA + - + -
MW(kDa)
IB:
kDa
50
37
25
GAPDH

Figure:8H

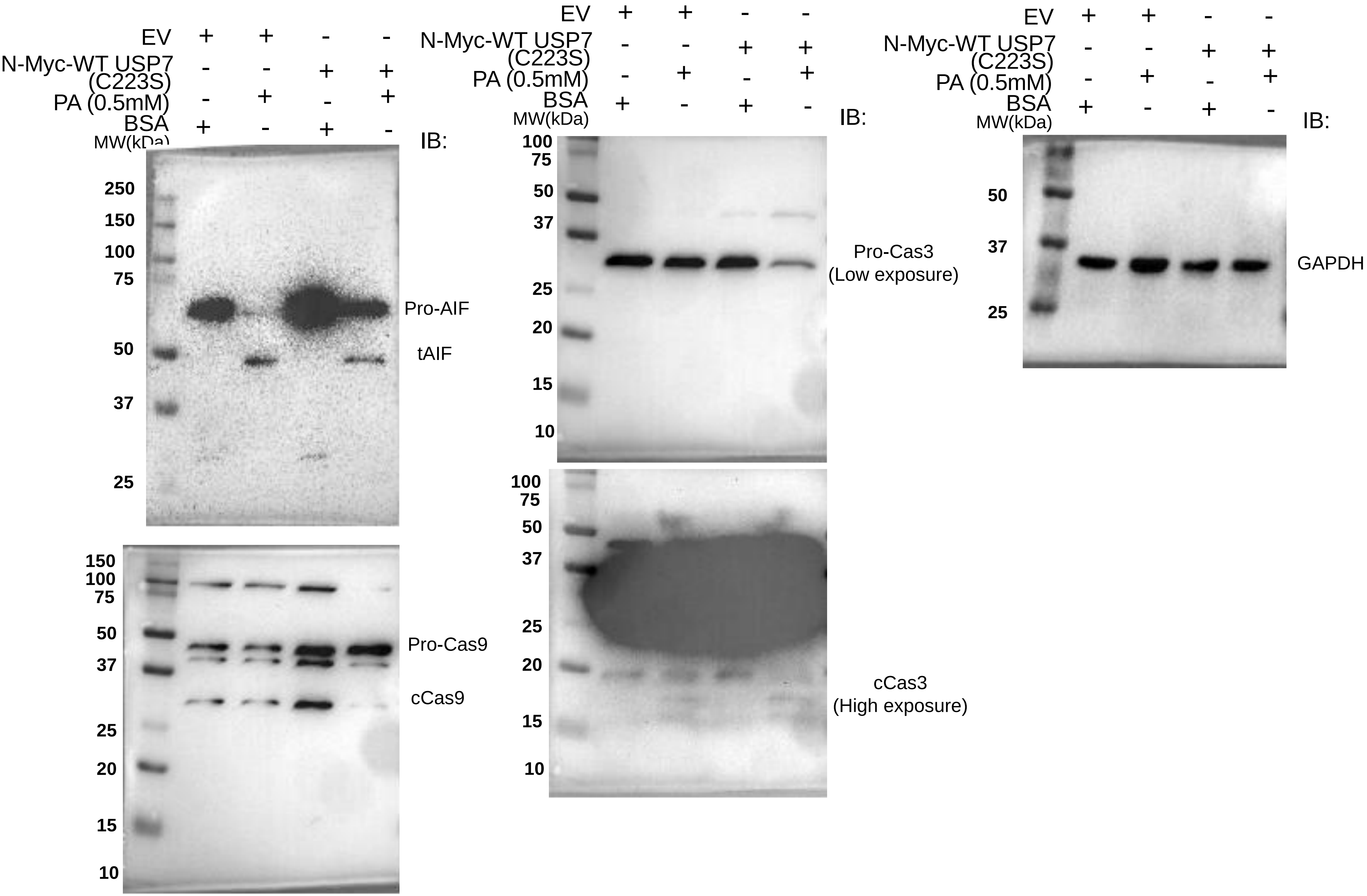
EV + + - -
N-Myc-WT USP7 (C223S) - - + +
PA (0.5mM) - + - +
BSA + - + -
MW(kDa)
IB:
250
150
100
75
50
37
25
Pro-AIF
tAIF
150
100
75
50
37
25
20
15
10
Pro-Cas9
cCas9
100
75
50
37
25
20
15
10
Pro-Cas3
(Low exposure)
cCas3
(High exposure)
50
37
25
GAPDH

Figure:8I

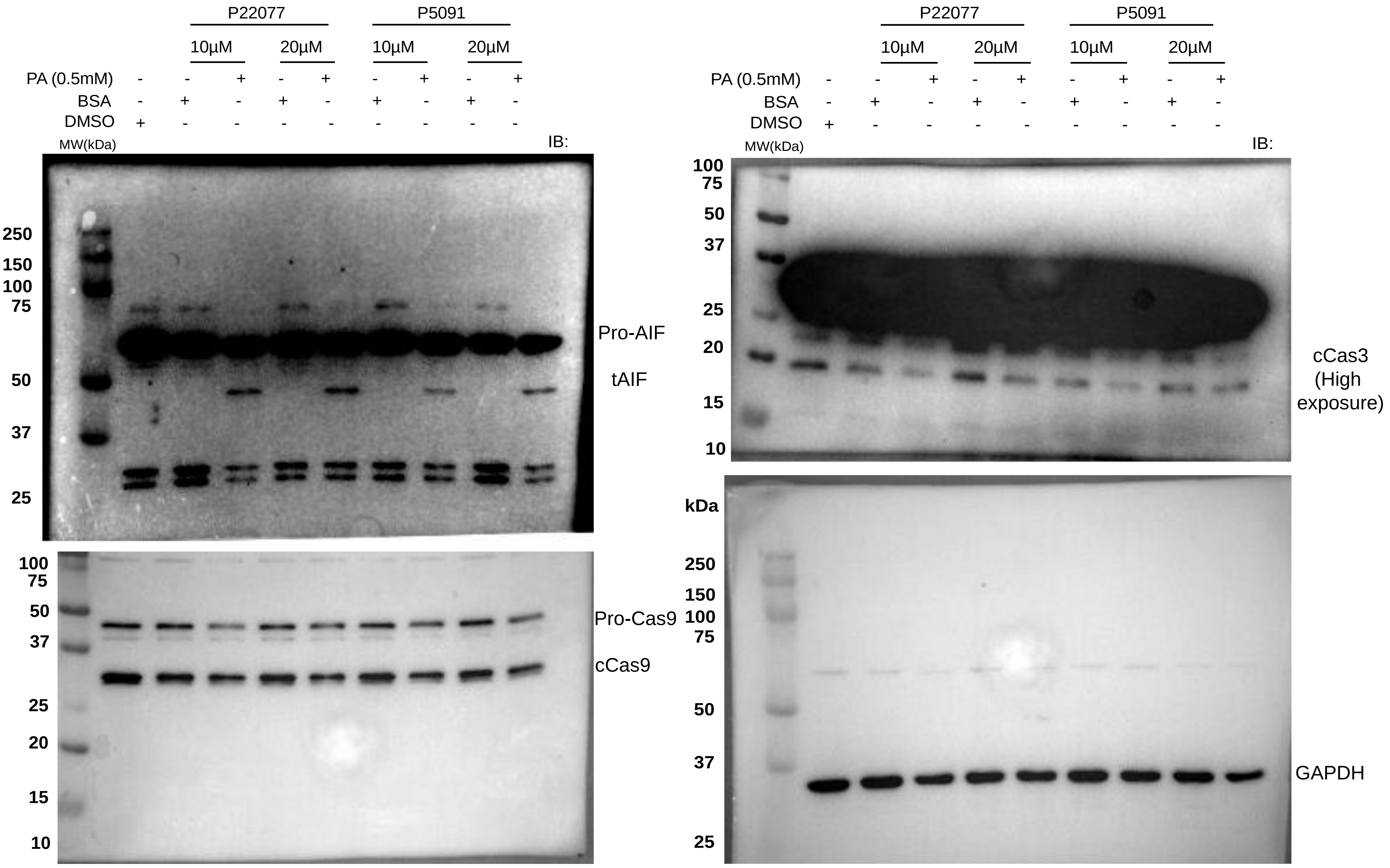
P22077
P5091
10µM
20µM
10µM
20µM
PA (0.5mM) - - + - + - + - +
BSA - + - + - + - + -
DMSO + - - - - - - - -
MW(kDa)
IB:
250
150
100
75
50
37
25
Pro-AIF
tAIF
100
75
50
37
25
20
15
10
Pro-Cas9
cCas9
P22077
P5091
10µM
20µM
10µM
20µM
PA (0.5mM) - - + - + - + - +
BSA - + - + - + - + -
DMSO + - - - - - - - -
MW(kDa)
IB:
100
75
50
37
25
20
15
10
cCas3
(High
exposure)
kDa
250
150
100
75
50
37
25
GAPDH

Figure:9B

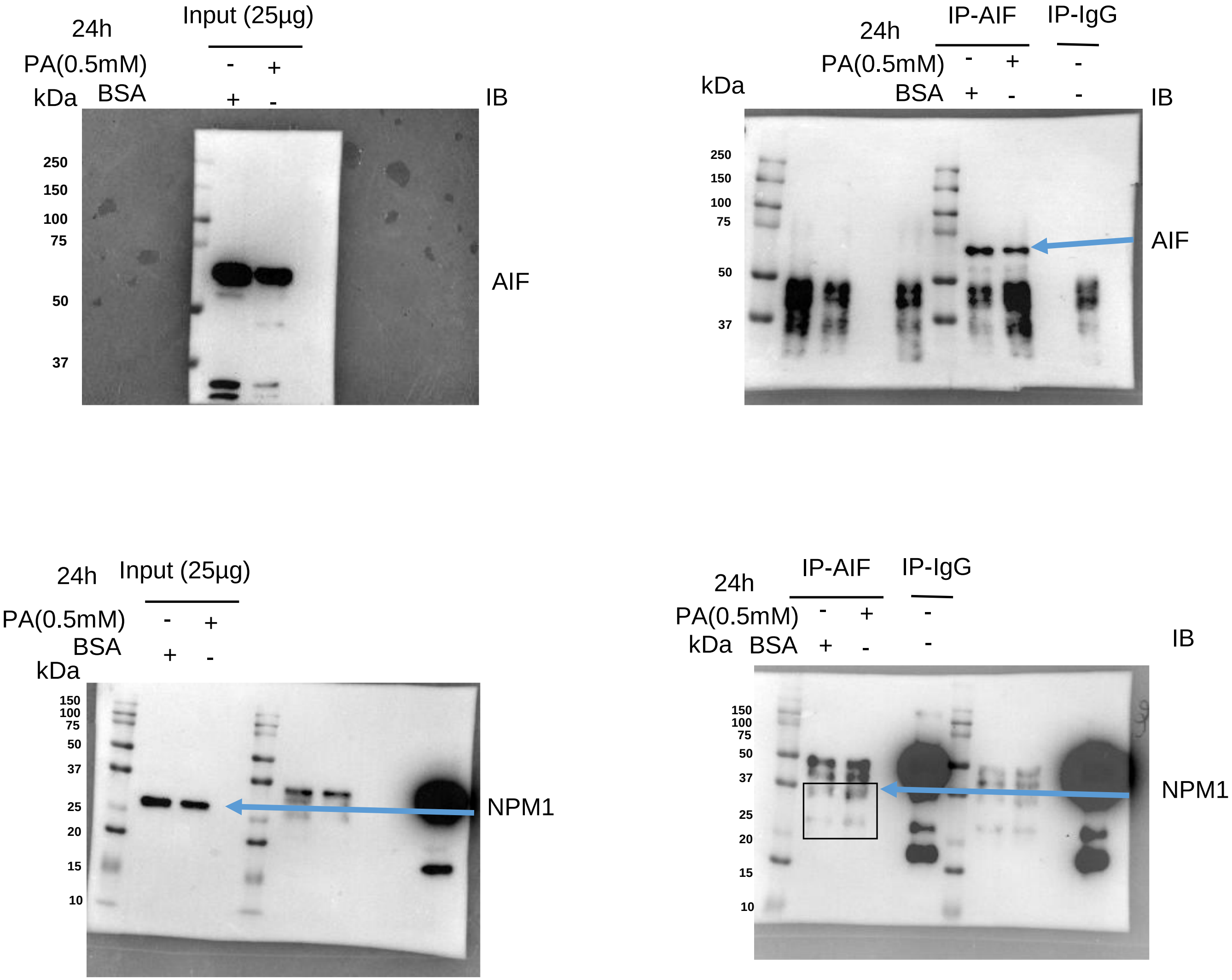

24h
Input (25µg)
PA(0.5mM) - +
kDa BSA + -
IB
250
150
100
75
50
37
AIF
24h
IP-AIF
IP-IgG
PA(0.5mM) - + -
kDa
BSA + - -
IB
250
150
100
75
50
37
AIF
24h Input (25µg)
PA(0.5mM) - +
BSA + -
kDa
150
100
75
50
37
25
20
15
10
NPM1
24h
IP-AIF
IP-IgG
PA(0.5mM) - + -
kDa BSA + - -
IB
150
100
75
50
37
25
20
15
10
NPM1

Figure:9B

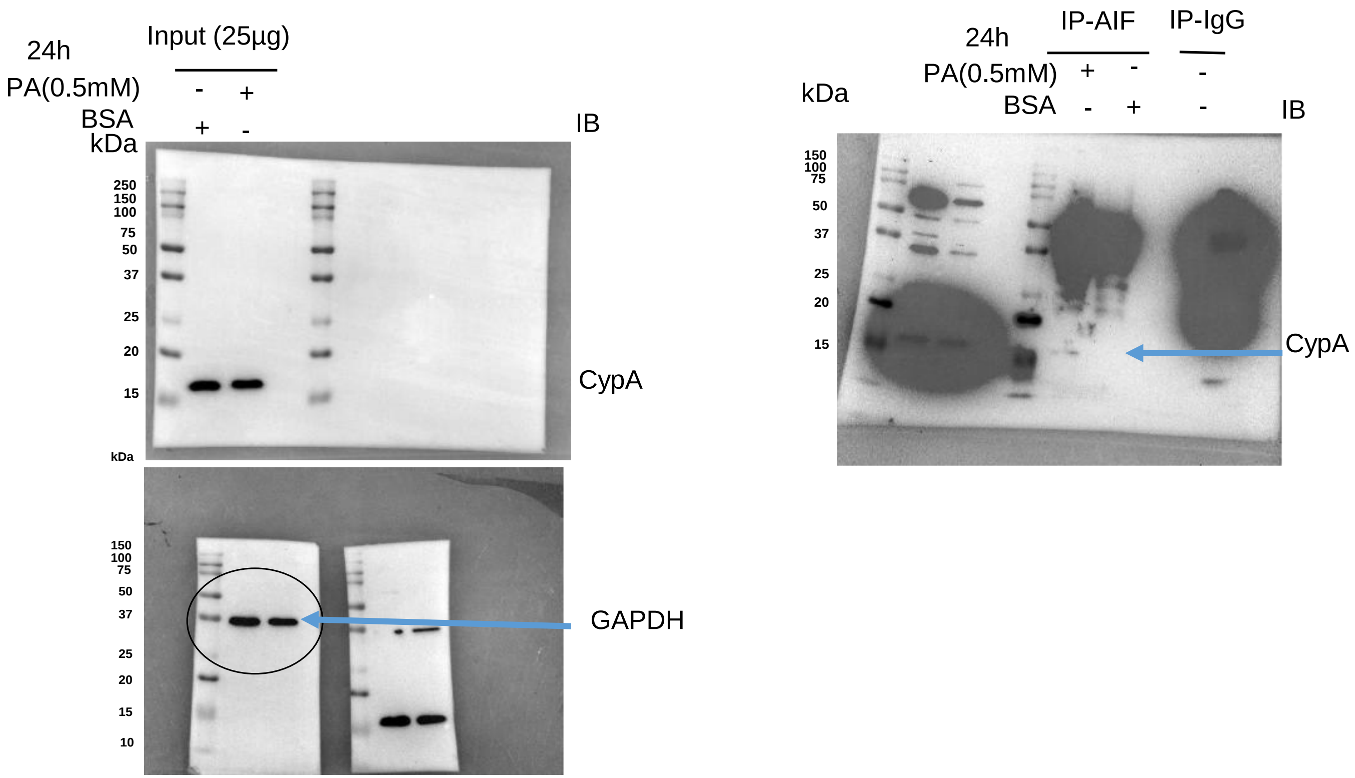
24h
Input (25µg)
PA(0.5mM) - +
BSA + -
kDa
IB
250
150
100
75
50
37
25
20
15
CypA
kDa
150
100
75
50
37
25
20
15
10
GAPDH
24h
IP-AIF
IP-IgG
PA(0.5mM) + - -
BSA - + -
kDa
IB
150
100
75
50
37
25
20
15
CypA
