# Supplementary figures and images for: Proteomic analysis reveals USP7 as a novel regulator of palmitic acid-induced hepatocellular carcinoma cell death
Source: Cell Death Dis. 2022 Jun 22;13(6):563. doi: 10.1038/s41419-022-05003-4 (PMC9217975; doi:10.1038/s41419-022-05003-4)

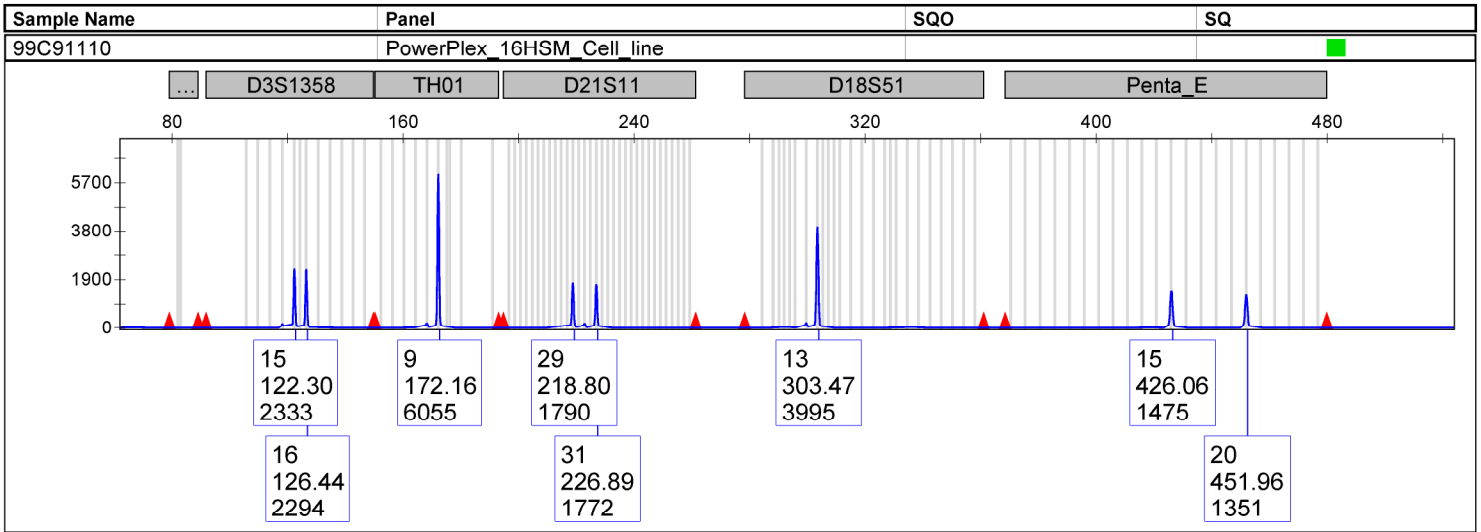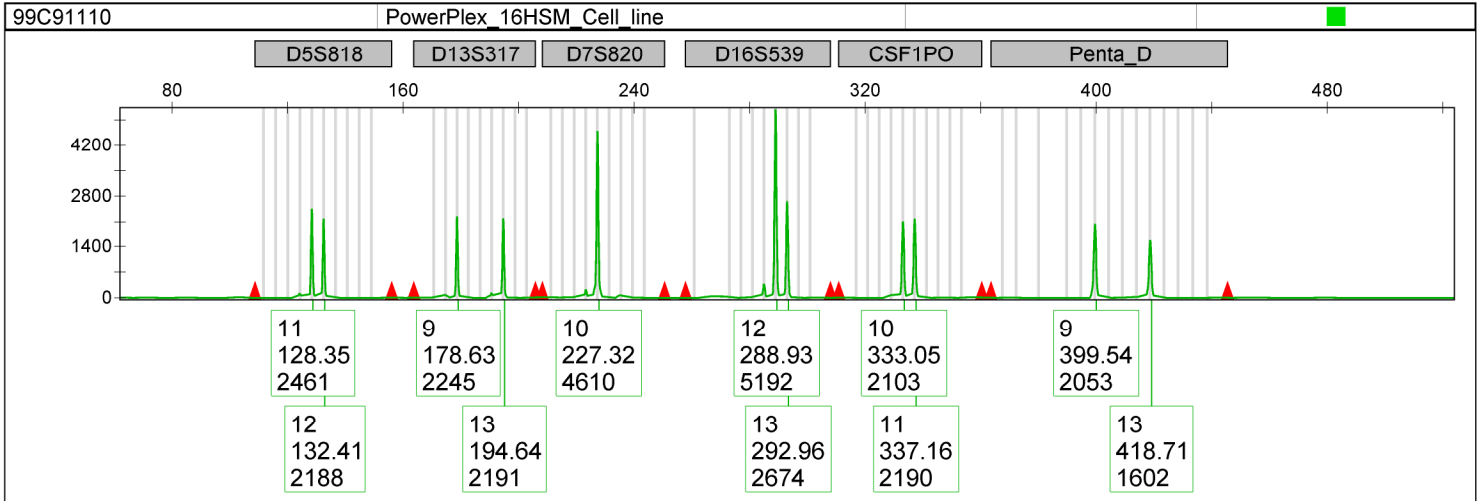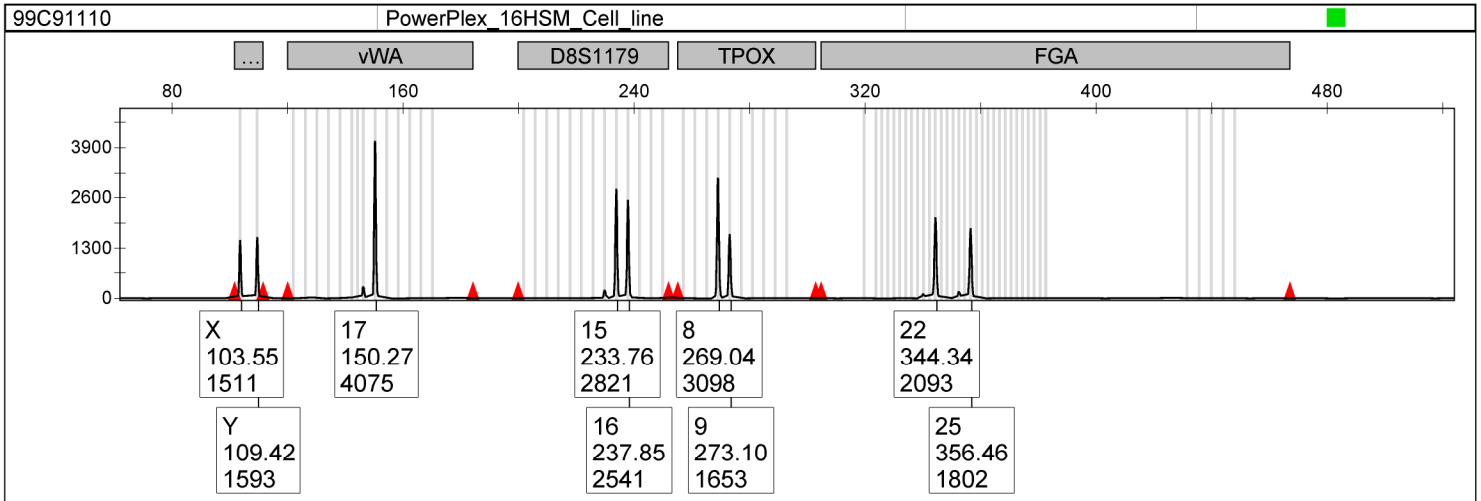

Supplement: Supplementary file 8 — Cell line authentication-STR [file 41419_2022_5003_MOESM8_ESM.pdf]
