# Supplementary material for: Proteomic analysis reveals USP7 as a novel regulator of palmitic acid-induced hepatocellular carcinoma cell death
Source: Cell Death Dis. 2022 Jun 22;13(6):563. doi: 10.1038/s41419-022-05003-4 (PMC9217975; doi:10.1038/s41419-022-05003-4)
Supplement: Supplementary file 9 — Cell Line Authentication Report_RCB_HepG2 [file 41419_2022_5003_MOESM9_ESM.pdf]

DNA Forensics Laboratory Pvt. Ltd.

C-40 A/9, Behind Community Hall, Kishangarh, Vasant Kunj, New Delhi- 110070,

Cell Line Authentication STR Profile Report

Sample Submitted By: Regional Center of Biotechnology
Address: tkmaiti@rcb.res.in
Date Sample Received: 09-09-19

Case Ref: DFL190712
Cell line Designation: HepG2
Report Date: 19-09-19

Test Result for submitted Sample			Reference Database Profile	
**Loci**	**Query profile (HepG2)**		**Database Profile (HepG2)**	
D3S1358	15	16	15	16
TH01	7	10	7	10
D21S11	29	31	29	31
D18S51	13		13	14
PentaE	15	20	15	20
D5S818	11	12	11	12
D13S317	9	13	9	13
D7S820	10		10	
D16S539	12	13	12	13
CSF1PO	10	11	10	11
PentaD	9	13	9	13
vWA	17		17	
D8S1179	15	16	15	16
TPOX	8	9	8	9
FGA	22	25	22	25
Amelogein	X	Y	X	X
**No of shared allele between query sample and database profile- 27**				
**Total no of allele in database profile- 28**				
**Percentage Match Between the submitted sample and database profile- 98.1**				

Note: *Loci highlighted in grey can be made public to verify cell identity. In order to protect the identity of the donor* ***Please do not publish*** *the allele calls from all the STR loci tested.*

DNA Forensics Laboratory Pvt. Ltd.

C-40 A/9, Behind Community Hall, Kishangarh, Vasant Kunj, New Delhi- 110070,

Interpretation of Result:

Cell lines with ≥80% match are considered to be related; i.e., derived from a common ancestry.

☐ The submitted sample profile is human, but not a match for any profile in the STR database

☐ The submitted sample profile is an exact match for the following human cell line in the STR database (8 core loci plus Amelogenin)

■ The submitted sample **(Hep-G2)** profile is similar to the following human cell line in STR Database: **(Hep-G2)**

Comparative output from Database:

Accession	Name	Nº Markers	Score	Amel	CSF1PO	D2S1338	D3S1358	D5S818	D7S820	D8S1179	D13S317	D16S539	D18S51	D19S433	D21S11	FGA	Penta D	Penta E	TH01	TPOX	vWA
NA	Query	NA	NA	X,Y	10,11		15,16	11,12	10	15,16	9,13	12,13	13		29,31	22,25	9,13	15,20	9	8,9	17
CVCL_0027	Hep-G2	15	98.11%	X,Y	10,11	19,20	15,16	11,12	10	15,16	9,13	12,13	13,14	15.2	29,31	22,25	9,13	15,20	9	8,9	17
CVCL_1098	Hep-G2/C3A	15	94.34%	X,Y	10,11		15,16	11,13	10	15,16	9,13	12,13	13,14		29,31	22,25	9,13	15,20	9	8,9	17
CVCL_1D66 *Best*	U-CH10	15	61.22%	X	10,11	19	15,16	11	10	8,13	9,13	11,13	13	13,15	29,31	24	9,13	18	8,9	11	16
CVCL_1D66 *Worst*	U-CH10	15	60.00%	X	10,11	19	15,16	11	9,10	8,13	9,13	11,13	13	13,15	29,31	24	9,13	18	8,9	11	16
CVCL_VR21	SK-RC-34	12	63.16%	X	11	16	16	11,12	10,12	13,16		12,13	15	13,14	29	21			9.3	8,9	14,17

Disclaimer: STR data Reported relate only to the samples received and tested at our NABL, ILAC/MRA an ISO15189:2012 testing laboratory. Authentication of cell line has been performed based on available cell bank databases. A minimum of eight core STR loci plus amelogenin for sex determination (X or XY) are required for cell line authentication.

End of Report
