# Supplementary material for: Proteomic analysis reveals USP7 as a novel regulator of palmitic acid-induced hepatocellular carcinoma cell death
Source: Cell Death Dis. 2022 Jun 22;13(6):563. doi: 10.1038/s41419-022-05003-4 (PMC9217975; doi:10.1038/s41419-022-05003-4)
Supplement: Supplementary file 10 — Supplementary Table 1 [file 41419_2022_5003_MOESM10_ESM.pdf]

Serial No	Accession #	Description	Gene name	Fold change (PA treated/BSA control)
1	sp|Q13501|SQSTM_HUMAN	Sequestosome-1	SQSTM1	1.995
2	sp|Q99541|PLIN2_HUMAN	Perilipin-2	PLIN2	1.674
3	sp|P18615|NELFE_HUMAN	Negative elongation factor E	NELFE	1.613
4	sp|Q14764|MVP_HUMAN	Major vault protein	MVP	1.581
5	sp|Q99714|HCD2_HUMAN	3-hydroxyacyl-CoA dehydrogenase type-2	HSD17B10	1.555
6	sp|Q01844|EWS_HUMAN	RNA-binding protein EWS	EWSR1	1.533
7	sp|Q9NX58|LYAR_HUMAN	Cell growth-regulating nucleolar protein	LYAR	1.531
8	sp|Q07955|SRSF1_HUMAN	Serine/arginine-rich splicing factor 1	SRSF1	1.529
9	sp|P18583|SON_HUMAN	Protein SON	SON	1.528
10	sp|P02671|FIBA_HUMAN	Fibrinogen alpha chain	FGA	1.517
11	sp|P06748|NPM_HUMAN	Nucleophosmin	NPM1	1.517
12	sp|Q9BRP8|PYM1_HUMAN	Partner of Y14 and mago	PYM1	1.510
13	sp|Q15637|SF01_HUMAN	Splicing factor 1	SF1	1.507
14	sp|O94760|DDAH1_HUMAN	N(G),N(G)-dimethylarginine dimethylaminohydrolase 1	DDAH1	1.505
15	sp|A0MZ66|SHOT1_HUMAN	Shootin-1	SHTN1	1.505
16	sp|P19404|NDUV2_HUMAN	NADH dehydrogenase [ubiquinone] flavoprotein 2, mitochondrial	NDUFV2	1.504
17	sp|Q9BRF8|CPPED_HUMAN	Serine/threonine-protein phosphatase CPPED1	CPPED1	1.502
18	sp|P10606|COX5B_HUMAN	Cytochrome c oxidase subunit 5B, mitochondrial	COX5B	1.500
19	sp|P62805|H4_HUMAN	Histone H4	HIST1H4A	1.500
20	sp|Q15050|RRS1_HUMAN	Ribosome biogenesis regulatory protein homolog	RRS1	1.496
21	sp|Q9UKY7|CDV3_HUMAN	Protein CDV3 homolog	CDV3	1.495
22	sp|P30049|ATPD_HUMAN	ATP synthase subunit delta, mitochondrial	ATP5F1D	1.494
23	sp|P30038|AL4A1_HUMAN	Delta-1-pyrroline-5-carboxylate dehydrogenase, mitochondrial	ALDH4A1	1.494
24	sp|P04181|OAT_HUMAN	Ornithine aminotransferase, mitochondrial	OAT	1.491
25	sp|P16949|STMN1_HUMAN	Stathmin	STMN1	1.489
26	sp|P17096|HMGA1_HUMAN	High mobility group protein HMG-I/HMG-Y	HMGA1	1.486
27	sp|P67809|YBOX1_HUMAN	Nuclease-sensitive element-binding protein 1	YBX1	1.485
28	sp|Q13405|RM49_HUMAN	39S ribosomal protein L49, mitochondrial	MRPL49	1.485
29	sp|Q16630|CPSF6_HUMAN	Cleavage and	CPSF6	1.479

		polyadenylation specificity factor subunit 6		
30	sp|O00330|ODPX_HUMAN	Pyruvate dehydrogenase protein X component, mitochondrial	PDHX	1.476
31	sp|P99999|CYC_HUMAN	Cytochrome c	CYCS	1.474
32	sp|Q99729|ROAA_HUMAN	Heterogeneous nuclear ribonucleoprotein A/B	HNRNPAB	1.473
33	sp|O75494|SRS10_HUMAN	Serine/arginine-rich splicing factor 10	SRSF10	1.473
34	sp|O43290|SNUT1_HUMAN	U4/U6.U5 tri-snRNP-associated protein 1	SART1	1.472
35	sp|P35637|FUS_HUMAN	RNA-binding protein FUS	FUS	1.468
36	sp|P54819|KAD2_HUMAN	Adenylate kinase 2, mitochondrial	AK2	1.467
37	sp|Q13428|TCOF_HUMAN	Treacle protein	TCOF1	1.464
38	sp|P20810|ICAL_HUMAN	Calpastatin	CAST	1.464
39	sp|O96008|TOM40_HUMAN	Mitochondrial import receptor subunit TOM40 homolog	TOMM40	1.459
40	sp|P62979|RS27A_HUMAN	Ubiquitin-40S ribosomal protein S27a	RPS27A	1.457
41	sp|P09429|HMGB1_HUMAN	High mobility group protein B1	HMGB1	1.454
42	sp|Q9Y3U8|RL36_HUMAN	60S ribosomal protein L36	RPL36	1.453
43	sp|P62917|RL8_HUMAN	60S ribosomal protein L8	RPL8	1.453
44	sp|Q1KMD3|HNRL2_HUMAN	Heterogeneous nuclear ribonucleoprotein U-like protein 2	HNRNPUL2	1.452
45	sp|P22307|NLTP_HUMAN	Non-specific lipid-transfer protein	SCP2	1.451
46	sp|Q8WW12|PCNP_HUMAN	PEST proteolytic signal-containing nuclear protein	PCNP	1.449
47	sp|P10768|ESTD_HUMAN	S-formylglutathione hydrolase	ESD	1.447
48	sp|Q92979|NEP1_HUMAN	Ribosomal RNA small subunit methyltransferase NEP1	EMG1	1.446
49	sp|P30085|KCY_HUMAN	UMP-CMP kinase	CMPK1	1.441
50	sp|Q9NW13|RBM28_HUMAN	RNA-binding protein 28	RBM28	1.439
51	sp|P05362|ICAM1_HUMAN	Intercellular adhesion molecule 1	ICAM1	1.439
52	sp|Q04837|SSBP_HUMAN	Single-stranded DNA-binding protein, mitochondrial	SSBP1	1.438
53	sp|O14979|HNRDL_HUMAN	Heterogeneous nuclear ribonucleoprotein D-like	HNRNPDL	1.438
54	sp|P37198|NUP62_HUMAN	Nuclear pore glycoprotein p62	NUP62	1.438
55	sp|P35754|GLRX1_HUMAN	Glutaredoxin-1	GLRX	1.438

56	sp|Q99848|EBP2_HUMAN	Probable rRNA-processing protein EBP2	EBNA1BP2	1.437
57	sp|P05455|LA_HUMAN	Lupus La protein	SSB	1.436
58	sp|P06753|TPM3_HUMAN	Tropomyosin alpha-3 chain	TPM3	1.436
59	sp|P02649|APOE_HUMAN	Apolipoprotein E	APOE	1.435
60	sp|P51858|HDGF_HUMAN	Hepatoma-derived growth factor	HDGF	1.435
61	sp|Q86UP2|KTN1_HUMAN	Kinectin	KTN1	1.435
62	sp|Q9UHX1|PUF60_HUMAN	Poly(U)-binding-splicing factor PUF60	PUF60	1.434
63	sp|P55084|ECHB_HUMAN	Trifunctional enzyme subunit beta, mitochondrial	HADHB	1.433
64	sp|P20674|COX5A_HUMAN	Cytochrome c oxidase subunit 5A, mitochondrial	COX5A	1.433
65	sp|P62258|1433E_HUMAN	14-3-3 protein epsilon	YWHAE	1.430
66	sp|P18621|RL17_HUMAN	60S ribosomal protein L17	RPL17	1.428
67	sp|P42330|AK1C3_HUMAN	Aldo-keto reductase family 1 member C3	AKR1C3	1.427
68	sp|P16989|YBOX3_HUMAN	Y-box-binding protein 3	YBX3	1.427
69	sp|O43390|HNRPR_HUMAN	Heterogeneous nuclear ribonucleoprotein R	HNRNPR	1.426
70	sp|P18859|ATP5J_HUMAN	ATP synthase-coupling factor 6, mitochondrial	ATP5J	1.425
71	sp|Q14103|HNRPD_HUMAN	Heterogeneous nuclear ribonucleoprotein D0	HNRNPD	1.424
72	sp|Q9UMX0|UBQL1_HUMAN	Ubiquilin-1	UBQLN1	1.423
73	sp|P10515|ODP2_HUMAN	Dihydrolipoyllysine-residue acetyltransferase component of pyruvate dehydrogenase complex, mitochondrial	DLAT	1.423
74	sp|O76021|RL1D1_HUMAN	Ribosomal L1 domain-containing protein 1	RSL1D1	1.422
75	sp|P23588|IF4B_HUMAN	Eukaryotic translation initiation factor 4B	EIF4B	1.422
76	sp|Q9BVP2|GNL3_HUMAN	Guanine nucleotide-binding protein-like 3	GNL3	1.421
77	sp|Q13435|SF3B2_HUMAN	Splicing factor 3B subunit 2	SF3B2	1.420
78	sp|O00515|LAD1_HUMAN	Ladinin-1	LAD1	1.420
79	sp|O95793|STAU1_HUMAN	Double-stranded RNA-binding protein Staufen homolog 1	STAU1	1.420
80	sp|P49411|EFTU_HUMAN	Elongation factor Tu, mitochondrial	TUFM	1.420
81	sp|P62750|RL23A_HUMAN	60S ribosomal protein L23a	RPL23A	1.418
82	sp|Q9BXP5|SRRT_HUMAN	Serrate RNA effector molecule homolog	SRRT	1.418
83	sp|P19338|NUCL_HUMAN	Nucleolin	NCL	1.417
84	sp|Q15942|ZYX_HUMAN	Zyxin	ZYX	1.416
85	sp|P08621|RU17_HUMAN	U1 small nuclear	SNRNP70	1.416

		ribonucleoprotein 70 kDa		
86	sp|P28074|PSB5_HUMAN	Proteasome subunit beta type-5	PSMB5	1.415
87	sp|Q9NYL9|TMOD3_HUMAN	Tropomodulin-3	TMOD3	1.415
88	sp|Q03154|ACY1_HUMAN	Aminoacylase-1	ACY1	1.414
89	sp|Q9UNZ2|NSF1C_HUMAN	NSFL1 cofactor p47	NSFL1C	1.413
90	sp|P30044|PRDX5_HUMAN	Peroxiredoxin-5, mitochondrial	PRDX5	1.413
91	sp|P37108|SRP14_HUMAN	Signal recognition particle 14 kDa protein	SRP14	1.411
92	sp|P09104|ENOG_HUMAN	Gamma-enolase	ENO2	1.411
93	sp|P50454|SERPH_HUMAN	Serpin H1	SERPINH1	1.411
94	sp|Q9NZL9|MAT2B_HUMAN	Methionine adenosyltransferase 2 subunit beta	MAT2B	1.410
95	sp|Q13268|DHRS2_HUMAN	Dehydrogenase/reductase SDR family member 2, mitochondrial	DHRS2	1.410
96	sp|Q8WWI1|LMO7_HUMAN	LIM domain only protein 7	LMO7	1.409
97	sp|P62277|RS13_HUMAN	40S ribosomal protein S13	RPS13	1.408
98	sp|P28331|NDUS1_HUMAN	NADH-ubiquinone oxidoreductase 75 kDa subunit, mitochondrial	NDUFS1	1.408
99	sp|P35232|PHB_HUMAN	Prohibitin	PHB	1.407
100	sp|Q05682|CALD1_HUMAN	Caldesmon	CALD1	1.407
101	sp|P06576|ATPB_HUMAN	ATP synthase subunit beta, mitochondrial	ATP5F1B	1.406
102	sp|P22626|ROA2_HUMAN	Heterogeneous nuclear ribonucleoproteins A2/B1	HNRNPA2B1	1.405
103	sp|Q9UKK9|NUDT5_HUMAN	ADP-sugar pyrophosphatase	NUDT5	1.404
104	sp|P38646|GRP75_HUMAN	Stress-70 protein, mitochondrial	HSPA9	1.404
105	sp|Q99798|ACON_HUMAN	Aconitate hydratase, mitochondrial	ACO2	1.403
106	sp|P36957|ODO2_HUMAN	Dihydrolipoyllysine-residue succinyltransferase component of 2-oxoglutarate dehydrogenase complex, mitochondrial	DLST	1.403
107	sp|Q16718|NDUA5_HUMAN	NADH dehydrogenase [ubiquinone] 1 alpha subcomplex subunit 5	NDUFA5	1.402
108	sp|Q92945|FUBP2_HUMAN	Far upstream element-binding protein 2	KHSRP	1.402
109	sp|Q96EY1|DNJA3_HUMAN	DnaJ homolog subfamily A member 3, mitochondrial	DNAJA3	1.402
110	sp|P38159|RBMX_HUMAN	RNA-binding motif protein, X chromosome	RBMX	1.401
111	sp|P32322|P5CR1_HUMAN	Pyrroline-5-carboxylate	PYCR1	1.401

		reductase 1, mitochondrial		
112	sp|O43823|AKAP8_HUMAN	A-kinase anchor protein 8	AKAP8	1.401
113	sp|Q15459|SF3A1_HUMAN	Splicing factor 3A subunit 1	SF3A1	1.400
114	sp|P30086|PEBP1_HUMAN	Phosphatidylethanolamine-binding protein 1	PEBP1	1.400
115	sp|Q7L014|DDX46_HUMAN	Probable ATP-dependent RNA helicase DDX46	DDX46	1.400
116	sp|P61604|CH10_HUMAN	10 kDa heat shock protein, mitochondrial	HSPE1	1.400
117	sp|P29401|TKT_HUMAN	Transketolase	TKT	1.399
118	sp|P26583|HMGB2_HUMAN	High mobility group protein B2	HMGB2	1.399
119	sp|P11177|ODPB_HUMAN	Pyruvate dehydrogenase E1 component subunit beta, mitochondrial	PDHB	1.397
120	sp|Q8NC51|PAIRB_HUMAN	Plasminogen activator inhibitor 1 RNA-binding protein	SERBP1	1.397
121	sp|P63104|1433Z_HUMAN	14-3-3 protein zeta/delta	YWHAZ	1.396
122	sp|Q99497|PARK7_HUMAN	Protein/nucleic acid deglycase DJ-1	PARK7	1.396
123	sp|Q14847|LASP1_HUMAN	LIM and SH3 domain protein 1	LASP1	1.395
124	sp|P12270|TPR_HUMAN	Nucleoprotein TPR	TPR	1.394
125	sp|Q9UKV3|ACINU_HUMAN	Apoptotic chromatin condensation inducer in the nucleus	ACIN1	1.394
126	sp|Q16543|CDC37_HUMAN	Hsp90 co-chaperone Cdc37	CDC37	1.394
127	sp|P23378|GCSP_HUMAN	Glycine dehydrogenase (decarboxylating), mitochondrial	GLDC	1.394
128	sp|P04040|CATA_HUMAN	Catalase	CAT	1.394
129	sp|P14927|QCR7_HUMAN	Cytochrome b-c1 complex subunit 7	UQCRB	1.393
130	sp|P40222|TXLNA_HUMAN	Alpha-taxilin	TXLNA	1.393
131	sp|P15559|NQO1_HUMAN	NAD(P)H dehydrogenase [quinone] 1	NQO1	1.393
132	sp|Q15181|IPYR_HUMAN	Inorganic pyrophosphatase	PPA1	1.393
133	sp|P42765|THIM_HUMAN	3-ketoacyl-CoA thiolase, mitochondrial	ACAA2	1.393
134	sp|P12955|PEPD_HUMAN	Xaa-Pro dipeptidase	PEPD	1.393
135	sp|Q9UQ80|PA2G4_HUMAN	Proliferation-associated protein 2G4	PA2G4	1.392
136	sp|Q14157|UBP2L_HUMAN	Ubiquitin-associated protein 2-like	UBAP2L	1.392
137	sp|Q12905|ILF2_HUMAN	Interleukin enhancer-binding factor 2	ILF2	1.391
138	sp|O75390|CISY_HUMAN	Citrate synthase, mitochondrial	CS	1.390

139	sp|O75947|ATP5H_HUMAN	ATP synthase subunit d, mitochondrial	ATP5H	1.390
140	sp|Q02790|FKBP4_HUMAN	Peptidyl-prolyl cis-trans isomerase FKBP4	FKBP4	1.389
141	sp|P09651|ROA1_HUMAN	Heterogeneous nuclear ribonucleoprotein A1	HNRNPA1	1.389
142	sp|Q13151|ROA0_HUMAN	Heterogeneous nuclear ribonucleoprotein A0	HNRNPA0	1.389
143	sp|Q02878|RL6_HUMAN	60S ribosomal protein L6	RPL6	1.389
144	sp|Q01105|SET_HUMAN	Protein SET	SET	1.388
145	sp|P02787|TRFE_HUMAN	Serotransferrin	TF	1.388
146	sp|P24752|THIL_HUMAN	Acetyl-CoA acetyltransferase, mitochondrial	ACAT1	1.388
147	sp|Q8WXF1|PSPC1_HUMAN	Paraspeckle component 1	PSPC1	1.387
148	sp|Q9UJZ1|STML2_HUMAN	Stomatin-like protein 2, mitochondrial	STOML2	1.387
149	sp|P14314|GLU2B_HUMAN	Glucosidase 2 subunit beta	PRKCSH	1.386
150	sp|Q07021|C1QBP_HUMAN	Complement component 1 Q subcomponent-binding protein, mitochondrial	C1QBP	1.386
151	sp|Q99584|S10AD_HUMAN	Protein S100-A13	S100A13	1.386
152	sp|P08243|ASNS_HUMAN	Asparagine synthetase [glutamine-hydrolyzing]	ASNS	1.386
153	sp|P78371|TCPB_HUMAN	T-complex protein 1 subunit beta	CCT2	1.386
154	sp|P22087|FBRL_HUMAN	rRNA 2'-O-methyltransferase fibrillarin	FBL	1.385
155	sp|Q8NBS9|TXND5_HUMAN	Thioredoxin domain-containing protein 5	TXNDC5	1.384
156	sp|Q9HAV7|GRPE1_HUMAN	GrpE protein homolog 1, mitochondrial	GRPEL1	1.384
157	sp|Q9BQ61|TRIR_HUMAN	Telomerase RNA component interacting RNase	TRIR	1.383
158	sp|Q9UK22|FBX2_HUMAN	F-box only protein 2	FBXO2	1.382
159	sp|Q99459|CDC5L_HUMAN	Cell division cycle 5-like protein	CDC5L	1.381
160	sp|Q9NQP4|PFD4_HUMAN	Prefoldin subunit 4	PFDN4	1.381
161	sp|O43143|DHX15_HUMAN	Pre-mRNA-splicing factor ATP-dependent RNA helicase DHX15	DHX15	1.381
162	sp|P52758|RIDA_HUMAN	2-iminobutanoate/2-iminopropanoate deaminase	RIDA	1.381
163	sp|Q86V81|THOC4_HUMAN	THO complex subunit 4	ALYREF	1.381
164	sp|Q9UIJ7|KAD3_HUMAN	GTP:AMP phosphotransferase AK3, mitochondrial	AK3	1.380
165	sp|P43243|MATR3_HUMAN	Matrin-3	MATR3	1.380
166	sp|P52597|HNRPF_HUMAN	Heterogeneous nuclear	HNRNPF	1.379

		ribonucleoprotein F		
167	sp|P05387|RLA2_HUMAN	60S acidic ribosomal protein P2	RPLP2	1.378
168	sp|P62263|RS14_HUMAN	40S ribosomal protein S14	RPS14	1.377
169	sp|P23193|TCEA1_HUMAN	Transcription elongation factor A protein 1	TCEA1	1.376
170	sp|P56537|IF6_HUMAN	Eukaryotic translation initiation factor 6	EIF6	1.376
171	sp|P46777|RL5_HUMAN	60S ribosomal protein L5	RPL5	1.376
172	sp|Q13242|SRSF9_HUMAN	Serine/arginine-rich splicing factor 9	SRSF9	1.375
173	sp|P27816|MAP4_HUMAN	Microtubule-associated protein 4	MAP4	1.375
174	sp|P36551|HEM6_HUMAN	Oxygen-dependent coproporphyrinogen-III oxidase, mitochondrial	CPOX	1.375
175	sp|P31947|1433S_HUMAN	14-3-3 protein sigma	SFN	1.374
176	sp|P10809|CH60_HUMAN	60 kDa heat shock protein, mitochondrial	HSPD1	1.373
177	sp|Q15233|NONO_HUMAN	Non-POU domain-containing octamer-binding protein	NONO	1.373
178	sp|Q96AE4|FUBP1_HUMAN	Far upstream element-binding protein 1	FUBP1	1.373
179	sp|P51991|ROA3_HUMAN	Heterogeneous nuclear ribonucleoprotein A3	HNRNPA3	1.373
180	sp|P08133|ANXA6_HUMAN	Annexin A6	ANXA6	1.373
181	sp|P50213|IDH3A_HUMAN	Isocitrate dehydrogenase [NAD] subunit alpha, mitochondrial	IDH3A	1.372
182	sp|P26599|PTBP1_HUMAN	Polypyrimidine tract-binding protein 1	PTBP1	1.372
183	sp|P54727|RD23B_HUMAN	UV excision repair protein RAD23 homolog B	RAD23B	1.372
184	sp|Q96C23|GALM_HUMAN	Aldose 1-epimerase	GALM	1.372
185	sp|Q13162|PRDX4_HUMAN	Peroxiredoxin-4	PRDX4	1.371
186	sp|P30048|PRDX3_HUMAN	Thioredoxin-dependent peroxide reductase, mitochondrial	PRDX3	1.371
187	sp|Q9NQ50|RM40_HUMAN	39S ribosomal protein L40, mitochondrial	MRPL40	1.370
188	sp|P49321|NASP_HUMAN	Nuclear autoantigenic sperm protein	NASP	1.370
189	sp|P30084|ECHM_HUMAN	Enoyl-CoA hydratase, mitochondrial	ECHS1	1.370
190	sp|P15311|EZRI_HUMAN	Ezrin	EZR	1.369
191	sp|Q14697|GANAB_HUMAN	Neutral alpha-glucosidase AB	GANAB	1.369
192	sp|P63244|RACK1_HUMAN	Receptor of activated protein C kinase 1	RACK1	1.368
193	sp|P09874|PARP1_HUMAN	Poly [ADP-ribose]	PARP1	1.368

		polymerase 1		
194	sp|P05091|ALDH2_HUMAN	Aldehyde dehydrogenase, mitochondrial	ALDH2	1.367
195	sp|Q13126|MTAP_HUMAN	S-methyl-5'-thioadenosine phosphorylase	MTAP	1.367
196	sp|P06396|GELS_HUMAN	Gelsolin	GSN	1.367
197	sp|P09622|DLDH_HUMAN	Dihydrolipoyl dehydrogenase, mitochondrial	DLD	1.367
198	sp|P62993|GRB2_HUMAN	Growth factor receptor-bound protein 2	GRB2	1.366
199	sp|P07954|FUMH_HUMAN	Fumarate hydratase, mitochondrial	FH	1.366
200	sp|P27797|CALR_HUMAN	Calreticulin	CALR	1.366
201	sp|P67936|TPM4_HUMAN	Tropomyosin alpha-4 chain	TPM4	1.365
202	sp|Q9UHB6|LIMA1_HUMAN	LIM domain and actin-binding protein 1	LIMA1	1.365
203	sp|Q09666|AHNK_HUMAN	Neuroblast differentiation-associated protein AHNAK	AHNAK	1.364
204	sp|Q00688|FKBP3_HUMAN	Peptidyl-prolyl cis-trans isomerase FKBP3	FKBP3	1.363
205	sp|P0DP25|CALM3_HUMAN	Calmodulin-3	CALM3	1.362
206	sp|P13804|ETFA_HUMAN	Electron transfer flavoprotein subunit alpha, mitochondrial	ETFA	1.362
207	sp|Q13442|HAP28_HUMAN	28 kDa heat- and acid-stable phosphoprotein	PDAP1	1.361
208	sp|Q13087|PDIA2_HUMAN	Protein disulfide-isomerase A2	PDIA2	1.360
209	sp|P23246|SFPQ_HUMAN	Splicing factor, proline- and glutamine-rich	SFPQ	1.360
210	sp|P04179|SODM_HUMAN	Superoxide dismutase [Mn], mitochondrial	SOD2	1.360
211	sp|O43768|ENSA_HUMAN	Alpha-endosulfine	ENSA	1.358
212	sp|P28838|AMPL_HUMAN	Cytosol aminopeptidase	LAP3	1.357
213	sp|Q01995|TAGL_HUMAN	Transgelin	TAGLN	1.356
214	sp|Q14257|RCN2_HUMAN	Reticulocalbin-2	RCN2	1.356
215	sp|O43491|E41L2_HUMAN	Band 4.1-like protein 2	EPB41L2	1.355
216	sp|Q15075|EEA1_HUMAN	Early endosome antigen 1	EEA1	1.355
217	sp|Q12904|AIMP1_HUMAN	Aminoacyl tRNA synthase complex-interacting multifunctional protein 1	AIMP1	1.354
218	sp|P30040|ERP29_HUMAN	Endoplasmic reticulum resident protein 29	ERP29	1.354
219	sp|P32119|PRDX2_HUMAN	Peroxiredoxin-2	PRDX2	1.354
220	sp|P49748|ACADV_HUMAN	Very long-chain specific acyl-CoA dehydrogenase, mitochondrial	ACADVL	1.352
221	sp|Q9BSE5|SPEB_HUMAN	Agmatinase, mitochondrial	AGMAT	1.352

222	sp|P54577|SYYC_HUMAN	Tyrosine--tRNA ligase, cytoplasmic	YARS	1.351
223	sp|Q96I99|SUCB2_HUMAN	Succinate--CoA ligase [GDP-forming] subunit beta, mitochondrial	SUCLG2	1.350
224	sp|P30101|PDIA3_HUMAN	Protein disulfide-isomerase A3	PDIA3	1.350
225	sp|Q9UN86|G3BP2_HUMAN	Ras GTPase-activating protein-binding protein 2	G3BP2	1.349
226	sp|O43852|CALU_HUMAN	Calumenin	CALU	1.349
227	sp|P38117|ETFB_HUMAN	Electron transfer flavoprotein subunit beta	ETFB	1.348
228	sp|P63208|SKP1_HUMAN	S-phase kinase-associated protein 1	SKP1	1.348
229	sp|P07148|FABPL_HUMAN	Fatty acid-binding protein, liver	FABP1	1.347
230	sp|P61978|HNRPK_HUMAN	Heterogeneous nuclear ribonucleoprotein K	HNRNPK	1.347
231	sp|P55072|TERA_HUMAN	Transitional endoplasmic reticulum ATPase	VCP	1.345
232	sp|P14625|ENPL_HUMAN	Endoplasmin	HSP90B1	1.345
233	sp|P09525|ANXA4_HUMAN	Annexin A4	ANXA4	1.345
234	sp|P07237|PDIA1_HUMAN	Protein disulfide-isomerase	P4HB	1.344
235	sp|P13667|PDIA4_HUMAN	Protein disulfide-isomerase A4	PDIA4	1.344
236	sp|Q12849|GRSF1_HUMAN	G-rich sequence factor 1	GRSF1	1.344
237	sp|P07910|HNRPC_HUMAN	Heterogeneous nuclear ribonucleoproteins C1/C2	HNRNPC	1.344
238	sp|Q99623|PHB2_HUMAN	Prohibitin-2	PHB2	1.344
239	sp|O75367|H2AY_HUMAN	Core histone macro-H2A.1	H2AFY	1.343
240	sp|O60218|AK1BA_HUMAN	Aldo-keto reductase family 1 member B10	AKR1B10	1.342
241	sp|P30041|PRDX6_HUMAN	Peroxiredoxin-6	PRDX6	1.341
242	sp|P31948|STIP1_HUMAN	Stress-induced-phosphoprotein 1	STIP1	1.341
243	sp|Q9Y4L1|HYOU1_HUMAN	Hypoxia up-regulated protein 1	HYOU1	1.341
244	sp|P43686|PRS6B_HUMAN	26S proteasome regulatory subunit 6B	PSMC4	1.340
245	sp|Q9Y2W1|TR150_HUMAN	Thyroid hormone receptor-associated protein 3	THRAP3	1.340
246	sp|P13010|XRCC5_HUMAN	X-ray repair cross-complementing protein 5	XRCC5	1.340
247	sp|P52272|HNRPM_HUMAN	Heterogeneous nuclear ribonucleoprotein M	HNRNPM	1.339
248	sp|P63241|IF5A1_HUMAN	Eukaryotic translation initiation factor 5A-1	EIF5A	1.338
249	sp|O00764|PDXK_HUMAN	Pyridoxal kinase	PDXK	1.337
250	sp|P46783|RS10_HUMAN	40S ribosomal protein S10	RPS10	1.336

251	sp|P12956|XRCC6_HUMAN	X-ray repair cross-complementing protein 6	XRCC6	1.336
252	sp|P11940|PABP1_HUMAN	Polyadenylate-binding protein 1	PABPC1	1.335
253	sp|P20042|IF2B_HUMAN	Eukaryotic translation initiation factor 2 subunit 2	EIF2S2	1.334
254	sp|P25705|ATPA_HUMAN	ATP synthase subunit alpha, mitochondrial	ATP5F1A	1.333
255	sp|Q8TAE8|G45IP_HUMAN	Growth arrest and DNA damage-inducible proteins-interacting protein 1	GADD45GIP 1	1.332
256	sp|Q13573|SNW1_HUMAN	SNW domain-containing protein 1	SNW1	1.332
257	sp|Q15417|CNN3_HUMAN	Calponin-3	CNN3	1.332
258	sp|Q92783|STAM1_HUMAN	Signal transducing adapter molecule 1	STAM	1.331
259	sp|P20700|LMNB1_HUMAN	Lamin-B1	LMNB1	1.331
260	sp|Q9Y2B0|CNPY2_HUMAN	Protein canopy homolog 2	CNPY2	1.331
261	sp|O14745|NHRF1_HUMAN	Na(+)/H(+) exchange regulatory cofactor NHE-RF1	SLC9A3R1	1.330
262	sp|O95831|AIFM1_HUMAN	Apoptosis-inducing factor 1, mitochondrial	AIFM1	1.330
263	sp|P17980|PRS6A_HUMAN	26S proteasome regulatory subunit 6A	PSMC3	1.330
264	sp|P60174|TPIS_HUMAN	Triosephosphate isomerase	TPI1	1.329
265	sp|P23141|EST1_HUMAN	Liver carboxylesterase 1	CES1	1.329
266	sp|P46087|NOP2_HUMAN	Probable 28S rRNA (cytosine(4447)-C(5))-methyltransferase	NOP2	1.329
267	sp|P00352|AL1A1_HUMAN	Retinal dehydrogenase 1	ALDH1A1	1.328
268	sp|O60664|PLIN3_HUMAN	Perilipin-3	PLIN3	1.327
269	sp|P02545|LMNA_HUMAN	Prelamin-A/C	LMNA	1.327
270	sp|O60506|HNRPQ_HUMAN	Heterogeneous nuclear ribonucleoprotein Q	SYNCRIP	1.325
271	sp|O00566|MPP10_HUMAN	U3 small nucleolar ribonucleoprotein protein MPP10	MPHOSPH10	1.324
272	sp|P61247|RS3A_HUMAN	40S ribosomal protein S3a	RPS3A	1.324
273	sp|P52565|GDIR1_HUMAN	Rho GDP-dissociation inhibitor 1	ARHGDIA	1.324
274	sp|P07737|PROF1_HUMAN	Profilin-1	PFN1	1.323
275	sp|O43615|TIM44_HUMAN	Mitochondrial import inner membrane translocase subunit TIM44	TIMM44	1.321
276	sp|Q08426|ECHP_HUMAN	Peroxisomal bifunctional enzyme	EHHADH	1.320
277	sp|P21964|COMT_HUMAN	Catechol O-methyltransferase	COMT	1.320
278	sp|P00367|DHE3_HUMAN	Glutamate dehydrogenase 1,	GLUD1	1.319

		mitochondrial		
279	sp|P24539|AT5F1_HUMAN	ATP synthase F(0) complex subunit B1, mitochondrial	ATP5F1	1.317
280	sp|P46108|CRK_HUMAN	Adapter molecule crk	CRK	1.316
281	sp|Q92841|DDX17_HUMAN	Probable ATP-dependent RNA helicase DDX17	DDX17	1.311
282	sp|P11021|BIP_HUMAN	Endoplasmic reticulum chaperone BiP	HSPA5	1.310
283	sp|P26368|U2AF2_HUMAN	Splicing factor U2AF 65 kDa subunit	U2AF2	1.310
284	sp|Q15293|RCN1_HUMAN	Reticulocalbin-1	RCN1	1.310
285	sp|P61088|UBE2N_HUMAN	Ubiquitin-conjugating enzyme E2 N	UBE2N	1.309
286	sp|Q15691|MARE1_HUMAN	Microtubule-associated protein RP/EB family member 1	MAPRE1	1.307
287	sp|Q9BSH4|TACO1_HUMAN	Translational activator of cytochrome c oxidase 1	TACO1	1.306
288	sp|O75821|EIF3G_HUMAN	Eukaryotic translation initiation factor 3 subunit G	EIF3G	1.305
289	sp|Q9BZE9|ASPC1_HUMAN	Tether containing UBX domain for GLUT4	ASPSCR1	1.302
290	sp|Q9NR30|DDX21_HUMAN	Nucleolar RNA helicase 2	DDX21	0.710
291	sp|P11047|LAMC1_HUMAN	Laminin subunit gamma-1	LAMC1	0.710
292	sp|P07900|HS90A_HUMAN	Heat shock protein HSP 90-alpha	HSP90AA1	0.709
293	sp|Q9P2E9|RRBP1_HUMAN	Ribosome-binding protein 1	RRBP1	0.709
294	sp|P55884|EIF3B_HUMAN	Eukaryotic translation initiation factor 3 subunit B	EIF3B	0.709
295	sp|P60842|IF4A1_HUMAN	Eukaryotic initiation factor 4A-I	EIF4A1	0.709
296	sp|Q08211|DHX9_HUMAN	ATP-dependent RNA helicase A	DHX9	0.709
297	sp|Q14677|EPN4_HUMAN	Clathrin interactor 1	CLINT1	0.709
298	sp|Q6Y7W6|GGYF2_HUMAN	GRB10-interacting GYF protein 2	GIGYF2	0.708
299	sp|Q16851|UGPA_HUMAN	UTP--glucose-1-phosphate uridylyltransferase	UGP2	0.708
300	sp|Q9UBQ7|GRHPR_HUMAN	Glyoxylate reductase/hydroxypyruvate reductase	GRHPR	0.708
301	sp|Q04637|IF4G1_HUMAN	Eukaryotic translation initiation factor 4 gamma 1	EIF4G1	0.708
302	sp|P50552|VASP_HUMAN	Vasodilator-stimulated phosphoprotein	VASP	0.707
303	sp|Q9Y295|DRG1_HUMAN	Developmentally-regulated GTP-binding protein 1	DRG1	0.707
304	sp|P50990|TCPQ_HUMAN	T-complex protein 1 subunit theta	CCT8	0.707

305	sp|Q92616|GCN1_HUMAN	eIF-2-alpha kinase activator GCN1	GCN1	0.707
306	sp|P25205|MCM3_HUMAN	DNA replication licensing factor MCM3	MCM3	0.707
307	sp|P24666|PPAC_HUMAN	Low molecular weight phosphotyrosine protein phosphatase	ACP1	0.706
308	sp|P02751|FINC_HUMAN	Fibronectin	FN1	0.706
309	sp|P54578|UBP14_HUMAN	Ubiquitin carboxyl-terminal hydrolase 14	USP14	0.706
310	sp|P40939|ECHA_HUMAN	Trifunctional enzyme subunit alpha, mitochondrial	HADHA	0.705
311	sp|P49915|GUAA_HUMAN	GMP synthase [glutamine-hydrolyzing]	GMPS	0.704
312	sp|O60763|USO1_HUMAN	General vesicular transport factor p115	USO1	0.704
313	sp|P46939|UTRO_HUMAN	Utrophin	UTRN	0.703
314	sp|Q15067|ACOX1_HUMAN	Peroxisomal acyl-coenzyme A oxidase 1	ACOX1	0.703
315	sp|P00338|LDHA_HUMAN	L-lactate dehydrogenase A chain	LDHA	0.703
316	sp|O75396|SC22B_HUMAN	Vesicle-trafficking protein SEC22b	SEC22B	0.702
317	sp|O43252|PAPS1_HUMAN	Bifunctional 3'-phosphoadenosine 5'-phosphosulfate synthase 1	PAPSS1	0.702
318	sp|Q9Y617|SERC_HUMAN	Phosphoserine aminotransferase	PSAT1	0.701
319	sp|P42704|LPPRC_HUMAN	Leucine-rich PPR motif-containing protein, mitochondrial	LRPPRC	0.701
320	sp|P12081|SYHC_HUMAN	Histidine--tRNA ligase, cytoplasmic	HARS	0.701
321	sp|P49736|MCM2_HUMAN	DNA replication licensing factor MCM2	MCM2	0.701
322	sp|P50895|BCAM_HUMAN	Basal cell adhesion molecule	BCAM	0.701
323	sp|P26641|EF1G_HUMAN	Elongation factor 1-gamma	EEF1G	0.700
324	sp|P58107|EPIPL_HUMAN	Epiplakin	EPPK1	0.699
325	sp|Q96AG4|LRC59_HUMAN	Leucine-rich repeat-containing protein 59	LRRC59	0.699
326	sp|Q01082|SPTB2_HUMAN	Spectrin beta chain, non-erythrocytic 1	SPTBN1	0.699
327	sp|Q00610|CLH1_HUMAN	Clathrin heavy chain 1	CLTC	0.699
328	sp|P33992|MCM5_HUMAN	DNA replication licensing factor MCM5	MCM5	0.699
329	sp|P61221|ABCE1_HUMAN	ATP-binding cassette sub-family E member 1	ABCE1	0.699
330	sp|O76003|GLRX3_HUMAN	Glutaredoxin-3	GLRX3	0.699
331	sp|P23526|SAHH_HUMAN	Adenosylhomocysteinase	AHCY	0.699

332	sp|Q9UJS0|CMC2_HUMAN	Calcium-binding mitochondrial carrier protein Aralar2	SLC25A13	0.699
333	sp|P54886|P5CS_HUMAN	Delta-1-pyrroline-5-carboxylate synthase	ALDH18A1	0.698
334	sp|Q12906|ILF3_HUMAN	Interleukin enhancer-binding factor 3	ILF3	0.698
335	sp|P04406|G3P_HUMAN	Glyceraldehyde-3-phosphate dehydrogenase	GAPDH	0.698
336	sp|P26640|SYVC_HUMAN	Valine--tRNA ligase	VARS	0.697
337	sp|P78527|PRKDC_HUMAN	DNA-dependent protein kinase catalytic subunit	PRKDC	0.697
338	sp|P11586|C1TC_HUMAN	C-1-tetrahydrofolate synthase, cytoplasmic	MTHFD1	0.697
339	sp|Q93009|UBP7_HUMAN	Ubiquitin carboxyl-terminal hydrolase 7	USP7	0.697
340	sp|Q14694|UBP10_HUMAN	Ubiquitin carboxyl-terminal hydrolase 10	USP10	0.697
341	sp|P55157|MTP_HUMAN	Microsomal triglyceride transfer protein large subunit	MTTP	0.696
342	sp|P22695|QCR2_HUMAN	Cytochrome b-c1 complex subunit 2, mitochondrial	UQCRC2	0.696
343	sp|Q13200|PSMD2_HUMAN	26S proteasome non-ATPase regulatory subunit 2	PSMD2	0.696
344	sp|P08559|ODPA_HUMAN	Pyruvate dehydrogenase E1 component subunit alpha, somatic form, mitochondrial	PDHA1	0.695
345	sp|P14618|KPYM_HUMAN	Pyruvate kinase PKM	PKM	0.695
346	sp|O43707|ACTN4_HUMAN	Alpha-actinin-4	ACTN4	0.695
347	sp|P41252|SYIC_HUMAN	Isoleucine--tRNA ligase, cytoplasmic	IARS	0.695
348	sp|P60228|EIF3E_HUMAN	Eukaryotic translation initiation factor 3 subunit E	EIF3E	0.695
349	sp|Q99613|EIF3C_HUMAN	Eukaryotic translation initiation factor 3 subunit C	EIF3C	0.695
350	sp|O43175|SERA_HUMAN	D-3-phosphoglycerate dehydrogenase	PHGDH	0.695
351	sp|O95394|AGM1_HUMAN	Phosphoacetylglucosamine mutase	PGM3	0.694
352	sp|P49368|TCPG_HUMAN	T-complex protein 1 subunit gamma	CCT3	0.694
353	sp|P31040|SDHA_HUMAN	Succinate dehydrogenase [ubiquinone] flavoprotein subunit, mitochondrial	SDHA	0.693
354	sp|P40925|MDHC_HUMAN	Malate dehydrogenase, cytoplasmic	MDH1	0.693
355	sp|Q13085|ACACA_HUMAN	Acetyl-CoA carboxylase 1	ACACA	0.693
356	sp|O94979|SC31A_HUMAN	Protein transport protein Sec31A	SEC31A	0.693

357	sp|P11498|PYC_HUMAN	Pyruvate carboxylase, mitochondrial	PC	0.693
358	sp|P30153|2AAA_HUMAN	Serine/threonine-protein phosphatase 2A 65 kDa regulatory subunit A alpha isoform	PPP2R1A	0.693
359	sp|P46109|CRKL_HUMAN	Crk-like protein	CRKL	0.693
360	sp|Q00341|VIGLN_HUMAN	Vigilin	HDLBP	0.691
361	sp|P14550|AK1A1_HUMAN	Alcohol dehydrogenase [NADP(+)]	AKR1A1	0.691
362	sp|P51570|GALK1_HUMAN	Galactokinase	GALK1	0.690
363	sp|P09543|CN37_HUMAN	2',3'-cyclic-nucleotide 3'-phosphodiesterase	CNP	0.690
364	sp|P06744|G6PI_HUMAN	Glucose-6-phosphate isomerase	GPI	0.690
365	sp|O75116|ROCK2_HUMAN	Rho-associated protein kinase 2	ROCK2	0.689
366	sp|O95336|6PGL_HUMAN	6-phosphogluconolactonase	PGLS	0.689
367	sp|P00387|NB5R3_HUMAN	NADH-cytochrome b5 reductase 3	CYB5R3	0.689
368	sp|P37268|FDFT_HUMAN	Squalene synthase	FDFT1	0.689
369	sp|P50395|GDIB_HUMAN	Rab GDP dissociation inhibitor beta	GDI2	0.689
370	sp|Q16555|DPYL2_HUMAN	Dihydropyrimidinase-related protein 2	DPYSL2	0.689
371	sp|O94906|PRP6_HUMAN	Pre-mRNA-processing factor 6	PRPF6	0.688
372	sp|P04843|RPN1_HUMAN	Dolichyl-diphosphooligosaccharide--protein glycosyltransferase subunit 1	RPN1	0.688
373	sp|P06733|ENOA_HUMAN	Alpha-enolase	ENO1	0.688
374	sp|P53396|ACLY_HUMAN	ATP-citrate synthase	ACLY	0.687
375	sp|Q8N392|RHG18_HUMAN	Rho GTPase-activating protein 18	ARHGAP18	0.686
376	sp|O00571|DDX3X_HUMAN	ATP-dependent RNA helicase DDX3X	DDX3X	0.686
377	sp|Q02818|NUCB1_HUMAN	Nucleobindin-1	NUCB1	0.686
378	sp|Q13724|MOGS_HUMAN	Mannosyl-oligosaccharide glucosidase	MOGS	0.685
379	sp|Q7Z4W1|DCXR_HUMAN	L-xylulose reductase	DCXR	0.685
380	sp|Q13813|SPTN1_HUMAN	Spectrin alpha chain, non-erythrocytic 1	SPTAN1	0.685
381	sp|Q9BT78|CSN4_HUMAN	COP9 signalosome complex subunit 4	COPS4	0.685
382	sp|O75083|WDR1_HUMAN	WD repeat-containing protein 1	WDR1	0.684
383	sp|P55786|PSA_HUMAN	Puromycin-sensitive aminopeptidase	NPEPPS	0.684

384	sp|P54136|SYRC_HUMAN	Arginine--tRNA ligase, cytoplasmic	RARS	0.684
385	sp|P53621|COPA_HUMAN	Coatomer subunit alpha	COPA	0.683
386	sp|P00966|ASSY_HUMAN	Argininosuccinate synthase	ASS1	0.683
387	sp|P14868|SYDC_HUMAN	Aspartate--tRNA ligase, cytoplasmic	DARS	0.683
388	sp|P00558|PGK1_HUMAN	Phosphoglycerate kinase 1	PGK1	0.683
389	sp|O75643|U520_HUMAN	U5 small nuclear ribonucleoprotein 200 kDa helicase	SNRNP200	0.683
390	sp|O00410|IPO5_HUMAN	Importin-5	IPO5	0.683
391	sp|O15305|PMM2_HUMAN	Phosphomannomutase 2	PMM2	0.682
392	sp|P21333|FLNA_HUMAN	Filamin-A	FLNA	0.682
393	sp|O00151|PDLI1_HUMAN	PDZ and LIM domain protein 1	PDLIM1	0.681
394	sp|Q8IY81|SPB1_HUMAN	pre-rRNA processing protein FTSJ3	FTSJ3	0.681
395	sp|P62244|RS15A_HUMAN	40S ribosomal protein S15a	RPS15A	0.680
396	sp|Q7KZF4|SND1_HUMAN	Staphylococcal nuclease domain-containing protein 1	SND1	0.680
397	sp|O00429|DNM1L_HUMAN	Dynamin-1-like protein	DNM1L	0.680
398	sp|Q86UE4|LYRIC_HUMAN	Protein LYRIC	MTDH	0.679
399	sp|P47756|CAPZB_HUMAN	F-actin-capping protein subunit beta	CAPZB	0.679
400	sp|P49419|AL7A1_HUMAN	Alpha-aminoadipic semialdehyde dehydrogenase	ALDH7A1	0.679
401	sp|P11413|G6PD_HUMAN	Glucose-6-phosphate 1-dehydrogenase	G6PD	0.679
402	sp|Q8WWM7|ATX2L_HUMAN	Ataxin-2-like protein	ATXN2L	0.678
403	sp|P0DMV9|HS71B_HUMAN	Heat shock 70 kDa protein 1B	HSPA1B	0.678
404	sp|Q9Y265|RUVB1_HUMAN	RuvB-like 1	RUVBL1	0.678
405	sp|P01023|A2MG_HUMAN	Alpha-2-macroglobulin	A2M	0.678
406	sp|O75369|FLNB_HUMAN	Filamin-B	FLNB	0.678
407	sp|Q14974|IMB1_HUMAN	Importin subunit beta-1	KPNB1	0.678
408	sp|P18124|RL7_HUMAN	60S ribosomal protein L7	RPL7	0.678
409	sp|Q8NE71|ABCF1_HUMAN	ATP-binding cassette sub-family F member 1	ABCF1	0.677
410	sp|P50991|TCPD_HUMAN	T-complex protein 1 subunit delta	CCT4	0.677
411	sp|Q14247|SRC8_HUMAN	Src substrate cortactin	CTTN	0.677
412	sp|Q15393|SF3B3_HUMAN	Splicing factor 3B subunit 3	SF3B3	0.676
413	sp|P15924|DESP_HUMAN	Desmoplakin	DSP	0.676
414	sp|P09327|VILI_HUMAN	Villin-1	VIL1	0.675
415	sp|P33176|KINH_HUMAN	Kinesin-1 heavy chain	KIF5B	0.675
416	sp|Q6P996|PDXD1_HUMAN	Pyridoxal-dependent	PDXDC1	0.674

		decarboxylase domain-containing protein 1		
417	sp|Q9NTK5|OLA1_HUMAN	Obg-like ATPase 1	OLA1	0.674
418	sp|P16435|NCPR_HUMAN	NADPH--cytochrome P450 reductase	POR	0.674
419	sp|Q9BWD1|THIC_HUMAN	Acetyl-CoA acetyltransferase, cytosolic	ACAT2	0.672
420	sp|P52209|6PGD_HUMAN	6-phosphogluconate dehydrogenase, decarboxylating	PGD	0.672
421	sp|P13639|EF2_HUMAN	Elongation factor 2	EEF2	0.671
422	sp|P49588|SYAC_HUMAN	Alanine--tRNA ligase, cytoplasmic	AARS	0.671
423	sp|O60716|CTND1_HUMAN	Catenin delta-1	CTNND1	0.671
424	sp|P16152|CBR1_HUMAN	Carbonyl reductase [NADPH] 1	CBR1	0.670
425	sp|O75153|CLU_HUMAN	Clustered mitochondria protein homolog	CLUH	0.670
426	sp|P61158|ARP3_HUMAN	Actin-related protein 3	ACTR3	0.670
427	sp|P22234|PUR6_HUMAN	Multifunctional protein ADE2	PAICS	0.670
428	sp|Q9Y490|TLN1_HUMAN	Talin-1	TLN1	0.669
429	sp|P34932|HSP74_HUMAN	Heat shock 70 kDa protein 4	HSPA4	0.668
430	sp|P39748|FEN1_HUMAN	Flap endonuclease 1	FEN1	0.666
431	sp|P33993|MCM7_HUMAN	DNA replication licensing factor MCM7	MCM7	0.666
432	sp|P12814|ACTN1_HUMAN	Alpha-actinin-1	ACTN1	0.665
433	sp|Q14566|MCM6_HUMAN	DNA replication licensing factor MCM6	MCM6	0.665
434	sp|P07437|TBB5_HUMAN	Tubulin beta chain	TUBB	0.665
435	sp|P55263|ADK_HUMAN	Adenosine kinase	ADK	0.665
436	sp|P15170|ERF3A_HUMAN	Eukaryotic peptide chain release factor GTP-binding subunit ERF3A	GSPT1	0.663
437	sp|P49257|LMAN1_HUMAN	Protein ERGIC-53	LMAN1	0.662
438	sp|Q9UHD8|SEPT9_HUMAN	Septin-9	Sep-09	0.662
439	sp|Q01581|HMCS1_HUMAN	Hydroxymethylglutaryl-CoA synthase, cytoplasmic	HMGCS1	0.662
440	sp|P38606|VATA_HUMAN	V-type proton ATPase catalytic subunit A	ATP6V1A	0.662
441	sp|O43776|SYNC_HUMAN	Asparagine--tRNA ligase, cytoplasmic	NARS	0.662
442	sp|P35580|MYH10_HUMAN	Myosin-10	MYH10	0.660
443	sp|P04844|RPN2_HUMAN	Dolichyl-diphosphooligosaccharide--protein glycosyltransferase subunit 2	RPN2	0.659
444	sp|Q00839|HNRPU_HUMAN	Heterogeneous nuclear ribonucleoprotein U	HNRNPU	0.657
445	sp|P62851|RS25_HUMAN	40S ribosomal protein S25	RPS25	0.657

446	sp|P30740|ILEU_HUMAN	Leukocyte elastase inhibitor	SERPINB1	0.656
447	sp|P02647|APOA1_HUMAN	Apolipoprotein A-I	APOA1	0.655
448	sp|P08238|HS90B_HUMAN	Heat shock protein HSP 90-beta	HSP90AB1	0.654
449	sp|P07814|SYEP_HUMAN	Bifunctional glutamate/proline--tRNA ligase	EPRS	0.654
450	sp|P39656|OST48_HUMAN	Dolichyl-diphosphooligosaccharide--protein glycosyltransferase 48 kDa subunit	DDOST	0.650
451	sp|Q9NYU2|UGGG1_HUMAN	UDP-glucose:glycoprotein glucosyltransferase 1	UGGT1	0.650
452	sp|Q9HC38|GLOD4_HUMAN	Glyoxalase domain-containing protein 4	GLOD4	0.650
453	sp|Q99536|VAT1_HUMAN	Synaptic vesicle membrane protein VAT-1 homolog	VAT1	0.650
454	sp|P35998|PRS7_HUMAN	26S proteasome regulatory subunit 7	PSMC2	0.647
455	sp|P31350|RIR2_HUMAN	Ribonucleoside-diphosphate reductase subunit M2	RRM2	0.646
456	sp|Q8WUM4|PDC6I_HUMAN	Programmed cell death 6-interacting protein	PDCD6IP	0.645
457	sp|Q96QK1|VPS35_HUMAN	Vacuolar protein sorting-associated protein 35	VPS35	0.644
458	sp|P62191|PRS4_HUMAN	26S proteasome regulatory subunit 4	PSMC1	0.642
459	sp|P01009|A1AT_HUMAN	Alpha-1-antitrypsin	SERPINA1	0.641
460	sp|P07339|CATD_HUMAN	Cathepsin D	CTSD	0.640
461	sp|P62760|VISL1_HUMAN	Visinin-like protein 1	VSNL1	0.639
462	sp|P49327|FAS_HUMAN	Fatty acid synthase	FASN	0.637
463	sp|P25786|PSA1_HUMAN	Proteasome subunit alpha type-1	PSMA1	0.636
464	sp|P35579|MYH9_HUMAN	Myosin-9	MYH9	0.635
465	sp|Q9Y6M1|IF2B2_HUMAN	Insulin-like growth factor 2 mRNA-binding protein 2	IGF2BP2	0.635
466	sp|P52292|IMA1_HUMAN	Importin subunit alpha-1	KPNA2	0.629
467	sp|P48047|ATPO_HUMAN	ATP synthase subunit O, mitochondrial	ATP5O	0.628
468	sp|Q14152|EIF3A_HUMAN	Eukaryotic translation initiation factor 3 subunit A	EIF3A	0.625
469	sp|P51812|KS6A3_HUMAN	Ribosomal protein S6 kinase alpha-3	RPS6KA3	0.622
470	sp|Q9UNX3|RL26L_HUMAN	60S ribosomal protein L26-like 1	RPL26L1	0.618
471	sp|P61313|RL15_HUMAN	60S ribosomal protein L15	RPL15	0.618
472	sp|Q14204|DYHC1_HUMAN	Cytoplasmic dynein 1 heavy chain 1	DYNC1H1	0.612
473	sp|P02656|APOC3_HUMAN	Apolipoprotein C-III	APOC3	0.606

474	sp|Q9UQ35|SRRM2_HUMAN	Serine/arginine repetitive matrix protein 2	SRRM2	0.595
