# Supplementary material for: Proteomic analysis reveals USP7 as a novel regulator of palmitic acid-induced hepatocellular carcinoma cell death
Source: Cell Death Dis. 2022 Jun 22;13(6):563. doi: 10.1038/s41419-022-05003-4 (PMC9217975; doi:10.1038/s41419-022-05003-4)
Supplement: Supplementary file 11 — Supplementary Table 2 [file 41419_2022_5003_MOESM11_ESM.pdf]

Term description	Observed gene count	FDR	Matching proteins (up regulated)	Matching proteins (down regulated)	Biological Progress noRedundant (FDR≤0.05)
Ubiquitin-dependent protein catabolic process	21	0.0333	FBXO2,HSP90B1, HSPA5,NSFL1C,PCNP,PSMB5,PSMC3,PSMC4,RAD23B,RPS27A,SKP1,SQSTM1,UBQLN1, VCP	USP10,USP14, USP7, PSMD2, PSMC2, PSMC1, PSMA1	Positive regulation of catabolic process<br>Positive regulation of proteolysis<br>Protein-DNA complex subunit organization<br>Response to ER stress<br>Protein folding<br>Regulation of protein catabolic process<br>Post-translational protein modification<br>Proteasomal protein catabolic process<br>Protein modification by small protein removal<br>Post-replication repair
Cell cycle	52	0.0017	SON, NPM1, RRS1, STMN1, PCNP, NUP62, YWHAE, NSFL1C, AKAP8, TPR, PA2G4, CDC5L, MAP4, SFN, NASP, EZR, ENSA, EPB41L2, SKP1, PHB2, GADD45GIP1, LMNA, MAPRE1	HSP90AA1, GIGYF2, MCM3, MCM2, SPTBN1, CLTC, PRKDC, PHGDH, PPP2R1A, ROCK2, FLNA, RUVBL1, KPNB1, ACTR3, FEN1, MCM7, MCM6, TUBB, GSPT1, MYH10, RRM2, HNRNPU, PDCD6IP, MYH9, RPS6KA3, DYNC1H1	Establishment of organelle localization<br>Regulation of cytoskeleton organization<br>Regulation of cell cycle phase transition<br>Chromosome segregation<br>Mitotic cell cycle phase transition<br>Cell cycle<br>G1/S phase transition<br>Cell cycle G2/M phase transition<br>Regulation of microtubule-based process<br>Microtubule cytoskeleton organization involved in mitosis<br>Spindle organization
Regulation of cell death	86	6.03E-10	SQSTM1, SON, FGA, NPM1, CYCS, CAST, RPS27A, HMGB1, ICAM1, NUP62, APOE, YWHAE, AKR1C3, YBX3, UBQLN1, RSL1D1, PRDX5, DHRS2, PHB, HSPA9, DNAJA3, PYCR1, HSPE1, HMGB2, SERBP1, YWHAZ, PARK7, ACIN1, CAT,	EIF4G1, LDHA, GAPDH, PRKDC, ACTN4, PPP2R1A, ENO1, DDX3X, FLNA, DNM1L, MTDH, G6PD, HSPA1B, CTTN, VIL1, POR, AARS, ACTN1, HSP90AB1, VPS35, PSMC1,	Response to toxic substance<br>Regulation of peptidase activity<br>Response to oxygen levels<br>Reactive oxygen species metabolic process<br>Response to endoplasmic reticulum stress<br>Response to oxidative stress<br>Neuron death<br>Protein folding<br>Intrinsic apoptotic signaling pathway<br>Regulation of apoptotic signaling pathway

			NQO1, ACAA2, PA2G4, SET, C1QBP, ASNS, TXNDC5, SFN, HSPD1, NONO, PRDX3, PARP1, GSN, CALR, PDIA2, SFPQ, SOD2, ERP29, PRDX2, PDIA3, FABP1, HNRNPK, VCP, HSP90B1, ANXA4, P4HB, PHB2, HYOU1, SLC9A3R1, AIFM1, LMNA, RPS3A, ARHGDIA, HSPA5	RPS6KA3	
