# Supplementary material for: Proteomic analysis reveals USP7 as a novel regulator of palmitic acid-induced hepatocellular carcinoma cell death
Source: Cell Death Dis. 2022 Jun 22;13(6):563. doi: 10.1038/s41419-022-05003-4 (PMC9217975; doi:10.1038/s41419-022-05003-4)
Supplement: Supplementary file 22 — Manuscript_CDD_Merged [file 41419_2022_5003_MOESM22_ESM.docx]

**Proteomic analysis revealed USP7 as a novel regulator of palmitic acid-induced hepatocellular carcinoma cell death**

Sandhini Saha^1^, Rohit Verma^2^, Chandan Kumar^3^, Bhoj Kumar^1^, Amit Kumar Dey^1^, Milan Surjit^2^, Sivaram V S Mylavarapu^3^ and Tushar Kanti Maiti^1*^

^1^Functional Proteomics Laboratory, Regional Centre for Biotechnology, NCR Biotech Science Cluster, Faridabad, 121001, India.

^2^Virology Laboratory, Translational Health Science and Technology Institute, NCR Biotech Science Cluster, Faridabad, Haryana, India

^3^Laboratory of Cellular Dynamics, Regional Centre for Biotechnology, NCR Biotech Science Cluster, Faridabad, 121001, India.

^*^To whom correspondence should be addressed. E-mail: [tkmaiti@rcb.res.in](mailto:tkmaiti@rcb.res.in)

**Running title:** USP7 mediates cellular death in lipotoxic conditions.

**Abstract**

Nutrient surplus and a consequent free fatty acid accumulation in the liver causes hepatosteatosis. The exposure of free fatty acids to cultured hepatocyte and hepatocellular carcinoma cell lines induces cellular stress, organelle adaptation and subsequent cell death. Despite many studies, the mechanism associated with lipotoxicity and subsequent cell death still remains poorly understood. Here, we have used proteomics approach to circumvent the mechanism for lipotoxicity using hepatocellular carcinoma cells as a model. Our quantitative proteomics data revealed that ectopic lipids accumulation in cells severely affects the ubiquitin proteasomal system. The palmitic acid (PA) partially lowered the expression of deubiquitinating enzymes USP7 which subsequently destabilize p53 and promote mitotic entry of cells. Our global phospho-proteomics analysis also provides a strong evidence of an altered cell cycle checkpoint proteins' expression that abrogates early G2/M checkpoints recovery with damaged DNA and induced mitotic catastrophe leading to hepatocyte death. We observe that palmitic acid prefers apoptosis-inducing factor (AIF) mediated cell death by depolarizing mitochondria and translocating AIF to the nucleus. In summary, the present study provides evidence of PA-induced hepatocellular death to mediate by deubiquitinase USP7 down-regulation and subsequent mitotic catastrophe.

**Keywords:** Lipotoxicity, HepG2, palmitic acid, ubiquitin proteasomal system, USP7, p53, AIFM1, NPM1, mitotic catastrophe.

**Introduction**

Prolong exposure of lipid molecules to cells alters the metabolism and cellular signaling which contributes to adverse global responses known as lipotoxicity(1–3). A high level of circulating non-esterified fatty acids causes lipotoxicity which is the signature of many metabolic disorders, including type 2 diabetes mellitus(4), cardiovascular disease(5), dyslipidemia (6), hypertension(7), atherosclerosis(8) and hepatic steatosis(9). In lipid-mediated hepatic dysfunction, hepatocytes overwhelm by multiple cytotoxic lipids, incredibly saturated fatty acids (SFAs) and show severe hepatic lipotoxicity (10,11). The hepatocyte dysfunction starts from simple steatosis to deteriorated hepatocyte function and finally reaches to extreme lipotoxicity that contributes to hepatocyte death(12). Saturated fatty acid, like palmitic acid (PA), has been shown to alter the cellular membrane fluidity and forms lipid droplets within the cells(13–15). PA is a common fatty acid (FA) accounting for 20-30% of total FA in the human body(16). PA can be provided through diet or generated via *de novo* lipogenesis(17). PA also forms lipid derivatives and protein modifications that drive cellular signalling(18,19). Cells exert excess lipid burden through the activation of intracellular metabolic pathways that promote β-oxidation(20). Thus, PA may attribute beneficial to detrimental outcomes depending upon dose and time of exposure(21–23). Increased concentration(24) and prolonged exposure of PA promote hepatotoxicity, accompanied by various factors including maladaptation of metabolic storm(25), toxic lipid accumulation(26), reactive oxygen species generation(27,28), Ca^2+^ channel dysfunction(29), ER stress(30–32), mitochondrial dysfunction(20,21), deregulated autophagy(33) and abrogated activation of several programmed to non-programmed cell death(34–36). During the last two decades, many attempts have been made to exploit omics tools including proteome, transcriptome, metabolome, and lipidome to disclose the PA-mediated key processes that dictate ultimate cell fate(37–40). Moreover, comprehensive murine models have also been explored for hepatic complications during PA toxicity(41–44). But consistent limitations due to system complexity, dissection of PA specific causal relationship with fundamental death execution is still a big challenge.

Hepatocellular carcinoma (HepG2) cells treated with PA recapitulate the global lipotoxic responses including insulin resistance to hepatocyte death(45). Therefore, using this cell-based model, we perform a comprehensive, differential quantitative proteomics study to find the regulation of the cellular process. Bioinformatics analysis of differentially expressed proteins highlights the regulation of metabolism, organelle dysfunction, and disruption of protein quality control machinery, including ubiquitin proteasomal system (UPS), autophagy and ERAD pathways. Here we identify a downregulation of deubiquitinating enzymes USP7, USP10 and USP14 upon PA exposure to cells. The partial lowering of UPS7 shows destabilization of p53 and promotes cell death. The USP7-p53-Mdm2 axis promotes caspase-independent death over caspase-dependent though activation of AIFM1 cleavage and translocation of cleaved AIF into the nucleus. The phosphoproteome and AIFM1 interactome studies identify Nucleophosmin-1 (NPM1) as a critical contributor that alters mitochondrial membrane potential and facilitates AIF-mediated cell death. Our analyses introduce mitotic catastrophe as a new roadway for hepato-lipotoxicity and subsequent hepatocyte death.

**Results**

**Differential proteomics reveals altered protein quality control upon Palmitic acid treatment to HepG2 cells**

To understand the mechanistic insight into the lipid-induced hepatocyte death, we approached a global proteomics strategy where cellular responses against palmitic acid (PA) have been studied using iTRAQ-based differential proteomics. Based on previous literature, we chose the human hepatocellular carcinoma cell line (HepG2) and palmitic acid as lipotoxic agents. We used 0.5 mM PA with 18 h exposure time based on our MTT, oil red O assay, and annexin V-FITC (Supplementary Fig. S1A-D). Next, we set out a global proteome experiment using four-plex iTRAQ reagents where HepG2 cells were exposed to 0.5 mM PA and BSA for 18 h and considered as "treated" and "control," respectively (Fig. 1A). A total of 3139 and 2702 proteins were identified in two biological replicates B1 and B2 respectively with 1 % FDR (Supplementary Fig. S2B). We captured 620 quantifiable proteins in two biological replicates (Fig. 1B) and among them, 289 proteins were upregulated and 185 proteins were downregulated on PA treatment (Supplementary Data.1, Supplementary Fig. S2C). The expression pattern of regulated proteins was visualized in the volcano plot (Fig. 1C, Supplementary Table. 1).

Next, the Over-Representation Analysis (ORA) was performed with differentially expressed proteins (DEPs) using the KEGG pathway database (Supplementary Data. 4, Fig. 1D). They are predominantly involved in metabolic pathways including TCA cycle (~48%), glycolysis/gluconeogenesis (~24%), pyruvate (~38%) and fatty acid (~24%) metabolism (Fig. 1D). Apart from metabolic processes, the protein processing in the endoplasmic reticulum (~17%) was most over-represented. The proteins associated with spliceosome (~10%) and proteasome (~16%) also enlisted within the top 12 KEGG pathways. Additionally, we also examined the top hits biological process using WEB-based GEne SeT AnaLysis Toolkit(46). The gene ontology enrichment analysis (Supplementary Data. 3) of GOBPs revealed mRNA splicing, nuclear-cytoplasmic transportation, ROS mediated process, regulation of apoptotic signaling, and post-transcriptional regulation of gene expression were enriched with upregulated proteins with positive correlation. In contrast, negative regulation of proteolysis, supramolecular fiber localization and cytoskeleton organization, negative regulation of hydrolase activity and mitotic cell cycle phase transition (Supplementary Fig. S2D) were also shown enriched with negative correlation with the down-regulated proteins. Interestingly, GOCCs analysis enriched with protein quality control machinery including chaperon complex, COP9 signalosome, spliceosome complex, ER, and peptidase complex (Supplementary Fig. S2E). In continuation, the analysis of GOMF was augmented with mRNA binding, ubiquitin-like protein ligase binding, and unfolded protein binding (Supplementary Fig. S2F). PA toxicity was accompanied by the metabolic complexity channelized through a global catabolic-anabolic network(47–49). It modulates a broad range of genes that are associated with carbohydrates, organic substances, small molecules, proteins, and nucleobase-containing compounds (Supplementary Data. 2, Supplementary Fig. S3A). Reactome pathway analysis of DEPs identified two highly dense networks. The altered translation machinery due to the metabolism of amino acids and cellular response to chemical stress connect the node to apoptosis or programmed cell death. Simultaneously, PA also impaired cellular signaling (Supplementary Data. 5), including Rho-GTPase cycle, cytosolic Ca^2+^, calnexin/calreticulin cycle. Surprisingly, PA toxicity severely hampered the cell fate (Fig. 1E). The second most highly dense network enriched with cell cycle-associated candidates is strongly connected with adjacent nodes of programmed cell death or apoptosis. Although PA toxicity drove organelle dysfunction and our proteomics data evidently highlighted mitochondrial biogenesis, ER stress, and UPR. Our proteomics analysis also emphasizes that PA toxicity is due to lipid stress and unfolded proteins stress, which directly or indirectly alters protein quality control machinery.

**Palmitic acid deregulates checkpoint-associated DUBs in HepG2 cells**

The network analysis of DEPs showed a strong connectivity of regulation of cell death, cell cycle, and ubiquitin-dependent catabolic networks. We represented these three biological processes and their associated deregulated candidates within one frame (Fig. 2A). A few candidate proteins were found to share two or more different biological processes. The ubiquitin-mediated catabolic process shared a network of both cell cycle and cell death pathways. The ubiquitin-dependent protein catabolic process was associated with the proteins with upregulation and downregulation and they were involved in protein folding, stability and ubiquitin proteasomal system (Supplementary Table. 2, Fig. 2A). On the other hand, the cell death node was associated with a large number of upregulated candidates. The cell cycle regulation due to PA exposure was majorly affected due to the down regulation of cell cycle-associated proteins and these proteins were associated with the mitosis phase transition, spindle organization, organelle distribution, and cytokinesis (Supplementary Table. 2, Fig. 2A). Similarly, the cell death execution node was highlighted with upregulated pro-apoptotic proteins and down-regulated anti-apoptotic proteins (Supplementary Table 2, Figure 2A). The closer view of annotated nodes revealed that each node consecutively shared with a large sub-node with a significant BP enrichment ratio. More precisely, ubiquitin-dependent protein catabolic process was associated proteins that are redistributed within post replication repair, post-translational protein modification, protein folding, ER stress and positive regulation of proteolysis and catabolic process (Supplementary Fig. S3B). The proteins associated with cell cycle-associated processes were linked with spindle organization, microtubule cytoskeleton organization, mitosis regulation of cell cycle phase transition mainly G2/M and G1/S (Supplementary Fig. S3C). The cell death node was implicated here with the regulation of intrinsic apoptotic signaling pathway contributed due to the response of oxidative, ER, and other toxic substances stress (Supplementary Fig. S3D). The GO term enriched with the regulation of peptidase activity and a few deubiquitinases, namely USP7, 10, and 14, were enriched with a significantly reduced expression.

Next, we checked the global ubiquitination pattern in HepG2 cells treated with PA and control vehicle (BSA) in a time-dependent manner. We observed an increase of low molecular, as well as high molecular weight protein ubiquitination at 12 h and 24 h time points upon exposure of PA, compare to its mock control (Fig. 2B). Mass spectrometry data identified USP7, USP10 and USP14 as downregulated deubiquitinating enzymes in PA exposure and both USP7 and USP10 play a critical role in mitosis(50,51). USP7 and USP10 have been shown to interact with p53 and Mdm2 (52,53) and prevent their proteasomal degradation. In a lipotoxic condition like NAFLD, USP10 is down-regulated and well-characterized in disease pathology(54). USP7 is also shown to be involved in hepatic de novo lipogenesis by transcriptionally regulating ZNF638(55). How does USP7 shift the equilibrium of cell death and cell survival balance in PA exposure is still poorly understood. In this note, USP7 may be a crucial player in the cell cycle, apoptosis, and ubiquitin signaling triad that induces lipid-induced cell death in HepG2 cells. Thus, we validated the expression of USP7 upon PA treatment. There was an increase in the expression of USP7 at 12 h which subsequently decreased in the later time points (Fig. 2C, D).

**Palmitic acid drives cell fate via downregulation of p53**

USP7 has been shown to regulate many cancers by modulating the p53-Mdm2 network(56). Thus, we sought to investigate the downstream consequence of lower expression of USP7 on p53 and Mdm2 levels. We found a reduced protein expression of p53 and Mdm2 in a time-dependent manner upon PA exposure (Fig. 3A-C). The depletion of p53 and Mdm2 may be due to their degradation via the ubiquitin proteasomal system. To test the p53 and Mdm2 stabilization, we co-treated the cells with a proteasome inhibitor (MG-132) and PA and checked their expressions. We observed a complete rescue of p53 and Mdm2 upon MG-132 treatment (Supplementary Fig. S4A, C, D). On the other hand, we also monitored the fate of USP7 upon PA treatment. Interestingly, we found the stabilization of USP7 upon MG132 treatment (Supplementary Fig. S4A, B). Thus, our results strongly support the destabilization and degradation of USP7, p53 and Mdm2 proceed through the ubiquitin-proteasome system in PA-induced conditions.

The regulation of p53 by USP7 is a complex phenomenon, and the dynamic relationship between USP7, p53, and Mdm2 is very much critical in cell survival and death. Here, we transiently overexpressed USP7 in HepG2 cells and the overexpressed cells were exposed to PA. The USP7 overexpressed HepG2 cells treated with BSA control showed a significant stabilization of both Mdm2 and p53. However, upon PA exposure, cells showed a substantial reduction of USP7 with subsequent destabilization of p53 and Mdm2 (Fig. 3D). The comparative analysis of PA treated empty vector control expressed HepG2 cells vs USP7 overexpressed cells with PA treatment showed the destabilization of Mdm2 and this destabilization may be due to its autoubiquitination and stabilization of p53 (Fig. 3D). Taken together, USP7 overexpression rescued the decline expression of p53 through an Mdm2 independent manner. Next, we lowered the expression of USP7 using siRNA and investigated the fate of p53 and Mdm2 upon PA exposure. siRNA knockdown of USP7 stabilized Mdm2 compared to non-targeting (NT) control cells (Fig. 3E). However, the expression of p53 did not change much. Upon PA treatment, there was a further reduction of USP7. Here we observed a significant reduction of both Mdm2 and p53. PA-induced loss of Mdm2 can be explained by its auto or any other E3 ligase-dependent ubiquitination leading to non-protection of ubiquitinated Mdm2 by USP7. On the other hand, the USP7 knockdown also stabilizes p53 that might be rescued from the action of Mdm2, E3 ligase dependent degradation, as compared to PA treated empty vector control (Fig. 3E).

Further, exposure to pharmacological inhibitors of USP7 also showed a rapid destabilization of Mdm2 both in control and PA treated conditions in a concentration-dependent manner. Conversely, PA-treated cells exhibited enhanced p53 stabilization as the concentration of inhibitors increased (Fig. 3F). Further, overexpression of catalytic inactive USP7 (C223S) also failed to recover the p53 stability irrespective of Mdm2 destabilization in PA treated HepG2 cells (Fig. 3G). Taken together, above all data suggest that USP7 acts on p53, independent of Mdm2, and Mdm2 destabilization is entirely independent of USP7 during PA toxicity. Thus, there might be some other ubiquitin ligase that facilitates the ubiquitination of Mdm2 during PA toxicity.

Checkpoints associated with molecules are well organized and channelized to ensure the proper cell synchronization and propagation on time. Alteration of protein quality control system deregulates checkpoints associated with protein expression. Therefore, we performed a cell cycle analysis using PI staining after PA treatment. A nice histogram of cell cycle distribution was shown at 0 h of untreated healthy cells (Fig. 3H). No significant changes were visualized in the control cells throughout all time (Supplementary Fig. S4E, F). However, we observed a 2.0- and 1.8-fold increase in cell number in the G2/M phase compared to the untreated condition at 12 h and 24 h, respectively (Fig. 3H, I). At later time points, particularly at 36 h and 48 h, cells mainly resided in the sub-G1 phase. A significant reduction of G0/G1 phases was noticed at 36 h and 48 h (Fig. 3H, I). Our results confirm that downregulation of USP7 potentiates the proteasomal degradation of p53 and dictates cell fate via cell cycle modulation.

**PA exhibits sequential variation in G2-M phase transition and mitotic abnormalities**

The G2/M checkpoints restrict mitosis onset upon exposure to many endogenous and exogenous materials, including chemotherapeutic drugs(57) and IR radiation(58). Thus, we verified DNA damage response markers γH2A.X(Ser139) and found more than 5-fold increased expression upon PA treatment (Fig. 4A). To understand the molecular interlayer’s of G2/M checkpoint recovery, we probed the expressions of G2/M cell cycle regulators, such as Wee1, pCdc2(Try15), and Cdc25C (Fig. 4A, B). The Wee1 expression was elevated by 1.5-fold at 12 h and gradually decreased in later time points. The lower expression of Wee1 along with phosphorylated CDK1 indicates an early CDK1 activation and supports the G2/M checkpoint recovery of cells (Fig. 4A, B). Interestingly, we also noticed a reduced expression of Cdc25C in PA treated conditions (Fig. 4B) which may be due to its proteasomal degradation. A few studies also reported that CDK1 activity may increase even after a deficiency in the CDK1-activating Cdc25A phosphatase(59). Further, USP7 has been shown to regulated Chk1-mediated cell cycle arrest by deubiquitinating and stabilizing Cdc25A in response to DNA damage(60,61). Thus, CDK1 inhibition rescue the generalized increase in p-histone3(Ser10) expression (Fig. 4A). Indeed, we found an almost 10-fold increased expression of p-H3(Ser10) upon PA exposure at 24 h and it supports premature mitotic entry (Fig. 4C).

Considering the significant reduction in expression of USP7, p53, Mdm2, Wee1, and other G2/M checkpoint proteins, role of PA in the mitotic delay needs detailed investigation. The flow cytometry analyses showed that PA led to a global accumulation of p-H3(Ser10) throughout all the time points except 48 h (Fig. 4D, E). Subsequently, our quantified data revealed a significant mitotic surge at 12 h with an almost 7-fold increase of the mitotic cell population. The mitotic cell population suddenly dropped at 24 h that endorsed a premature mitotic entry with damaged DNA (Fig. 4E). To circumvent the mitotic progression in the presence of PA, we fixed the HepG2 cells and performed immune-fluorescence staining against p-H3(Ser10). The confocal imaging data confirmed that PA potentially populated with p-H3(Ser10) positive cells compared to BSA treated control condition at 24h. Notably, majority of the mitotic cells restricted in aberrant metaphase onset leading to abnormal chromosome segregation and chromatin condensation (Fig. 4F). We calculated the mitotic index in two consecutive time points, 24 and 48 h. In the early time point, no significant changes were seen in the mitotic index due to treatment while significantly dropped by 2-fold at 48 h compared to 24 h in PA treated condition (Fig. 4G). The possible outcomes for drastic reduction due to mitotic anomalies in metaphase, PA prohibited a successful completion of cell division.

To observe the mitotic consequences of PA toxicity, we employed time-lapse live cell imaging in 0.5mM PA treated HepG2 cells that transiently expressed H2B mCherry to visualize the chromosomes. Time-lapse imaging data analysis showed that PA treated HepG2 cells expended significant delay in metaphase to anaphase transition compared to control cells. Control cells progressed from metaphase to anaphase (M-A) within 25 mins whereas PA-treated cells took more than 60 mins on an average (Fig. 5A, E). PA exhibited almost two times increase (%) of cell number that showed M-A transition delay compared to control (Fig. 5D). We further investigated the cause of M-A delay by investigating other mitotic phenotypes including chromosome mis-congression and improper segregation of sister chromatids. PA treated cells showed almost threefold increase of chromosome mis-congression than control cells leading to the delay in metaphase onset (Fig. 5B). We also probed into chromosome segregation defects once chromosomes are aligned at the metaphase plate. Interestingly, we found almost 4-fold increase of such defect than control condition and several cells showed a lagging chromosomes upon PA treatment (Fig. 5C, Supplementary Video. 1-4).

**Phosphoproteomics study also confirms PA-induced altered mitosis**

Intending to characterize signaling network alterations associated with cell division mainly mitosis, we used LC-MS/MS-based phosphoproteomics to dissect the *in vitro* temporal phosphoproteome dynamics into PA induced conditions (Supplementary Fig. S5A). We observed an increase in enriched phosphoproteins along with phosphosites over the time course of PA treatment and more than 20,000 phosphopeptides were identified throughout all-time points (Supplementary Fig. S5B). To represent a deep phosphoproteome coverage, we listed those proteins which were present in at least two biological replicates. We identified ~200 phosphoproteins enriched at each time point and 54 proteins were common in all-time points (Supplementary Data. 6, Supplementary Fig. S5C). Further, Gene Set Enrichment Analysis (GSEA) and Reactome pathway analysis of identified phosphorylated proteins identified Rho-GTPase cycle, metabolism associate proteins, membrane trafficking, unfolded proteins response, and cell cycle regulation as top 10 molecular pathways. Detailed analysis of each time point highlighted a dynamic regulation of phosphorylation and kinase action (Supplementary Data. 7, Fig. 5A-E). PA abrogated cytoskeleton reorganization was observed by robust activation of RHOBTB/ RHOBTB2 GTPase and Rho GTPase signaling pathways at all time points that ultimately alter membrane fluidity and plasticity. Interestingly, a few selective pathways were orchestrated uniquely in the individual time point which can be extrapolated throughout the time scale. Our phosphoproteomics data also supported that autophagy and aggrephagy are the consequences of early and prolong exposure of PA. Together, an apparent wave of coordinated phosphorylated proteins were observed in enriched cell cycle pathways across the period and implicated substantial alteration of cell fate upon PA treatment. The cell cycle-regulated phospho-proteins dynamics revealed that PA induced cell proliferation was observed in the early time points and it subsequently reduced at 12 and 18 h time. However, we observed an altered cell cycle related phosphoproteome signatures at 24 h. A few clusters were overrepresented with central cell cycle effectors and most of these cell cycle-regulated phosphosites fall within the mitotic phase (Fig. 5E). Further, major mitotic phosphoproteins such as NPM1, DYNC1H1, PCM1, HSP90AA1, CEP135, CEP110, and PCNT represented a wide range of Reactome categories related to mitosis like mitotic prometaphase, AURKA activation by TPX2, and loss of proteins required for interphase microtubule organization from the centrosome. In summary, our time dependent phosphoproteome analysis provides evidence of kinases and their substrates associated majorly with cell cycle regulation.

**Phosphoproteome analysis identified NPM1 as a regulator of mitotic catastrophe**

Next, we identified a kinase-substrate relationship from the phosphoproteome datasets using a web-based tool, KEA2 and constructed a radial map of diverse phosphoproteins with their respective kinase partners (Fig. 7A). Time dependent kinase distribution and their significant enrichment values were listed (Supplementary Data. 8 and Supplementary Fig. S5E-I). Our time-resolved phosphoproteome analysis recognized nucleophosmin1 (NPM1) that regulates its function by phosphorylation and dephosphorylation during the cell cycle (Fig. 7A). Interestingly, we found 12 phosphorylation sites in NPM1 in our high-throughput phospho-proteome dataset. Residues at Ser-125, Thr-199, Thr-219, and Thr-234/237 were phosphorylated by AURKB CDK1, CDK2, GSK3B, which are the regulators of the cell cycle. The phosphorylation site mapping revealed that phosphorylated Ser-112 and Ser-125 were dominant at 6, 18 and 24 h and absent at 12 h. We observed Thr-199 site phosphorylation at 12 h which correlates early G2/M checkpoint recovery followed by enhanced mitosis. The CDK2 mediated phosphorylation of NPM1 on Thr-199 also causes the dissociation of NPM1 away from the centrosome and permits duplication during mitosis(62). In parallel, 12 h phosphoproteome network also enriched with CDK1 dependent phosphorylation at Thr-219, Thr-234, and Thr-237 and thus entirely drive mitosis by abolishing the RNA binding activity of NPM1(63). Furthermore, the dephosphorylations on the above sites Thr-199, Thr-234/237 were more pronounced in later time points like 18 h and 24 h. Our kinase-substrate interaction map represents a strong mitotic phosphorylation marker, phospho-NPM1 (Thr-199, Thr-237, Thr-234) at 12 h, phosphorylated by CDK2 and CDK1 at the onset of mitosis(64–66) (Fig. 7A). Further, the phosphorylation at Ser-125 position by Aurora A and B at the mitotic peak supported its entry from the nucleus to mitochondria(67).

Further to quantify the ectopic NPM1 expression upon PA, western blot analysis was performed, and we found a slightly elevated expression at 12 h. In contrast, an almost 2-fold reduced expression of NPM was observed at 48h (Fig. 7B, C). Besides, we also observed 1.5-fold elevated expression in our quantitative differential proteomics data at 18h (Supplementary Table. 1, Fig. 2A). Further, immune fluorescence assay has also observed a wide-ranging cellular localization of NPM, depending on the extent of PA severity in HepG2 cells. NPM was localized in the nucleus in the control condition (green signal entirely merged with DAPI). However, NPM relocated into nucleoplasm upon PA exposure and finally in the cytoplasm. A yellow tint was visualized outside the nucleus where cellular cytoskeleton dye phalloidin co-localized with cytosolic NPM (Fig. 7D-F). Thus, our phosphoproteome along with confocal microscopy analysis confirm NPM as a regulator of mitotic catastrophe in PA exposed condition.

**Palmitic acid promotes AIF mediated cell death in HepG2 cells**

Mitotic catastrophe is delineated with cellular devastation that drives the cells towards an irreversible fate, either apoptosis or regulated necrosis(68). Here, we aim to investigate both caspase-dependent and caspase-independent pathways to find the possible outcome of PA persuaded mitotic catastrophe. The lethal proteins, such as BCL-2 family members Bax, Bid, and cleaved Cas3 and Cas9 were unchanged in the early time points and their expression was significantly reduced after 24 h of PA exposure (Fig. 8A-C). The data suggested that PA-induced HepG2 cell death is caspase as well as pro-apoptotic proteins independent. Despite homeostatic suppression of caspase-dependent cell death, a large cell population further entered into the early and late apoptosis as observed in our annexin V -FITC assay (Supplementary Fig. S1C, D). Therefore, we explored caspase-independent death pathways where cells response to intrinsic clues and evolve mitochondrial outer membrane permeabilization, membrane potential loss, PARP activation and release mitochondrial apoptosis inducing factor 1 (AIFM1) followed by its nuclear translocation and chromatolysis. The mitochondrial membrane potential analysis showed an almost 4-fold loss in PA treated condition compared to control at 24 h (Supplementary Fig. S6A). We also found a significant cleaved PARP in the later phase (24 h and 48 h) of PA exposure which was absent in the early time point (Fig. 8A, D). PARP activation also promotes the truncation of AIFM1 and translocates it into the nucleus. We observed 2 and 3.75-fold increase of cleaved AIFM1 at 24 and 48 h respectively in PA treated conditions compared to control (Fig. 8A, E). Further, subcellular fractionation of mitochondria and nucleus at four consecutive time points (0, 12, 24, and 48 h) followed by western blot analysis of AIFM1 and CypA also showed an increased nuclear concentration of tAIF and CypA at 24 h of PA exposure (Supplementary Fig. S6B).

To investigate the role of USP7 in caspase-dependent and independent cell death upon PA exposure, USP7 overexpressed, siRNA knockdown, pharmacological USP7 inhibitors and overexpression of catalytic inactive USP7 mutant conditions were subjected to the western blot analysis. In USP7 overexpressed condition, truncation of AIF (tAIF) and cleavage of Cas3 both were observed, whereas vector control expressed HepG2 cells did not show any of them (Fig. 8F). However, upon PA exposure USP7 overexpressed cells showed significant enhancement of AIF truncation and Cas3 cleavage compared to cells that were only exposed to PA. Interestingly, PA-treated USP7 overexpressed cells obscured the Cas9 cleavage (Fig. 8F). The death assessment through FACS demonstrated a significant increase of cell death population over the BSA treated while treated with PA in USP7 overexpressed condition (Fig. 8J). Taken together, USP7 overexpressed cells executed caspase-dependent and caspase-independent death through p53 stabilization during PA toxicity. Further, siRNA-mediated silencing of USP7 overcome the AIF truncation, Cas3/9 cleavage while, PA exposure prevailed the AIF truncation as well as caspases (Cas3/9) cleavage. Hence, PA exposure decreases the rate of AIF truncation and instead increases p53-mediated cleavage of Cas3/9 upon silencing compared to only PA-treated cells (Fig. 8G). Further, knockdown efficacy contributed to a drastic increase in the cumulative dead cell population (Fig. 8J).

PA treated catalytic inactive USP7 mutant (C223S) overexpressed cells rescued from advanced lethality due to overexpressed or knockdown of WT-USP7, while dealing with PA. Thus, no significant changes in AIF truncation and Cas3/9 cleavage compared to only PA treatment (Fig. 8H). Conversely, chemical inhibitors exacerbated AIF truncation and Cas3/9 cleavage in PA treated cells in concentration-dependent manner (Fig. 8I). As a result, inhibitors-mediated enhanced death, were executed through both caspase-dependent and independent manner (Fig. 8J).

Next, the co-localization of AIFM1 with nuclear resident DNA damage marker γH2A.X(Sre139) also supported tAIF’s translocation (Supplementary Fig. S6C-E). To better understand the role of AIF in PA-induced cellular changes, we performed immunoprecipitation of AIF interactors in BSA and PA treated conditions. The LC-MS-based interatomic analysis identified 96 proteins that showed differential abundance in the PA stimulated condition. Based on the expression pattern of candidate proteins and their rank, we identified the top 10 proteins that showed an interaction with AIF (Fig. 9A). Interestingly we observed nucleophosmin (NPM1) as one of the interactors and the AIFM1-NPM1interaction was further confirmed using immunoprecipitation followed by western blot analysis (Fig. 9A, B). The PA-induced AIFM1 release might be associated with NPM1 mediated loss of mitochondrial permeability and efficiently interact either in mitochondria or in the nucleus. Besides, an interaction of AIFM1 with CypA(69) and CypA-γH2A.X(Ser139) was also perceived in PA stimulated conditions which eventually evoked AIF-induced caspase-independent cell death (Fig. 9A, B, and Supplementary Fig. S6F, G). Furthermore, double immunofluorescence staining of HepG2 cells treated with PA against AIF and NPM also showed a considerable degree of co-localization for AIF (green) within the nuclear region (blue, DAPI) and NPM (pink) within mitochondria (red) in PA treated HepG2 cells. Interestingly, still few AIFM-NPM co-localization was observed upon PA exposure to HepG2 cells at 24 h (Fig. 9C). However, we did not see significant co-localization of AIF and NPM at 48 h (Fig. 9D). This cytoplasmic appearance coincided with a decrease in nuclear levels of NPM protein level, thus lowering the co-localization with AIF at the end stage of lipotoxicity. To conclude, our study suggested that PA promotes caspase-dependent cell death as an early event and prolong exposure to PA stimulates caspase-independent cell death mediated through AIFM1.

**Discussion**

PA-induced dysregulation of protein quality control largely hampers the ubiquitin-dependent protein catabolic process that ultimately modulates the cell cycle and triggers cell death. In addition, PA-toxicity is constantly challenged with heightened ubiquitinated protein load due to proteasome subunit modulation and altered expression of deubiquitinating enzymes. Our proteomics data show a considerably lower expression of a few deubiquitinases like USP7, USP10 and USP14. USP14 is one of the major proteasome-associated deubiquitinating enzymes and plays a critical role in disease conditions such as hyperglycemia(70) and heapato steatosis(71). Besides, USP7 is also well known for its antagonist action towards p53 and its regulatory ubiquitin ligase Mdm2(52). Further, pharmacological inhibition of USP7 shows significant anti-cancer activity through p53 dependent and independent manner(72,73). Recently, USP7 has been shown to play a critical role in pathological hepatic de novo lipogenesis(55). The downregulation of USP7 upon PA exposure destabilizes Mdm2 and p53 through their proteasomal degradation. Pieces of evidence suggest that the inhibition of USP7 activity or depletion of USP7 from cells causes defects in cell cycle progression(50,74). Our proteomic data identified DNA replication licensing factors MCM 2, 3, 5, and 7 down regulated in PA treated conditions and it has been shown that USP7-depleted cells show the delayed cell cycle progression due to defect in the MCM proteins(75). A few recent studies have also indicated mitotic phase cell cycle regulation by USP7 where USP7 promotes mitotic entry by stabilizing Polo-like kinase 1 (PLK1) and cells with USP7 depletion cause G2/M cell cycle arrest and delayed mitotic progression(76–78). Interestingly, the USP7-p53-Mdm2 axis facilitates abrogated G2/M checkpoint recovery through early CDK1 activation, phosphorylation of histone3(Ser 10) and γH2A.X(Ser 129) expression which eventually provisions the cells for a mitotic surge with damaged DNA. Further, USP7 depleted cells exhibit altered chromosomal misalignment and segregation during metaphase and anaphase transition that is the primary cause of chromosomal abnormalities(77,78). We demonstrate that PA-induced depletion of USP7 delays mitotic events, restricting the cells in pro-meta/metaphases. The PA-induced abrogated DNA damage checkpoint recovery followed by mitotic anomalies is the sign of the cellular catastrophe. Our temporal phospho-proteomics data shows the phosphorylation and dephosphorylation dynamics of nucleophosmin1 (NPM1) which regulates the cell cycle in many ways. Of note, the ectopic expression of NPM1 in HepG2 cells has been reported in PA-induced insulin resistance condition(79) and our data also corroborate with mitotic cell cycle regulation by NPM1 through phosphorylation at T199 upon PA exposure.

Given the above outcome, mitotic catastrophe (MC) is delineated with cellular devastation and it constitutes a crossroad for two conventional death pathways either in a caspase-dependent or caspase-independent(80–82). The mitotic catastrophe also occurs in p53-null cells where it operates by modulating caspases, AIF, autophagy and necroptosis(83,84). Sometimes cell death executes due to mechanical disruption which is induced by centrosome overduplication without much affecting the suicidal signal(83). Our data reveals that rapid depletion of lethal pro-apoptotic proteins Bax, Bid, and cleaved caspases (Cas3 and Cas9) expressions observed upon PA exposure as a late event of PA toxicity. On the other hand, prolonged PA exposure to cells potentiates PARP and AIFM1 cleave and drives the cell in caspase independent death. Persistent exposure of PA also induces cleave of AIF from mitochondria and facilitates the translocation of truncated AIF (tAIF) to nucleus through its interaction with CypA. They form a DNA-degrading complex with γH2A.X(Ser 139) which provokes cellular lethality. Further NPM1 acts as a BAX chaperon in leukemic conditions and aggravates stress-induced mitochondrial membrane injury and cell death(85). Finally, our data suggest that ectopic accumulation of NPM1 in the mitochondria promotes PA-induced loss of mitochondrial membrane potential in the absence of p53 and induces mitotic catastrophe and cell death.

In summary, our integrated analysis of quantitative proteomics and global phospho-proteomics showed an alteration in cell cycle checkpoints proteins expression and imbalanced kinome networks elicit cell cycle aberration in lipotoxic conditions. The abrogated G2/M checkpoints recovery with damaged DNA induces mitotic catastrophe that prepares cells for ultimate death in a p53 independent manner. Our findings uncovered a new direction in the PA-induced cell death through mitotic catastrophe independent of caspases where USP7, NPM1 and AIFM1 play a decisive role.

**Materials and methods**

**Cell line and palmitic acid treatment**

HepG2, hepatocellular carcinoma cell lines were cultured in high glucose Dulbecco's Modified Eagle's Medium (Gibco™ Life technologies, cat no. 12800017) supplemented with 1X antibiotic antimycotic solution (Himedia, REF A002A-100ML), 10% fetal bovine serum (Gibco™ Life technologies, cat no. 10270), and were kept in a humidified atmosphere of 5% CO2 incubator at 37 °C. Cells (4.4×10 ^6^) were plated in 100 mm cell culture dishes at least 24 h before treatment. Cells were authenticated by STR profiling and tested for mycoplasma contamination before experiments. Cells were exposed to different concentrations of FFAs (palmitic acid) in media containing 1% FFAs-free BSA (Sigma-Aldrich, cat no. A7030). Palmitic acid (ACROS-organics) was dissolved in 100% ethanol to their desired concentrations and the mixture were incubated at a temperature 65°C for 30 min followed by vigorous vortexing. Meanwhile, 10% BSA were prepared in 1X PBS (pH 7.4) at 55°C. Both solutions were mixed in a 1:20 ratio to prepare BSA conjugated palmitic acid solutions. The solutions were incubated at 55°C for 30 min with frequent vortexing. The mixture was cooled at 25°C and filtered by sterilizing a 0.45 µm syringe filter and stored at -20 °C until use. Finally, BSA conjugated palmitic acid was mixed with incomplete DMEM media and exposed to the cells for the treatment. The exact amount of 10% BSA conjugated ethanol was used to treat the cells in the control condition.

**Plasmids, siRNA and inhibitor treatments**

Plasmids coding for USP7 (pCDNA3.1-N-Myc_WT USP7) was a kind gift from R. Woodgate, and pCDH-CMV-H2B-mCherry-EF1-Hygromycon were taken from SVS Mylavarapu. USP7 catalytic mutant construct (C223S) was generated by standard SDM cloning Procedure. The mutant plasmid was verified by DNA sequencing. All transfections were conducted using Lipofectamine 3000 reagent (Invitrogen) following the manufacturer's instructions. A single siRNA was designed to knock down the potential expression of USP7. Non-target siRNA oligonucleotides (D-001810-10-20) were used for the control, and the oligos human siUSP7-CUAAGGACCCUGCAAAUUA was used against USP7. For successful siRNA delivery, we used the DharmaFECT 1 (Dharmacon/Thermo Scientific) transfection reagent (T-2001-02) for 48h. The overexpression and knockdown efficiency was confirmed by western blot. DUB inhibitors P22077 (662142) and P5091 (SML0770) were purchased from Sigma-Aldrich. MG132 (M7449) and nocodazole (M1404) were purchased from Invitrogen and Sigma.

**Cell viability test by MTT assay**

HepG2 cells were plated at an initial density of 8×10^3^ cells/well in a 96-well plate and treated with 0.25, 0.5, 0.75, 1.0, 2.0- and 3.0 mM concentrations of PA for 6, 12, 18, 24, 36, and 48 h time points. The experiments were done in triplicates. After incubation, 10 μl of MTT (Sigma Aldrich, USA) solutions were added to each well to a final concentration of 0.5 mg/ml and incubated for 4 h at 37 °C. Finally, the formazan crystals were dissolved in 100 μl of DMSO and the absorbance’s of coloured product were measured at 570 nm using ELISA plate reader (SpectraMax M5, Molecular Devices). The percentage of the cell viability was calculated as “Absorbance of sample-Absorbance of blank/ Absorbance of control -Absorbance of blank) x 100”. The IC_50_ value of palmitic acid treatment was calculated using GraphPad Prism 7.00 software.

**Oil red O staining**

HepG2 cells were grown at an initial density of 10^5^ cells/well in a 24-well plate and treated with 0.5 mM concentration of PA for 18 h. Cells were then washed three times with iced 1XPBS and fixed with 4% paraformaldehyde for 10 minutes. After fixation, cells were washed with PBS three times and stained with oil red O solution (working solution, 0.5 g oil red O powder (MPI) dissolved in 60% isopropanol) for 15 min and hematoxylin for 1 min at room temperature. Cells were rewashed with PBS to remove unbound staining. Bright-field microscopy was used to capture the image.

**Annexin V-FITC assay**

Quantification of apoptotic cell death upon PA treatment was determined by Annexin V-FITC assay according to the manufacturer's instructions (APOAF - Annexin V-FITC Apoptosis Detection Kit, Sigma-Aldrich) followed by flow cytometry analysis. In Brief, HepG2 cells (10^6^ cells) were trypsinized and harvested in cold Phosphate-buffered saline (PBS, pH 7.4) solution. Cells were resuspended in binding buffer and stained with 5 μl of Annexin V-FITC Conjugate and 10 μl of propidium iodide solution and incubated in the tubes at room temperature in dark for exactly 10 minutes. Cells were immediately subjected to flow cytometry analysis using BD FACSVerse^TM^ (BD Biosciences, San Jose, CA, USA). For quantifying the apoptotic population, FACS data were analysed by FlowJo 7.6 software. Further cell death assessment studies were also performed in (i) USP7 overexpressed (ii) USP7 knockdown (iii) chemical inhibitors for USP7 (P5091 and P22077) treated HepG2 cells. Data were acquired for control vehicle (CV), and PA treated conditions separately.

**Sample preparation for proteomics analysis**

Differential proteomics were carried out using iTRAQ labeling coupled with LC-MS/MS. HepG2 cells were grown in high glucose DMEM media in T-25 flasks and PA treatment were done at their 70% confluence. Cells were exposed to “control vehicle” and “treated” conditions for 18h. After incubation, cells were washed three times with chilled 1X PBS and proteins were extracted from the harvested cell pellets by RIPA lysis buffer (Sigma, cat no. R0278) containing 1X halt-protease and phosphatase inhibitor cocktail (Cat no.1861282, Thermo Fisher Scientific, USA). The cells were mechanically lysed by mild sonication with intermediate vortexing. The lysates were centrifuged at 14,000×g for 30 min at 4°C and the supernatant was collected. All the impurities present within the samples, like SDS, NP-40 were removed by acetone precipitation. The protein precipitates were re-solubilized in 8 M urea. The protein concentration was estimated by the BCA method (Pierce™ BCA Protein Assay Kit, Thermo Scientific).

**iTRAQ labeling and fractionation by reverse phase chromatography**

From each condition, an equal amount of protein (100 μg) was subjected for reduction (10 mM DTT, 56^0^C, 30 min) and alkylation (freshly prepared 20 mM IAA, 30 min, dark). The urea concentration was adjusted up to 1M with 500 mM triethylammonium bicarbonate (TEAB) and MS grade trypsin (Pierce™ Trypsin Protease MS grade, cat no. 1862743, USA) was added to digest the proteins into peptides (trypsin: protein ratio of 1:20 (w/w) at pH 8, 37°C for 24 h). The digestion was terminated by adding formic acid (1% v/v). Samples were dried in a vacuum centrifuge and resuspended in 500 mM TEAB buffer before isobaric tagging for relative and absolute quantification (iTRAQ labelling). The samples were labelled with isobaric tags iTRAQ 4-plex kit as the manufacturer's instructions (AB Sciex, USA). The labelling strategy with isobaric mass tags was as follows: control (untreated) samples with mass tags 114 & 116 and palmitic acid-treated samples with mass tag 115 & 117. The samples were incubated at room temperature for 2 hrs for complete labelling. After incubation, the samples were mixed 1:1:1:1 and dried by vacuum centrifugation. Two biological repeats were carried out.

To reduce the proteome complexity, combined labelled peptides were employed for high pH RP chromatography. Peptide mixtures were separated by a 1260 Infinity high-pH reverse-phase LC system (Agilent, USA). A total of 400 μg combined labeled peptides were loaded onto the C18 column (Agilent, USA, 300 extend-C18; 3.5 µm; 2.1 × 150 mm), which was previously equilibrated with solvent A (10 mM ammonium formate, pH 10) and the column temperature was maintained at 40°C. The 60 min linear gradient was composed of 96% buffer A for 1 min; 4–19% buffer B (98% ACN, 2% ddH_2_O, pH 10.0) for 30 min; then 19–95% buffer B for 23 min; followed by 95% buffer B for 5 min at a flow rate of 0.5 ml/min. The eluted fractions were pooled to 11 fractions for each biological replicate. The fractions were vacuum dried and desalted with C18 tips (Pierce^TM^ C18 Tips, USA cat no. 87784), and stored at -20°C until MS analysis.

**LC-MS/MS acquisition, protein identification, quantification, and functional analysis**

Peptide fractions were suspended in buﬀer A (0.1% FA, 2% ACN) and analyzed by in Sciex 5600^+^ Triple-TOF mass spectrometer coupled with ChromXP reversed-phase 3 μm C18-CL trap column (350μm × 0.5 mm, 120 Å, Eksigent) and nanoViper C18 separation column (75 μm × 250mm, 3 μm, 100 Å; Acclaim Pep Map, Thermo Scientific) in Eksigent nanoLC (Ultra 2D plus) system. The binary mobile solvent system was used as follows: solvent A (2% (v/v) ACN, 0.1% (v/v) FA in water) and solvent B (98% (v/v) ACN, 0.1% (v/v) FA). The peptides were separated at a 300 nl/min flow rate in a 60 min gradient with a total run time of 75 min. Data were acquired in information-dependent mode with a TOF/MS survey scan (400–1250 m/z), where the accumulation time was ~250 ms. For fragmentation, a maximum number of twenty precursor ions per cycle was selected and each cycle consist of 100 ms acquisition time for MS/MS scan range of 100–1600 m/z. The parent ions with a charge state from +2 to +5 were included for the MS/MS fragmentation. The MS/MS spectra were acquired in high sensitivity mode with adjust collision energy when using iTRAQ reagent settings.

All raw files (.wiff) were converted to Mascot generic file (mgf) format using Peak View (version 1.2.0.3). ProteinPilot software (version 4.5, SCIEX) with the Paragon algorithm was used for protein identification and relative iTRAQ quantification. Proteins were identified against the UniProt human-reviewed database containing only canonical sequences (downloaded in July, 2018). The search parameters were set as follows: (a) iTRAQ 4plex (peptide labelled) (b) IAA Cysteine alkylation; digestion enzyme Trypsin. Peptides and proteins were validated at ˂1% false discovery rate (FDR) and with unused score ˃ 1.3 (which corresponds to ˃ 95% confidence). Proteins were considered only if they were significant in all independent biological replicate experiments. The ratio threshold for up-regulation and down-regulation proteins was set to *>*1.3 or <0.7 respectively with *p*-value *<*0.05. The average expression values of replicates were used to indicate the absolute protein abundance at a given sample. The altered proteins were further analysed using WebGestalt(46), ClueGo(86) for Gene Ontology (GO) enrichment and KEGG, Reactome for pathway enrichment analysis. Final data were visualized by using Cytoscape software (Version 3.8.2)(87).

**Western blot analysis**

The whole protein lysates were prepared in RIPA lysis buffer (Sigma-Aldrich, USA) supplemented with a 1X halt-protease and phosphatase inhibitor cocktail. Total protein content in the lysates was measured using BCA method (Pierce™ BCA Protein Assay Kit, Thermo Scientific). From each condition, 20-30 μg of protein lysates were resolved in 10-15% SDS-PAGE and proteins were transferred from gel to PVDF membrane (Immobilon-P, PVDF, 0.45µm, Merck Millipore) by wet transfer method at 4°C for 90 min at a constant voltage (100V) using Bio-Rad transfer apparatus. After transfer, the membrane was rinsed with TBS followed by blocking with 5% skimmed milk powder (dissolved in TBS+0.1% Tween20) for 1 h at room temperature on a rocker. The membrane was then rinsed once with TBST to remove excess skimmed milk solution. Immunoblotting analyses were performed by incubating the blot with the specific primary antibody: USP7 (3277, CST, 1:1000 and ab4080, Abcam, 1:2000), p53 (9282, CST, 1:1000), Mdm2 (86934, CST, 1:1000), Wee1 (13084, CST, 1:1000), pCdc2(Try15) (4539, CST, 1:1000), Cdc25C (4688, CST, 1:1000), γH2A.X (Ser 139)(9718, CST, 1:1000), p-H3(Ser10) (3377, CST, 1:1000), AIF (4642, CST, 1:1000), NPM (3532, CST, 1:1000), Caspase3 (9662, CST, 1:1000), Caspas9 (9502, CST, 1:1000), PARP (9532, CST, 1:1000), Bax (2772, CST, 1:1000), Bid (2002, CST, 1:1000), β-Actin (4967, CST, 1:1000), Ubiquitin (sc 271289, Santa Cruz Biotechnology, 1:1000), CYCS (PAA594Hu01, Cloud-Clone Corp, 1:1000), CypA (PAA979Mu01, Cloud-Clone Corp, 1:1000), COX4I1 (PAD286Hu01, Cloud-Clone Corp, 1:1000), H3 (PAA285Hu01, Cloud-Clone Corp, 1:1000) and GAPDH (398600, Invitrogen, 1:5000) for overnight at 4^0^ C. Secondary immunoblotting was done using secondary antibodies {rabbit (31460, Invitrogen, 1:10000) and mouse (SAA544Mu19, Cloud-Clone Corp, 1:8000)} for 1 hr at room temperature under constant shaking condition. Further, three washes (5 min each) were given with TBST. Protein signals were developed with Luminata ECL reagent (Immobilon Forte, Millipore, USA) and visualized by Gel illuminator (Chemi Doc^TM^ MP Imaging System, Bio-rad). Images were processed using Image Lab (6.0.1.) software for quantitative analysis.

**Cell cycle analysis and mitotic entry by flow cytometry**

The cell cycle analysis was examined by the flow cytometric method. In brief, HepG2 cells were treated with 0.5 mM palmitic acid and harvested at 0-48 h (each 12 h interval), where control vehicle was used as a control for each time point. Harvested cells were washed twice with 1X phosphate-buffered saline (PBS, pH 7.4) and fixed in ice-cold 70% ethanol for overnight at 4 °C. Ethanol-fixed cells were centrifuged and washed twice with 1X PBS. The cell pellet was resuspended in 1X PBS (300 µl) along with ribonuclease (50 µl from 100 µg/ml stock solution) and propidium iodide (200 µl from 50 µg/ml stock solution) and incubated at 37 °C for 30 min. To calculate the mitotic cell percentage, fixed cells were stained with p-H3(Ser10) (PA517869, Invitrogen, 1:50) for 1 h at room temperature in continuous shacking conditions. Stained cells were washed twice with PBS and incubated with Alexa-488 conjugated secondary goat anti-rabbit IgG (A11034, Invitrogen-Molecular Probes, 1:700). After secondary staining, cells were washed twice with PBS and resuspended with PBS containing PI/RNase staining before flow cytometry analysis. Cell cycle and p-H3 staining data were acquired on BD FACSVerse^TM^ (BD Biosciences, San Jose, CA, USA). The data were analyzed by FlowJo 7.6 software.

**Microscopy**

**Confocal imaging and image analysis**

Palmitic acid (0.5 mM) and control vehicle were given to the HepG2 cells as discussed above and a co-localization study was performed to analyse proteins distributions at two different time points (24 and 48 h). Briefly, upon treatment cells were incubated with Mito Tracker at 200 nM concentration according to the manufacturer's instructions (MitoTracker Red CMXRos, M7512, Invitrogen – Molecular Probes) and washed the cells with 1X PBS (pH=7.4) for three times. 4% paraformaldehyde was used for fixing cells on the coverslips for 10 min. After fixation, cells were washed with 1X PBS and incubated with 1X PBS containing 1% BSA and 0.1% Triton-X 100 (PBAT) for 1h for permeabilization and blocking the non-specific epitopes. Further, the cells were incubated at 4°C overnight with primary antibody AIF (4642, CST, 1:100), γH2A.X(Ser139) (9718, CST, 1:400), p-H3(Ser10) (3377, CST, 1:800), NPM (ab10530, Abcam, 1:500), CypA (PAA979Mu01, Cloud-Clone Corp, 1:200) and α-Tubulin (13-8000, Invitrogen, 1:250). After incubation, cells were washed thrice with PBSAT. The coverslips were incubated with corresponding secondary antibodies including goat anti-rabbit Alexa 488 (A11034, Invitrogen-Molecular Probes, 1:700) and goat anti-mouse Alexa 594 (A11005, Invitrogen-Molecular Probes, 1:800) and 647 (715-605-150, Jackson ImmunoResearch, 1:800)) for 1 h. Phalloidin dye (12381, Invitrogen – Molecular Probes, 1:40) was added to the cells during secondary antibody incubation. Cells were rinsed thrice with PBAT followed by 1X PBS and counterstained with the DNA-binding dye DAPI (Sigma-Aldrich). Mounting reagent ProLong™ Gold Antifade Mountant (P36934, Thermo Fisher Scientific) was used for preserving the coverslips. The images were acquired using a 63× lens with 1.5× zoom using a confocal laser-scanning microscope (Leica TCS SP8 II system). The light was collected through an oil immersion objective. Offline Leica LAS X software module was used for post imaging processing, and ImageJ (Fiji) software was used for measuring the mitotic index. Mitotic index was calculated as (number of cells in mitosis (p-H3(Ser10 positive)/ total number of cells (stained by DAPI)) ×100 under the microscopic field.

**Time-lapse imaging and image analysis**

HepG2 cells transiently expressing H2B mCherry were grown on coverslips for 36h. Further, cells were treated with 400nM nocodazole for 16h to enrich their populations in prometaphase, washed twice with PBS and sophistically placed on a custom-designed aluminium slide containing chamber provided with either control vehicle (CV) or 0.5mM PA treatment media, respectively. Time-lapse imaging was performed for 12 hours with ~5 min intervals between successive frames at different positions on each coverslip in the bright field and fluorescence modes using a 63x1.4 numerical aperture oil immersion lens in an environmentally controlled chamber with 5% CO2 and 37^0^C (Reference?). The movies were acquired using a confocal laser-scanning microscope (Leica TCS SP8 II system). Time-lapse imaging data analysis was performed on an offline Leica LAS X software module. Quantification of chromosome congression, metaphase to anaphase delay, and lagging chromosomes were evaluated from time-lapse series of HepG2 cells transiently expressing H2B mCherry plasmid.

**Sample preparation for phosphoproteomics analysis**

Phosphoproteomic analysis was carried out using metal oxide affinity enrichment (MOAC) coupled with LC-MS/MS. For this, HepG2 cells were incubated with 0.5 mM palmitic acid for 0, 6, 12, 18, and 24 h time points, where 0h signifies the untreated condition, used as a control. After completion of each time point, cells were harvested and proteins were extracted by RIPA lysis buffer (Sigma, cat no. R0278) supplemented with 1X halt-protease and phosphatase inhibitor cocktail (Cat no.1861282, Thermo Fisher Scientific, USA) with intermediate vortexing, followed by mild sonication. The lysate was centrifuged at 14,000× g for 30 min at 4°C and the supernatant was collected. Six volumes of acetone were added to the lysates for protein precipitation. The protein precipitates were re-solubilized in 8 M urea and concentrations were estimated by BCA method (Pierce™ BCA Protein Assay Kit, Thermo Scientific).

**Protein digestion and Phospho-proteome enrichment**

From each condition, an equal amount of protein (10 mg) was incubated with 10 mM DTT (56^0^C, 30 min) for reduction and alkylated with 20 mM IAA (room temperature, 30 min, dark). MS grade trypsins (Sigma Aldrich, USA) were added for digestion (trypsin: protein ratio of 1:20 (w/w) at pH 8, 37°C for 24 h) and reactions were quenched by adding 1% formic acid. Following digestion, the resulting peptide mixtures were centrifuged at 14000×g for 30 min to remove the undigested particles. The supernatant was collected and loaded onto the reversed-phase C18 Sep-Pak cartridges (Cat no. WAT020515, Waters, USA) for desalting and clean-up of peptides. The peptides were vacuumed dry and kept at -80^0^C until enrichment.

Phospho-peptides enrichment was done by titanium dioxide beads (TiO_2_). TiO2 beads were pre-incubated in 2,5-dihydroxybenzoic acid (20mg/ml) in 80% ACN and 1% FA (5 µl/mg of beads) for 20 min. Dried peptides were resuspended in 80% ACN and 5% FA in a final volume of 10 ml. Then beads were incubated for 30 min while rotating. After incubation, each condition was centrifuged at 4000×g for 5min and supernatant was removed. Beads were washed thrice with 30% ACN and 1%FA, 50% ACN and 1%FA and 80% ACN and 1%FA. Phosphorylated peptides were eluted with 100 µl of 5% NH_4_OH followed by 100 µl 10% NH_4_OH with 25% ACN. Eluted peptides were dried in SpeedVac, desalted with C18 tips (Pierce, Thermo Fisher Scientific, USA) and primed for LC-MS/MS analysis.

**LC-MS/MS analysis of enriched phosphopeptides**

Dried phospho-peptides were reconstituted with solvent A (2% (v/v) ACN, 0.1% (v/v) FA in water) and performed LC−MS/MS experiments using Sciex 5600^+^ Triple-TOF mass spectrometer coupled with ChromXP reversed-phase 3 μm C18-CL trap column (350μm × 0.5 mm, 120 Å, Eksigent, AB Sciex) and nanoViper C18 separation column (75 μm × 250mm, 3 μm, 100 Å; Acclaim Pep Map, Thermo Scientific, USA) in Eksigent nanoLC (Ultra 2D plus) system. The binary mobile solvent system was used as follows: solvent A (2% (v/v) ACN, 0.1% (v/v) FA in water) and solvent B (98% (v/v) ACN, 0.1% (v/v) FA). The peptides were separated using a 60 min gradient with a total run time of 90 min at a flow rate of 300 nl/min. The MS data of each condition was acquired in IDA (information-dependent acquisition) with high sensitivity mode. Each cycle consisted of ~250 and 100 ms acquisition time for MS1 (m/z 350−1250 Da) and MS/MS (100–1600 m/z) scans respectively, with a total cycle time of ~2.8s. Each condition was run in triplicate.

All spectral raw files (.wiff) were searched using “ProteinPilot” software (version 4.5, SCIEX) with the Mascot algorithm (version 2.3.02) for protein identification against the SwissProt_57.15 database (20266 sequences after Homo sapiens taxonomy filter). The search parameters for the identification of phosphorylated peptides were as follows: (a) trypsin as a proteolytic enzyme (with up to two missed cleavages); (b) peptide mass error tolerance of 20 ppm; (c) fragment mass error tolerance of 0.20 Da; and (d) carbamidomethylation of cysteine (+57.02146 Da), oxidation of methionine (+15.99492 Da), deamination of NQ (+0.98416), Phospho (ST) (79.96633 Da), and Phospho (Y) (79.96633) as variable modifications.

Phophoproteins were finalized with at least one corrected phosphorylated peptide having PEP score ≥ 10 PEP which was extracted from the smooth gaussian curve. Next, time-resolved phosphoproteins list were finalized based on proteins with their presence in minimum two biological replicates. Functional enrichment analyses were executed using two web-based tools, "GSEA (<https://www.gsea-msigdb.org/gsea/index.jsp)>(88), and Reactome pathway browser (<https://reactome.org/PathwayBrowser/#TOOL=AT>)(89). For visualization of respective pathways, PPI networks were generated using STRING(90). The phosphosites were retrieved from PhosphoSitePlus(91). A web-based tool, KEA2 (<https://www.maayanlab.net/KEA2/>)(92) were used to correlate the kinase-substrate relationship from the phosphoproteome datasets and a yFiles radial map of diverse phosphoproteins with their respective kinase partner were constructed using Cytoscape (version 3.8.2)(87) across the PA induced time course.

**Mitochondrial membrane potential (ΔΨm)**

The mitochondrial membrane potential (ΔΨm) was measured using JC-10 dye for determining the loss of the MMP upon PA treatment. In brief, HepG2 cells were seeded in a 96-well plate and treated with 0.5 mM concentrations of PA for 24 h and subjected for staining according to the manufacturer's instructions (Sigma-Aldrich, Mitochondrial Membrane Potential Kit. (Cat no. MAK159)). JC-10 was formed fluorescent aggregates according to the polarization of the mitochondrial membrane upon palmitic acid treatment. Fluorescence intensity (lex = 490/ lem = 525 nm) and (lex = 540/lem = 590 nm) were monitored for ratio analysis using ELISA plate reader (SpectraMax M5, Molecular Devices). The ratio of red/green fluorescence intensity was calculated using GraphPad Prism 7.00 software.

**Subcellular fractionation followed by western blot analysis**

Subcellular fractionation of HepG2 cells were done at 0, 12, 24 and 48h time points for separating the mitochondria and nucleus. Mitochondrial and nuclear fractionation protocols were followed according to the manufacturer's instructions (Mitochondria Isolation Kit for Cultured Cells (Thermo Scientific™ **cat no.** 89874) and NE-PER™ Nuclear and Cytoplasmic Extraction Reagents (Thermo Scientific™, **cat no.** 78833)) and the respective fractions were subjected further for immunoblotting analysis according to the western blot methodology as described above.

**Interactomic analysis using LC-MS/MS followed by Co-IP**

HepG2 were treated with control vehicle and 0.5mM palmitic acid for 24 h. Cells were harvested followed by trypsinizaton and total protein was extracted in 500 µl of IP lysis buffer (Pierce, Thermo Scientific) containing 1X halt-protease and phosphatase inhibitor cocktail (Thermo Fisher Scientific, USA) with intermediate vortexing and sonication. The lysates were centrifuged at 14,000× g for 30 min at 4°C and aliquot of supernatants were quantified by BCA method (Thermo Fisher Scientific, USA). Equivalent amount (1 mg) of proteins from each condition was incubated with anti-AIF conjugated protein G Sepharose 4 fast flow beads (17-0618-01/ GE Healthcare) for overnight at 4°C. The immunoprecipitated complex was washed three times with IP lysis buffer and bound protein complexes were eluted in 50 µl SDS-PAGE Laemmli sample buffer. The samples were fractionated on 12% SDS-PAGE and stained with Coomassie stain (0.1% w/v). The gel was used further in-gel mass spectrometry analysis.

Gel slices corresponding to each lane were cut out and destained using wash buffer (50 mM ammonium bicarbonate (ABC) and 50% ACN). Slices were hydrated with 10 mM DTT (56^0^C, 30 min), alkylated using 50 mM IAA (30 min, RT, dark) and further dehydrated using 100% ACN before digestion. The available proteins in gel pieces were digested with MS grade trypsin (Pierce™ Trypsin Protease MS grade, cat no. 1862743, USA) and incubated at 37°C for 18 h. The extraction of digested peptides was done by adding 60% ACN with 0.1% FA and 100% ACN with 0.1% FA, followed by ultra-sonication. The digested peptides were collected, vacuum dried, and resuspended in 35 μl of solvent A (2% (v/v) ACN, 0.1% (v/v) FA in water) and subjected for LC−MS/MS experiments using Sciex 5600^+^ Triple-TOF mass spectrometer coupled with ChromXP reversed-phase 3 μm C18-CL trap column (350 μm × 0.5 mm, 120 Å, Eksigent, AB Sciex) and nanoViper C18 separation column (75 μm × 250 mm, 3 μm, 100 Å; Acclaim Pep Map, Thermo Scientific, USA) in Eksigent nanoLC (Ultra 2D plus) system. The binary mobile solvent system was consisted with solvent A (2% (v/v) ACN, 0.1% (v/v) FA in water) and solvent B (98% (v/v) ACN, 0.1% (v/v) FA). The peptides were separated with 200 nl/min flow rate in a 60 min gradient with a total run time of 90 minutes. The acquisition was executed with conventional data-dependent IDA mode. The parent spectra were acquired with the scan range of 350-1250 m/z. The data-dependent acquisition experiments were set to obtain a high-resolution TOF–MS/MS scan over a mass range of 100-1800 m/z. The raw spectrum was run on a MaxQuant (v1.6.1.0)(93) with default settings against human database. The identification settings were used as follows: (a) trypsin as a proteolytic enzyme (b) Oxidation (M); Acetyl (Protein N-term); Carbamidomethyl (C); Deamidation (NQ) set as variable modifications. Proteins were listed down for individual conditions.

**Quantification and statistical analysis**

Statistical analysis was performed using the GraphPad Prism 7 software. The results were presented as the standard error means (SEM). The graphs represented N≥3 independent experiments. Ordinary one-way and two-way ANOVA (Tukey's multiple comparison test, Sidak's multiple comparisons test), unpaired two-tailed t-test, Mann-Whitney two-tailed t-test or adjusted t-tests were used for calculating the statistical significance. P values less than 0.05 were significant and indicated by asterisks as follows: *P < 0.05, **P < 0.01, ***P < 0.001, ****P < 0.0001.

**Data availability**

## The proteomics data is available online through the ProteomeXchange Consortium via the PRIDE (<https://www.ebi.ac.uk/pride/>) partner repository with the dataset identifiers PXD029010, PXD029016, and PXD029005 for iTRAQ dataset, phosphoproteome dataset and AIF interactome dataset respectively.

**References**

1. Brookheart RT, Michel CI, Schaffer JE. As a matter of fat. *Cell Metab* **10**, 9-12 (2009)

2. Shimabukuro M, Zhou YT, Levi M, Unger RH. Fatty acid-induced β cell apoptosis: a link between obesity and diabetes. *Proc Natl Acad Sci USA* **95**, 2498-502 (1998)

3. Rada P, González-Rodríguez Á, García-Monzón C, Valverde ÁM. Understanding lipotoxicity in NAFLD pathogenesis: is CD36 a key driver?. *Cell Death Dis* **11**, 1-5 (2020)

4. Wen H, Gris D, Lei Y, Jha S, Zhang L, Huang MTH, et al. Fatty acid–induced NLRP3-ASC inflammasome activation interferes with insulin signaling. *Nat Immunol* **12**, 408-15 (2011)

5. Zhou YT, Grayburn P, Karim A, Shimabukuro M, Higa M, Baetens D, et al. Lipotoxic heart disease in obese rats: implications for human obesity. *Proc Natl Acad Sci USA* **97**, 1784-9 (2000)

6. Chaurasia B, Summers SA. Ceramides–lipotoxic inducers of metabolic disorders. *Trends Endocrinol Metab* **26**, 538-50 (2015)

7. Shin SJ, Lim JH, Chung S, Youn DY, Chung HW, Kim HW, et al. Peroxisome proliferator-activated receptor-α activator fenofibrate prevents high-fat diet-induced renal lipotoxicity in spontaneously hypertensive rats. *Hypertens Res* **32**, 835-45 (2009)

8. Erbay E, Babaev VR, Mayers JR, Makowski L, Charles KN, Snitow ME, et al. Reducing endoplasmic reticulum stress through a macrophage lipid chaperone alleviates atherosclerosis. *Nat Med* **15**, 1383-91 (2009)

9. Browning JD, Horton JD. Molecular mediators of hepatic steatosis and liver injury. *J Clin Invest* **114**, 147-52 (2004)

10. Ipsen DH, Lykkesfeldt J, Tveden-Nyborg P. Molecular mechanisms of hepatic lipid accumulation in non-alcoholic fatty liver disease. Cell Mol Life Sci **75**, 3313-27 (2018)

11. Leamy AK, Egnatchik RA, Young JD. Molecular mechanisms and the role of saturated fatty acids in the progression of non-alcoholic fatty liver disease. *Prog Lipid Res* **52**, 165-74 (2013)

12. Yamaguchi K, Yang L, McCall S, Huang J, Xing XY, Pandey SK, et al. Inhibiting triglyceride synthesis improves hepatic steatosis but exacerbates liver damage and fibrosis in obese mice with nonalcoholic steatohepatitis. *Hepatology* **45**, 1366-74 (2007)

13. Lin L, Ding Y, Wang Y, Wang Z, Yin X, Yan G, et al. Functional lipidomics: Palmitic acid impairs hepatocellular carcinoma development by modulating membrane fluidity and glucose metabolism. *Hepatology* **66**, 432-48 (2017)

14. Levental KR, Malmberg E, Symons JL, Fan YY, Chapkin RS, Ernst R, et al. Lipidomic and biophysical homeostasis of mammalian membranes counteracts dietary lipid perturbations to maintain cellular fitness. Nat Commun **11**, 1-3 (2020)

15. Rong X, Albert CJ, Hong C, Duerr MA, Chamberlain BT, Tarling EJ, et al. LXRs regulate ER stress and inflammation through dynamic modulation of membrane phospholipid composition. *Cell Metab* **18**, 685-97 (2013)

16. Carta G, Murru E, Banni S, Manca C. Palmitic acid: physiological role, metabolism and nutritional implications. *Front Physiol* **8**, 902 (2017)

17. Zhang D, Tong X, Arthurs B, Guha A, Rui L, Kamath A, et al. Liver clock protein BMAL1 promotes de novo lipogenesis through insulin-mTORC2-AKT signaling. *J Biol Chem* **289**, 25925-35 (2014)

18. Ricchi M, Odoardi MR, Carulli L, Anzivino C, Ballestri S, Pinetti A, et al. Differential effect of oleic and palmitic acid on lipid accumulation and apoptosis in cultured hepatocytes. *J Gastroenterol Hepatol* **24**, 830-40 (2009)

19. Listenberger LL, Han X, Lewis SE, Cases S, Farese R V, Ory DS, et al. Triglyceride accumulation protects against fatty acid-induced lipotoxicity. *Proc Natl Acad Sci USA* **100**, 3077-82 (2003)

20. Koves TR, Ussher JR, Noland RC, Slentz D, Mosedale M, Ilkayeva O, et al. Mitochondrial overload and incomplete fatty acid oxidation contribute to skeletal muscle insulin resistance. *Cell Metab* **7**, 45-56 (2008)

21. Liu L, Xie B, Fan M, Candas-Green D, Jiang JX, Wei R, et al. Low-level saturated fatty acid palmitate benefits liver cells by boosting mitochondrial metabolism via CDK1-SIRT3-CPT2 cascade. *Dev Cell* **52**, 196-209 (2020)

22. Piccolis M, Bond LM, Kampmann M, Pulimeno P, Chitraju C, Jayson CBK, et al. Probing the global cellular responses to lipotoxicity caused by saturated fatty acids. *Mol Cell* **74**, 32-44 (2019)

23. Mota M, Banini BA, Cazanave SC, Sanyal AJ. Molecular mechanisms of lipotoxicity and glucotoxicity in nonalcoholic fatty liver disease. *Metabolism* **65**, 1049-61 (2016)

24. Paumen MB, Ishida Y, Muramatsu M, Yamamoto M, Honjo T. Inhibition of carnitine palmitoyltransferase I augments sphingolipid synthesis and palmitate-induced apoptosis. *J Biol Chem* **272**, 3324-9 (1997)

25. Chouchani ET, Kajimura S. Metabolic adaptation and maladaptation in adipose tissue. *Nat Metab* **1**, 189-200 (2019)

26. Turpin SM, Lancaster GI, Darby I, Febbraio MA, Watt MJ. Apoptosis in skeletal muscle myotubes is induced by ceramides and is positively related to insulin resistance. *Am J Physiol - Endocrinol Metab* **291**, E1341-50 (2006)

27. Chen Z, Tian R, She Z, Cai J, Li H. Role of oxidative stress in the pathogenesis of nonalcoholic fatty liver disease. *Free Radic Biol Med* **152**, 116-41 (2020)

28. Murphy MP. Mitochondrial dysfunction indirectly elevates ROS production by the endoplasmic reticulum. *Cell Metab* **18**, 145-6 (2013)

29. Wei Y, Wang D, Gentile CL, Pagliassotti MJ. Reduced endoplasmic reticulum luminal calcium links saturated fatty acid-mediated endoplasmic reticulum stress and cell death in liver cells. *Mol Cell Biochem* **331**, 31-40 (2009)

30. Özcan U, Cao Q, Yilmaz E, Lee AH, Iwakoshi NN, Özdelen E, et al. Endoplasmic reticulum stress links obesity, insulin action, and type 2 diabetes. *Science* **306**, 457-61 (2004)

31. Pfaffenbach KT, Gentile CL, Nivala AM, Wang D, Wei Y, Pagliassotti MJ. Linking endoplasmic reticulum stress to cell death in hepatocytes: roles of C/EBP homologous protein and chemical chaperones in palmitate-mediated cell death. *Am J Physiol - Endocrinol Metab* **298**, E1027-35 (2010)

32. Wang D, Wei Y, Pagliassotti MJ. Saturated fatty acids promote endoplasmic reticulum stress and liver injury in rats with hepatic steatosis. *Endocrinology* **147**, 943-51 (2006)

33. Pang L, Liu K, Liu D, Lv F, Zang Y, Xie F, et al. Differential effects of reticulophagy and mitophagy on nonalcoholic fatty liver disease. *Cell Death Dis* **9**, 1-3 (2018)

34. Feldstein AE, Canbay A, Angulo P, Taniai M, Burgart LJ, Lindor KD, et al. Hepatocyte apoptosis and fas expression are prominent features of human nonalcoholic steatohepatitis. *Gastroenterology* **125**, 437-43 (2003)

35. Akazawa Y, Cazanave S, Mott JL, Elmi N, Bronk SF, Kohno S, et al. Palmitoleate attenuates palmitate-induced Bim and PUMA up-regulation and hepatocyte lipoapoptosis. *J Hepatol* **52**, 586-93(2010)

36. Malhi H, Bronk SF, Werneburg NW, Gores GJ. Free fatty acids induce JNK-dependent hepatocyte lipoapoptosis. *J Biol Chem* **281**, 12093-101 (2006)

37. Li Z, Lai ZW, Christiano R, Gazos-Lopes F, Walther TC, Farese R V. Global analyses of selective insulin resistance in hepatocytes caused by palmitate lipotoxicity. *Mol* *Cell Proteomics* **17**, 836-49 (2018)

38. Li Z, Liu H, Niu Z, Zhong W, Xue M, Wang J, et al. Temporal proteomic analysis of pancreatic β-cells in response to lipotoxicity and glucolipotoxicity. *Mol Cell Proteomics* **17**, 2119-31 (2018)

39. Wood GC, Chu X, Argyropoulos G, Benotti P, Rolston D, Mirshahi T, et al. A multi-component classifier for nonalcoholic fatty liver disease (NAFLD) based on genomic, proteomic, and phenomic data domains. *Sci Rep* **7**, 1-9
(2017)

40. Maldonado EM, Fisher CP, Mazzatti DJ, Barber AL, Tindall MJ, Plant NJ, et al. Multi-scale, whole-system models of liver metabolic adaptation to fat and sugar in non-alcoholic fatty liver disease. *npj Syst Biol Appl* **4**, 1-0 (2018)

41. Tsuchida T, Lee YA, Fujiwara N, Ybanez M, Allen B, Martins S, et al. A simple diet-and chemical-induced murine NASH model with rapid progression of steatohepatitis, fibrosis and liver cancer. *J Hepatol* **69**, 385-95 (2018)

42. Van Herck MA, Vonghia L, Francque SM. Animal models of nonalcoholic fatty liver disease—a starter’s guide. *Nutrients* **9**, 1072 (2017)

43. Kubota N, Kado S, Kano M, Masuoka N, Nagata Y, Kobayashi T, et al. A high‐fat diet and multiple administration of carbon tetrachloride induces liver injury and pathological features associated with non‐alcoholic steatohepatitis in mice. *Clin Exp Pharmacol Physiol* **40**, 422-30 (2013)

44. Friedman SL, Neuschwander-Tetri BA, Rinella M, Sanyal AJ. Mechanisms of NAFLD development and therapeutic strategies. *Nat Med* **24**, 908-22 (2018)

45. Gao D, Nong S, Huang X, Lu Y, Zhao H, Lin Y, et al. The effects of palmitate on hepatic insulin resistance are mediated by NADPH Oxidase 3-derived reactive oxygen species through JNK and p38MAPK pathways. *J Biol Chem* **285**, 29965-73 (2010)

46. Liao Y, Wang J, Jaehnig EJ, Shi Z, Zhang B. WebGestalt 2019: gene set analysis toolkit with revamped UIs and APIs. *Nucleic Acids Res* **47**, W199-205 (2019)

47. Pagano G, Pacini G, Musso G, Gambino R, Mecca F, Depetris N, et al. Nonalcoholic steatohepatitis, insulin resistance, and metabolic syndrome: further evidence for an etiologic association. *Hepatology* **35**, 367-72 (2002)

48. Musso G, Cassader M, Gambino R. Non-alcoholic steatohepatitis: emerging molecular targets and therapeutic strategies. *Nat Rev Drug Discov* **15**, 249-74 (2016)

49. Montgomery MK, De Nardo W, Watt MJ. Impact of lipotoxicity on tissue “cross talk” and metabolic regulation. *Physiology* **34**, 134-49 (2019)

50. Giovinazzi S, Morozov VM, Summers MK, Reinhold WC, Ishov AM. USP7 and Daxx regulate mitosis progression and taxane sensitivity by affecting stability of Aurora-A kinase. *Cell Death Differ* **20**, 721-31 (2013)

51. Sun J, Li T, Zhao Y, Huang L, Sun H, Wu H, et al. USP10 inhibits lung cancer cell growth and invasion by upregulating PTEN. *Mol Cell Biochem* **441**, 1-7 (2018)

52. Sheng Y, Saridakis V, Sarkari F, Duan S, Wu T, Arrowsmith CH, et al. Molecular recognition of p53 and MDM2 by USP7/HAUSP. *Nat Struct Mol Biol* **13**, 285-91 (2006)

53. Yuan J, Luo K, Zhang L, Cheville JC, Lou Z. USP10 regulates p53 localization and stability by deubiquitinating p53. *Cell* **140**, 384-96 (2010)

54. Luo P, Qin C, Zhu L, Fang C, Zhang Y, Zhang H, et al. Ubiquitin‐specific peptidase 10 (USP10) inhibits hepatic steatosis, insulin resistance, and inflammation through Sirt6. *Hepatology* **68**, 1786-803 (2018)

55. Ni W, Lin S, Bian S, Zheng W, Qu L, Fan Y, et al. USP7 mediates pathological hepatic de novo lipogenesis through promoting stabilization and transcription of ZNF638. *Cell Death Dis* **11**, 1-7 (2020)

56. Cummins JM, Vogelstein B. HAUSP is required for p53 destabilization. *Cell cycle* **3**, 687-90 (2004)

57. Sakaue-Sawano A, Kobayashi T, Ohtawa K, Miyawaki A. Drug-induced cell cycle modulation leading to cell-cycle arrest, nuclear mis-segregation, or endoreplication. *BMC Cell Biol* **12**, 1-2 (2011)

58. Fernandez-Capetillo O, Chen HT, Celeste A, Ward I, Romanienko PJ, Morales JC, et al. DNA damage-induced G 2–M checkpoint activation by histone H2AX and 53BP1. *Nat Cell Biol* **4**, 993-7 (2002)

59. Ruiz S, Mayor-Ruiz C, Lafarga V, Murga M, Vega-Sendino M, Ortega S, et al. A genome-wide CRISPR screen identifies CDC25A as a determinant of sensitivity to ATR inhibitors. *Mol Cell* **62**, 307-13 (2016)

60. Vega IA De, Martín Y, Smits VAJ. USP7 controls Chk1 protein stability by direct deubiquitination. *Cell cycle* **13**, 3921-6 (2014)

61. Valles GJ, Bezsonova I, Woodgate R, Ashton NW. USP7 is a master regulator of genome stability. Front. *Cell Dev Biol* **8**, 717 (2020)

62. Okuwaki M. The structure and functions of NPM1/Nucleophsmin/B23, a multifunctional nucleolar acidic protein. *J Biochem* **143**, 441-8 (2008)

63. Tarapore P, Shinmura K, Suzuki H, Tokuyama Y, Kim SH, Mayeda A, et al. Thr199 phosphorylation targets nucleophosmin to nuclear speckles and represses pre-mRNA processing. *FEBS Lett* **580**, 399-409 (2006)

64. Tokuyama Y, Horn HF, Kawamura K, Tarapore P, Fukasawa K. Specific phosphorylation of nucleophosmin on Thr199 by cyclin-dependent kinase 2-cyclin E and its role in centrosome duplication. *J Biol Chem* **276**, 21529-37 (2001)

65. Ching RHH, Lau EYT, Ling PMT, Lee JMF, Ma MKF, Cheng BYL, et al. Phosphorylation of Nucleophosmin at Threonine 234/237 is associated with HCC metastasis. *Oncotarget* **6**, 43483 (2015)

66. Cha H, Hancock C, Dangi S, Maiguel D, Carrier F, Shapiro P. Phosphorylation regulates nucleophosmin targeting to the centrosome during mitosis as detected by cross-reactive phosphorylation-specific MKK1/MKK2 antibodies. *Biochem J* **378**, 857-65 (2004)

67. Falini B, Nicoletti I, Bolli N, Martelli MP, Liso A, Gorello P, et al. Translocations and mutations involving the nucleophosmin (NPM1) gene in lymphomas and leukemias. *Haematologica* **92,** 519-32 (2007).

68. Castedo M, Perfettini JL, Roumier T, Andreau K, Medema R, Kroemer G. Cell death by mitotic catastrophe: a molecular definition. *Oncogene* **23**, 2825-37 (2004)

69. Doti N, Reuther C, Scognamiglio PL, Dolga AM, Plesnila N, Ruvo M, et al. Inhibition of the AIF/CypA complex protects against intrinsic death pathways induced by oxidative stress. *Cell Death Dis* **5**, e993 (2014)

70. Liu B, Zhang Z, Hu Y, Lu Y, Li D, Liu J, et al. Sustained ER stress promotes hyperglycemia by increasing glucagon action through the deubiquitinating enzyme USP14. *Proc Natl Acad Sci USA* **116**, 21732-8 (2019)

71. Liu B, Jiang S, Li M, Xiong X, Zhu M, Li D, et al. Proteome-wide analysis of USP14 substrates revealed its role in hepatosteatosis via stabilization of FASN. *Nat Commun* **9**, 1-2 (2018)

72. Reverdy C, Conrath S, Lopez R, Planquette C, Atmanene C, Collura V, et al. Discovery of specific inhibitors of human USP7/HAUSP deubiquitinating enzyme. *Chem Biol* **19**, 467-77 (2012)

73. Yi L, Cui Y, Xu Q, Jiang Y. Stabilization of LSD1 by deubiquitinating enzyme USP7 promotes glioblastoma cell tumorigenesis and metastasis through suppression of the p53 signaling pathway. *Oncol Rep* **36**, 2935-45 (2016)

74. Agathanggelou A, Zlatanou A, Gulshanara A, Stewart G, Kearns PR, Pratt G, et al. New Therapeutic Strategy for Sensitisation of CLL Cells with Inactivation of the DNA Damage Response by Targeting the Deubiquitylating Enzyme USP7-Dependent Pathways. *Blood* **118**, 3865 (2011)

75. Jagannathan M, Nguyen T, Gallo D, Luthra N, Brown GW, Saridakis V, et al. A role for USP7 in DNA replication. *Mol cell biol* **34**, 132-45 (2014)

76. Yamaguchi L, Nishiyama A, Misaki T, Johmura Y, Ueda J, Arita K, et al. Usp7-dependent histone H3 deubiquitylation regulates maintenance of DNA methylation. *Sci Rep* **7**, 1-2 (2017)

77. Giovinazzi S, Sirleto P, Aksenova V, Morozov VM, Zori R, Reinhold WC, et al. Usp7 protects genomic stability by regulating Bub3. *Oncotarget* **5**, 3728 (2014)

78. Peng Y, Liu Y, Gao Y, Yuan B, Qi X, Fu Y, et al. USP7 is a novel Deubiquitinase sustaining PLK1 protein stability and regulating chromosome alignment in mitosis. *J* *Exp Clin Cancer Res* **38**, 1-2 (2019)

79. Wang X, Ma H, Wang X. Nucleophosmin/B23 contributes to hepatic insulin resistance through the modulation of NF-κB pathway. *Biochem Biophys Res Commun* **511**, 214-20 (2019)

80. Vakifahmetoglu H, Olsson M, Zhivotovsky B. Death through a tragedy: mitotic catastrophe. *Cell Death Differ* **15**, 1153-62 (2008)

81. Vitale I, Galluzzi L, Castedo M, Kroemer G. Mitotic catastrophe: a mechanism for avoiding genomic instability. *Nat Rev Mol Cell Biol* **12**, 385-92 (2011)

82. Prokhorova EA, Egorshina AY, Zhivotovsky B, Kopeina GS. The DNA-damage response and nuclear events as regulators of nonapoptotic forms of cell death. *Oncogene* **39**, 1-6 (2020)

83. Fragkos M, Beard P. Mitotic catastrophe occurs in the absence of apoptosis in p53-null cells with a defective G1 checkpoint. *PloS one* **6**, e22946 (2011)

84. Carrà G, Panuzzo C, Torti D, Parvis G, Crivellaro S, Familiari U, et al. Therapeutic inhibition of USP7-PTEN network in chronic lymphocytic leukemia: a strategy to overcome TP53 mutated/deleted clones. *Oncotarget* **8**, 35508 (2017)

85. Wang Z, Gall JM, Bonegio R, Havasi A, Illanes K, Schwartz JH, et al. Nucleophosmin, a critical Bax cofactor in ischemia-induced cell death. *Mol Cell Biol* **33**, 1916-24 (2013)

86. Bindea G, Mlecnik B, Hackl H, Charoentong P, Tosolini M, Kirilovsky A, et al. ClueGO: a Cytoscape plug-in to decipher functionally grouped gene ontology and pathway annotation networks. *Bioinformatics* **25**, 1091-3 (2009)

87. Otasek D, Morris JH, Bouças J, Pico AR, Demchak B. Cytoscape automation: empowering workflow-based network analysis. *Genome Biol* **20**, 1-5 (2019)

88. Subramanian A, Tamayo P, Mootha VK, Mukherjee S, Ebert BL, Gillette MA, et al. Gene set enrichment analysis: a knowledge-based approach for interpreting genome-wide expression profiles. *Proc Natl Acad Sci USA* **102**, 15545-50 (2005)

89. Jassal B, Matthews L, Viteri G, Gong C, Lorente P, Fabregat A, et al. The reactome pathway knowledgebase. *Nucleic Acids Res* **46**, D649-55 (2018)

90. Szklarczyk D, Gable AL, Lyon D, Junge A, Wyder S, Huerta-Cepas J, et al. STRING v11: protein–protein association networks with increased coverage, supporting functional discovery in genome-wide experimental datasets. *Nucleic Acids Res* **47**, D607-13 (2019)

91. Hornbeck P V, Kornhauser JM, Latham V, Murray B, Nandhikonda V, Nord A, et al. 15 years of PhosphoSitePlus®: integrating post-translationally modified sites, disease variants and isoforms. *Nucleic Acids Res* **47**, D433-41 (2019)

92. Lachmann A, Ma’ayan A. KEA: kinase enrichment analysis. *Bioinformatics* **25**, 684-6 (2009)

93. Cox J, Mann M. MaxQuant enables high peptide identification rates, individualized ppb-range mass accuracies and proteome-wide protein quantification. *Nat Biotechnol* **26**, 1367-72 (2008)

## **Acknowledgments**

## We appreciate continuous support by RCB technical staff of RCB mass spectrometry facility, microscopy facility in data collection. TKM thanks Regional Centre for Biotechnology for funding. SS and RV thank CSIR for the fellowship. CK thanks ICMR for the fellowship.

**Author Contribution**

TKM and SS conceived the project and designed the experiments. SS performed proteomics sample preparation, mass spectrometry data analysis and biochemical and cellular experiments. BK and AKD helped in MS data collection. RV prepared the mutant. RV and MS supported FACS data collection and analysis. TKM, SS, MVS and CK design the live cell imaging experiments. SS and CK performed live cell imaging and image analysis. SS and TKM wrote the manuscript. All author read and approve the final version of the manuscript.

**Competing interests:** The authors declare no competing interests.

**Supplementary information**

**Supplementary Figures legends:**

**Supplementary Fig. 1**: PA-induced cytotoxic effects in HepG2 cells.

**Supplementary Fig. 2:** Overview of differential proteomics.

**Supplementary Fig. 3:** Malfunction of metabolic activity evoked upon lipotoxicity.

**Supplementary Fig. 4:** PA-induced USP7/Mdm2/p53 axis destabilization.

**Supplementary Fig. 5:** Phosphoproteome analysis of HepG2 cells treated with PA.

**Supplementary Fig. 6:** PA potentiates to induce AIF-mediated caspase-independent cell death.

**Supplementary Table 1:** Lists of differentially expressed proteins.

**Supplementary Table 2:** Annotation of differentially regulated network dynamics connected cell cycle and death through ubiquitin-dependent protein catabolic process

**Supplementary Data 1:** Expression pattern of differentially expressed candidates within two biological replicates.

**Supplementary Data 2:** List of metabolic activity that enriched in our mass spec dataset.

**Supplementary Data 3:** Gene ontology analysis of differentially expressed proteins.

**Supplementary Data 4:** KEGG pathway enrichment analysis.

**Supplementary Data 5:** Reactome pathway enrichment analysis.

**Supplementary Data 6:** Overview of phosphoproteins analysis.

**Supplementary Data 7:** Kinase-Substrate enrichment with Phosphosites in different time points.

**Supplementary Data 8:** Top 10 Reactome pathway executed through phosphoproteome.

**Supplementary Data 9:** Overview of AIF interatomic proteome in PA induced condition.

**Supplementary Movie 1:** Confocal fluorescence time-lapse movie in control vehicle (CV) treated HepG2 cells transiently expressing H2B-mCherry (red) within a chromosome, showing the duration of mitosis.

**Supplementary Movie 2:** Confocal fluorescence time-lapse movie upon 0.5mM PA treatment in HepG2 cells transiently expressing H2B-mCherry (red), showing the duration of mitosis. The chromosomes showed congression defects at metaphase and lagging chromosomes during metaphase to anaphase transition.

**Supplementary Movie 3:** Confocal fluorescence time-lapse movie represents 0.5mM PA-induced chromatin condensation and fragmentation (chromatinolysis) due to incapacitation of successful mitosis.

**Supplementary Movie 4:** Confocal fluorescence time-lapse movie showing 0.5mM PA-induced chromosomal mis-congression that ultimately bypasses the metaphase. Further, unequal chromosomal segregation during anaphase leads to chromatin fragmentation, condensation, and finally preparing the cell for death through mitotic catastrophe.

**Figures legends**

**Fig. 1: Differential proteomics reveals altered protein quality control upon Palmitic acid treatment to HepG2 cells.**

Schematic representation of differential proteomics analysis of HepG2 cells upon PA exposure at 18 h. BSA conjugated palmitic acid (0.5 mM) and BSA were used as “PA treatment” and control vehicle respectively. The experimental workflow includes protein extraction, acetone precipitation, tryptic digestion, four-plex iTRAQ labeling, peptide fractionation by basic C18-RP-HPLC, LC-MS/MS data collection in Tiple TOF 5600 and data analysis using Protein Pilot software 4.5.0.0 (A). Venn diagram represented differentially expressed proteins (DEPs) within two biological replicates (B). The volcano plot distinguished the fold change value >1.3 and <0.7 with p-value <0.05 as upregulation (red dot) and down-regulation (green dot) respectively (C). Over-Representation Analysis (ORA) using the KEGG pathway (functional database) enrichment analysis illustrated highly altered biological events (top12) upon PA exposure. The percentage of signature visualizes denoted pathways for each term (D). Functional analysis of differentially expressed proteins network was made using Reactome pathway database. Representative pathways are mapped according to the total associated genes in each term and color code denotes significant value from dark to light (E). All results were created with ClueGo v2.5.7. Two-sided hypergeometric statistical test was used for both ontology clustering. The P-value cut-off was <0.05 and Bonferroni step-down correction method was used in both analyses. Kappa score threshold was set at 0.5.

**Fig. 2: Palmitic acid deregulates checkpoint-associated DUBs in HepG2 cells.**

Annotation of differentially regulated network dynamics connected cell cycle and death through ubiquitin-dependent protein catabolic process. The nodes with DEPs of three major networks are represented in PA-induced conditions. Each candidate was represented as a dot where green and red denote downregulated upregulated protein respectively. Over-represented GO-terms and their matching candidates were retrieved from STRING v11 and reconstructed by Cytoscape (A). Time-dependent ubiquitination pattern of HepG2 upon 0.5 mM PA and control vehicle treatment was executed at 0, 12, 24, and 48 h time points. HepG2 cells without any treatment were considered as 0 h. GAPDH was used as a loading control (B). PA partially regulated USP7 in HepG2 cells. Same lysates were subjected to SDS-PAGE and immune-blotted with USP7 antibody(C). In blot, an untreated cell at 0 h signifies a healthy condition. GAPDH was used as a loading control. Each time-dependent expression was normalized by GAPDH, and fold change intensity was normalized by individual BSA treated conditions of a particular time point. Quantification of protein expression was represented by dot plot (D). Ordinary one-way ANOVA followed by Tukey's multiple comparisons test was applied for statistical analysis. Error bars are showing mean±SEM; n≥ 3; *p ≤ 0.05; **, p ≤ 0.01; ****, p ≤ 0.0001, and ns=non-significant.

**Fig. 3: Palmitic acid drives cell fate via downregulation of p53.**

PA efficiently down-regulates the expression of p53 and Mdm2. Western blot of total HepG2 cell lysate from control vehicle (BSA) and 0.5 mM PA in three different time points (12, 24, and 48 h). 0 h lysate put as a healthy cell condition and immunoblotted against p53 and Mdm2 (A). GAPDH was used as a loading control. Proteins were quantified as a normalized fold change density compared to individual BSA treated conditions of a particular time point (B and C). Ordinary one-way ANOVA followed by Tukey's multiple comparisons test was applied for statistical analysis. USP7 stabilizes p53 through its deubiquitinating activity. Western blot analysis of HepG2 cells transiently expressing WT-USP7 were treated with 0.5mM PA for 24h showing the dynamic expression pattern of Mdm2 and p53. The overexpression of USP7 rescued declined expression of p53 through Mdm2 independent manner (D). Pre-silencing of USP7 in 0.5mM PA treated HepG2 cells for 24h was subjected to Mdm2 and p53 expression analysis through western blot. The knockdown efficiency completely destabilizes the Mdm2 and partially stabilizes p53 that is rescued from Mdm2, E3 ligase dependent degradation (E). Further, western blot analyses of Mdm2 and p53 were performed in PA treated HepG2 cells prior exposed to pharmacological inhibitors of USP7 for 24h. Pre-treatment of both (P22077 and P5091; 10 and 20µM) inhibitors showed rapid stabilization of p53 followed by a reduction in Mdm2 expression in a concentration-dependent manner upon 0.5mM PA exposure for 24h in the HepG2 cells (F). Consistently, overexpression of catalytic inactivated mutant (C223S-WT-USP7) fails to recover the p53 stabilization irrespective of Mdm2 de-stability in 24h PA HepG2 (G). PA regulates G2/M checkpoints recovery. HepG2 cells were harvested upon PA (0.5 mM) exposure. The cell cycle analysis was performed on incubation of cells with propidium iodide (50 µg/ml) for 30 min in dark (H). The percentage of cell distribution through all phases was presented in the bar graph (I). Cell cycle distribution mapping was performed every 12 h interval till up to 48h after post-treatment of PA. Two-way ANOVA followed by Tukey's multiple comparisons test was applied for statistical analysis. Error bar represents mean±SEM; n≥ 3, *p<0.05, **p<0.01, ***p<0.001, ****p<0.0001, and ns=non-significant.

**Fig. 4: PA exhibits sequential variation in G2-M phase transition and mitotic abnormalities.**

PA modulates the G2/M checkpoints associated markers. HepG2 cells lysates were harvested at 0, 12, 24, and 48 h consecutive time points after 0.5 mM PA treatment, and BSA was used as control. Western blot analysis was performed against Wee1, pCdc2 (Try15), Cdc25C, γH2A.X (Ser139), and p-H3(Ser10). GAPDH was an internal control (A). All protein expressions were quantified as a normalized fold change density compared to individual control conditions of a particular time point. Bar graph showing the quantification of immunoblots (B). A dot plot of quantified H3S10P protein expression was represented in a time-dependent manner (C). Two-way ANOVA followed by Tukey's multiple comparisons test was applied for both statistical analyses. PA regulates early G2/M checkpoint recovery. PA (0.5 mM) treated fixed cells were stained with propidium iodide and p-H3(Ser10) Alexa 488 in 0, 12, 24, 36 and 48 h time points for mitotic entry analysis. The percentage of mitotic cells in each condition was represented in flow cytometry windows which denoted p-H3(Ser10) positive cells with 4N DNA contents (D). Each treated condition was compared with the respective control vehicle. The percentage of mitotic cells for each condition was quantified and visualized by a bar graph. Ordinary one-way ANOVA followed by Tukey's multiple comparisons test was used for statistical analysis (E). PA restricts the cells in mitosis irrespective of successful cell division. Representative immunofluorescence images of BSA and PA treated HepG2 cells showed chromatin-bound p-H3(Ser10) (green), α-tubulin (red), and DAPI (blue). The overlay channels for all merged conditions were shown on the right panel: scale bar, 10 µm (F). Mitotic cells are indicated by white arrows. The mitotic index (MI) of individual conditions was measured by the. percentage of stained cells (positive green signals of p-H3(Ser10)) against viable cells, counting the nucleus (DAPI signal) by ImageJ software (Fiji). Bar graph showing the percentage of mitotic cells in each condition. Each dot denotes the MI of a particular field. Ordinary one-way ANOVA followed by Tukey's multiple comparisons test was used for statistical analysis. All error bar represents as mean±SEM; n≥ 3; *p<0.05, **p<0.01, ***p<0.001, ****p<0.0001, and ns=non-significant.

**Fig. 5 PA exhibits abnormal chromosomal segregation alongside congression defects.**

Stills from time-lapse videos featuring transiently expressed H2B-mCherry in HepG2 cells that were treated with control vehicle (upper lane) and 0.5mM PA (lower last two-lane). Images ensure a stipulated time frame for cell division in both cases. Cells treated with PA showed chromosomal congression defects and metaphase to anaphase delay. Yellow arrows show an unaligned chromosome. Along with the delay in metaphase to anaphase transition, lagging chromosomes (indicated by white arrows) were also observed, which is a hallmark of aberrant chromosomal segregation, during PA-induced lipotoxicity (A). Quantify the fraction of mitotic cells showing chromosome congression defects upon PA treatment(B). A representative graph shows the percentage of lagging chromosomes within a fraction of mitotic cells (C). The number of cells showing metaphase (M) to anaphase(A) delay was quantified (D), followed by measuring the delay in times (mins) (E). For B-D, individual dots represent value from individual biological replicates. Unpaired two-tailed t-test were applied for statistical analysis. The graphs represent N=3 independent experiments, with a minimum of 12 mitotic cells per experiment. In addition, each dot in scatter plot E represents the M-A transition period for one cell. A total of 36 mitotic cells were subjected to analyzing the PA-induced M-A delay. Mann-Whitney two-tailed t-test was applied for statistical analysis. Error bars represent mean±SEM; Scale bar, 25 µm; *p<0.05, **p<0.01, ***p<0.001, ****p<0.0001, and ns=non-significant.

**Fig. 6: Phosphoproteomics study also confirms PA-induced altered mitosis.**

Represented histogram showing top 10 enriched functional Reactome pathways that were arranged respective to their gene numbers, pValue (-Log2), and FDR(-Log10) ratio across the 0, 6, 12, 18, and 24h time points (upper panel) (A)-(E). Schematic representations of the above-enlisted pathways corresponding to their linked phosphoproteins were highlighted. Each pathway was embedded with an associative PPI network generated from STRING with the highest confidence level (0.900). The coloured domain represents individual pathways respective to their course of incubation time with PA (lower panel) (A)-(E).

**Fig. 7: Phosphoproteome analysis identified NPM1 as a regulator of mitotic catastrophe.**

A pictorial illustration of kinase-substrate interaction map throughout five-time points (0, 6, 12, 18, and 24h). The time points (define each node with individual colour) were associated with phosphoproteins (middle shell) and their respective phosphosites arranged in the outer shell. The image was drawn in Cytoscape by using yFiles radial layout. Colour gradient denoted the presence of those particular phosphoproteins among different time points (A). PA dynamically changes NPM expression. Western blot analysis of HepG2 cells was performed upon 0.5 mM PA treatment at 0 h 12 h, 24 h, and 48 h (B). GAPDH was used as a loading control. Proteins were quantified as a normalized fold change density compared to individual control conditions of particular time points. Ordinary one-way ANOVA followed by Tukey's multiple comparisons test was applied for statistical analysis (C). PA alters the NPM localization in a time-dependent manner. Immunofluorescence analysis demonstrating NPM (green) distribution between the nucleus (blue, DAPI) and cytosol (red, MITO TRACKER) in HepG2 cells upon 0.5 mM PA treatment. The overlay channel for all merged conditions was shown on the right panel. Scale bar, 10 µm (D). Bar graph showing quantification of co-localization of Pearson’s value for NPM and DAPI (E). The representative bar graph represents the cellular distribution ratio of NPM (nucleus/ cytosol) (E). The translocation ratio 2 fold reduced upon PA treatment at 24 h. Ordinary one-way ANOVA followed by Tukey's multiple comparisons test was applied for statistical analysis. All error bars are showing mean±SEM; n≥3; *p<0.05, **p<0.01, ***p<0.001, ****p<0.0001, and ns=non-significant.

**Fig. 8: Palmitic acid promotes AIF mediated cell death in HepG2 cells.**

PA potentiates caspase-independent cell death over caspase-dependent. PA-induced HepG2 cells were harvested at 0 h, 12 h, 24 h, and 48 h time points. The caspase-dependent molecules like c-Cas3, c-Cas9, Bax, and Bid and caspase-independent molecules like CypA, c-PARP, and tAIF were probed in western blot analysis (A). GAPDH was used as a loading control. Proteins were quantified as a normalized fold change density compared to individual control conditions of particular time points (B, C, D, and E). Two-way ANOVA followed by Sidak's multiple comparisons test was applied for statistical analyses. USP7/p53 axis exacerbates Palmitic acid induced AIF mediated cell death. A group of HepG2 cells that were transiently expressed WT-USP7, siRNA USP7, catalytic inactive mutant (C223S) of USP7 and pretreated with pharmacological inhibitors, P22077 and P5091 (10 and 20 µM for 24h) were treateded with or without palmitic acid (0.5mM) for 24h and were subjected for immunoblot assay. Caspases dependent (cCas3 and cCas9) and caspase-independent (AIF truncation) both were explored for death assessment. USP7 overexpression elevated cell death through AIF truncation and Cas3 cleavage upon PA treatment (F). Pre-silencing of USP7 abrogated cellular lethality while treated with PA. Thus, PA-treated USP7 knockdown cells efficiently showed both caspase-dependent as well as caspase-independent cell death (G). However, catalytic mutant of USP7 (C223S) failed to execute the USP7 lethality during PA toxicity (H). Chemical inhibitors exacerbated PA-induced cell death (I). Immunoblots were normalized by GAPDH. Further, the above groups were treated with or without PA (0.5mM) for 24 h and applied to annexin V-FITC assay; the percentage of death assessment due to alterations of USP7 levels upon PA induction was revealed by FACs analysis(J). Representative statistical results were performed using ordinary one-way ANOVA followed by Tukey’s multiple comparison test and shown as mean±SEM; n ≥ 3; *p<0.05, **p<0.01, ***p<0.001, ****p<0.0001, and ns=non-significant.

**Fig. 9: AIFM1/NPM1axis operates during PA-induced cell death.**

Identification of potent AIFM1 interacts upon PA stimulation. Scatter diagram showing the AIFM1-interacting proteins in HepG2 after treatment with control vehicle or PA (0.5 mM). The y axis shows the Log2 fold change (PA/ BSA) and the x-axis shows protein rank abundance. Each point represents a protein. Red dots denote the top 10 highly interacting, and blue indicates poorly interacting partners of AIFM1 in both BSA and PA-treated conditions (A). The endogenous interactions between AIF and NPM were determined by co-immunoprecipitation (Co-IP) assay in PA (0.5 mM) treated HepG2 cells at 24 h. Blots were developed against AIF, NPM and CypA. GAPDH was used as an internal control for input (B). The cellular distribution of NPM1 upon PA stimulation. Immunofluorescence analysis demonstrated NPM (pink) translocation from the nucleus to mitochondria (red) where cells were close to death, followed by AIF (green) translocated to the nucleus (blue) in treated (0.5 mM PA) condition. The localization dynamics were observed at two different time points (24 h and 48 h). The Pearson coefficient has been measured for the co-localization of AIF with DAPI and NPM with MITO TRACKER. The overlay channel for all merged conditions was shown on the right panel with a scale bar of 10 µm (B). Co-localization quantification was performed using Pearson’s value for AIF and NPM (D). Ordinary one-way ANOVA followed by Tukey's multiple comparisons test was applied for statistical analysis. Error bars are showing mean±SEM; n ≥ 3; *p<0.05, **p<0.01, ***p<0.001, ****p<0.0001, and ns=non-significant.

**Figure:1**

**
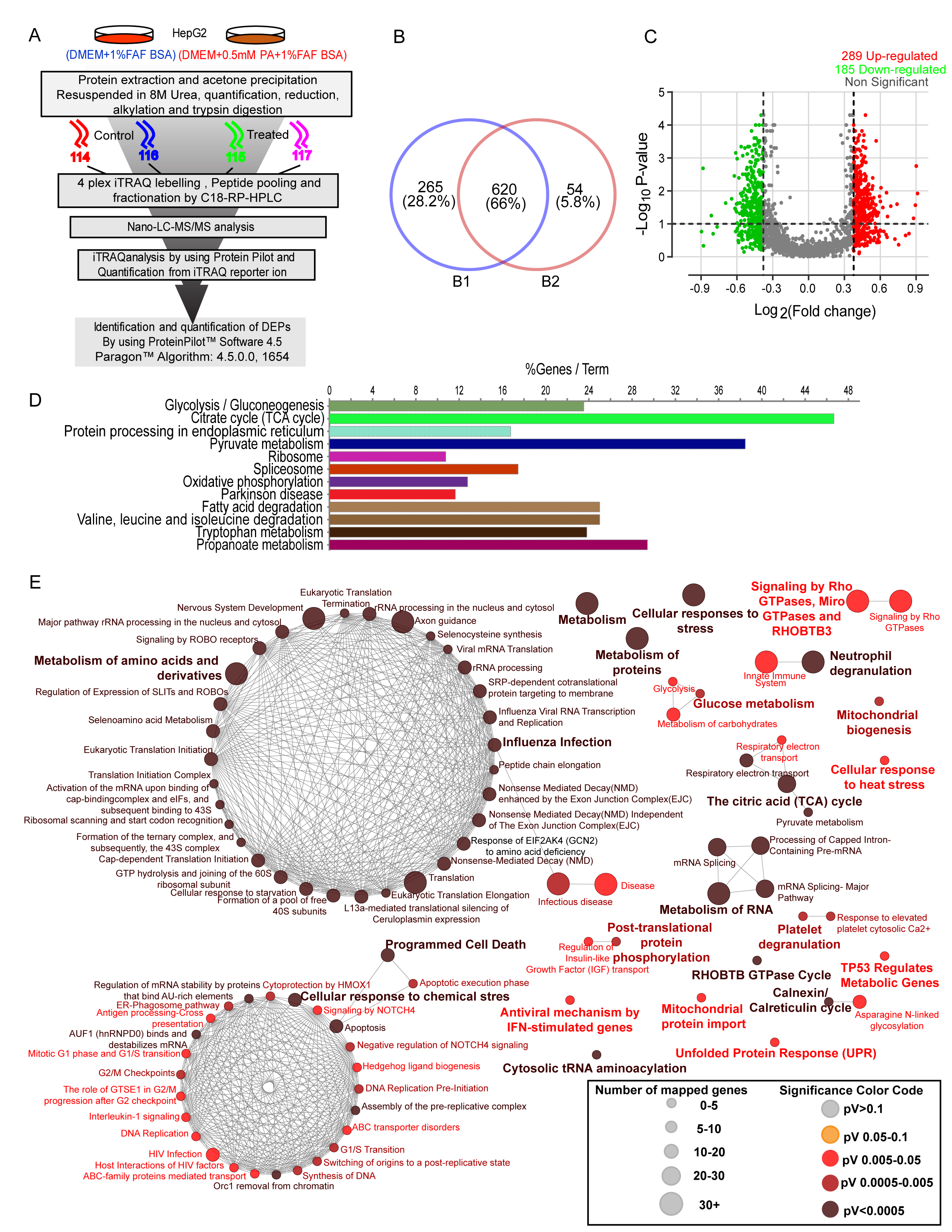
**

**Figure:2**

**
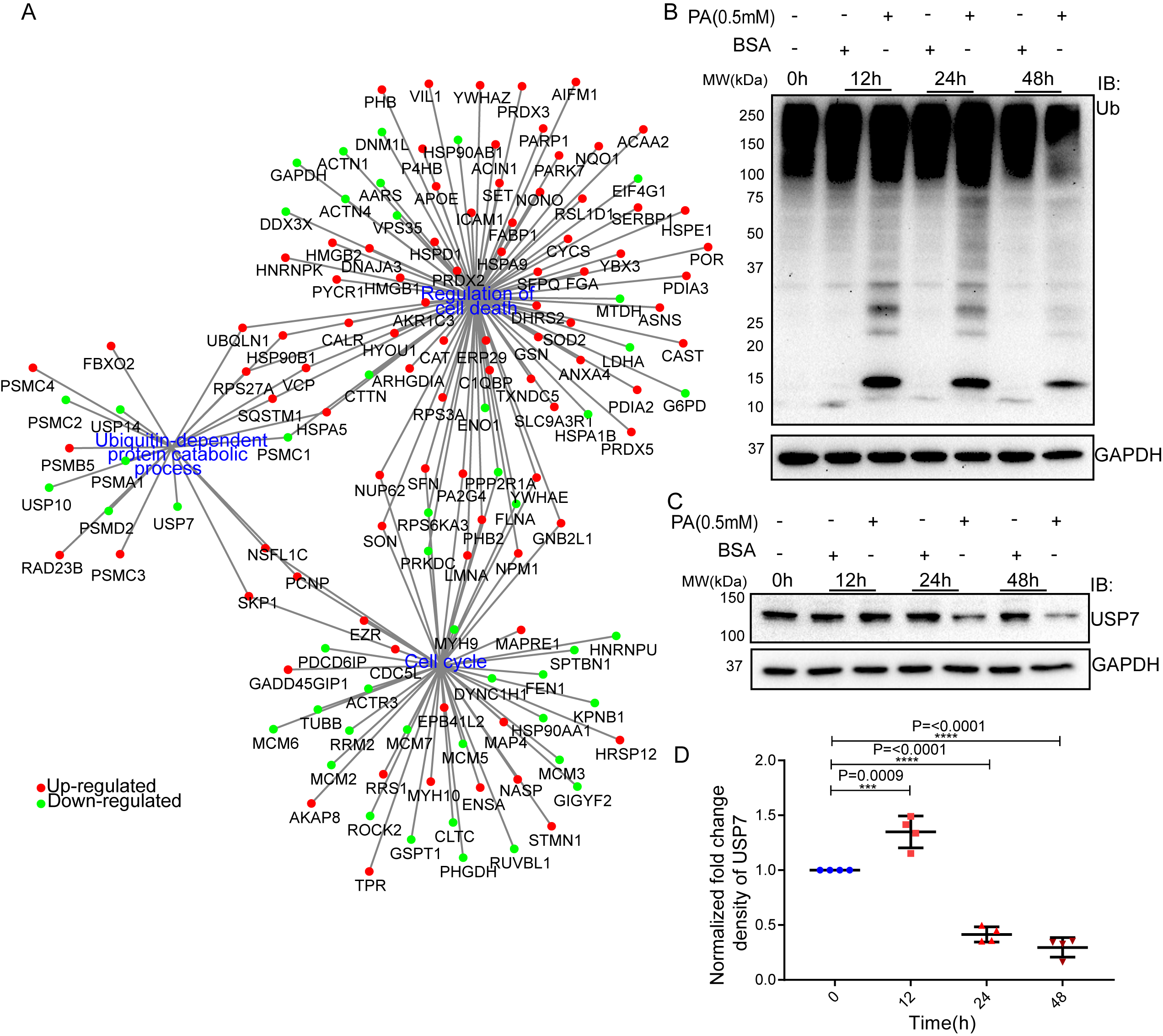
**

**Figure:3**

**
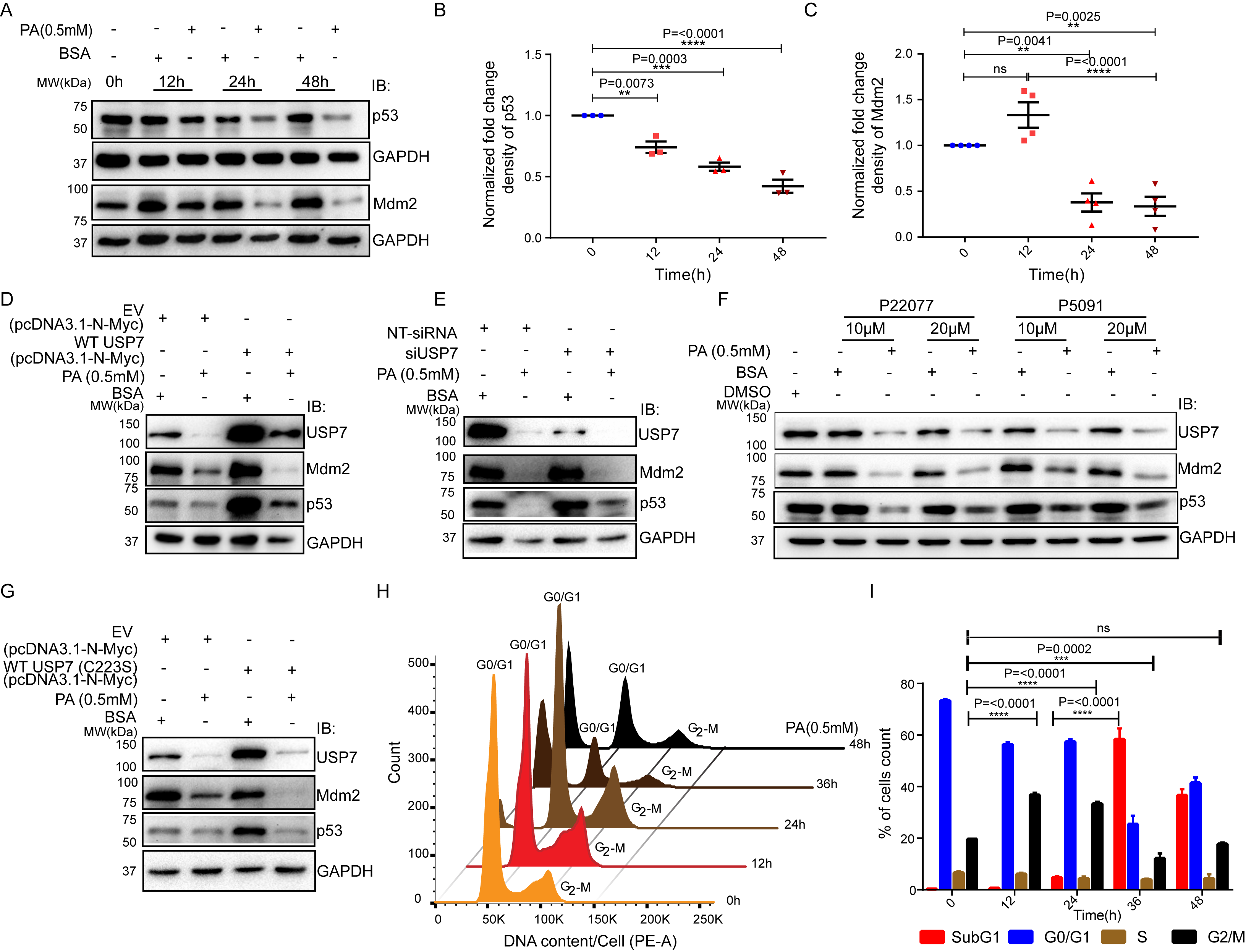
**

**Figure:4**

**
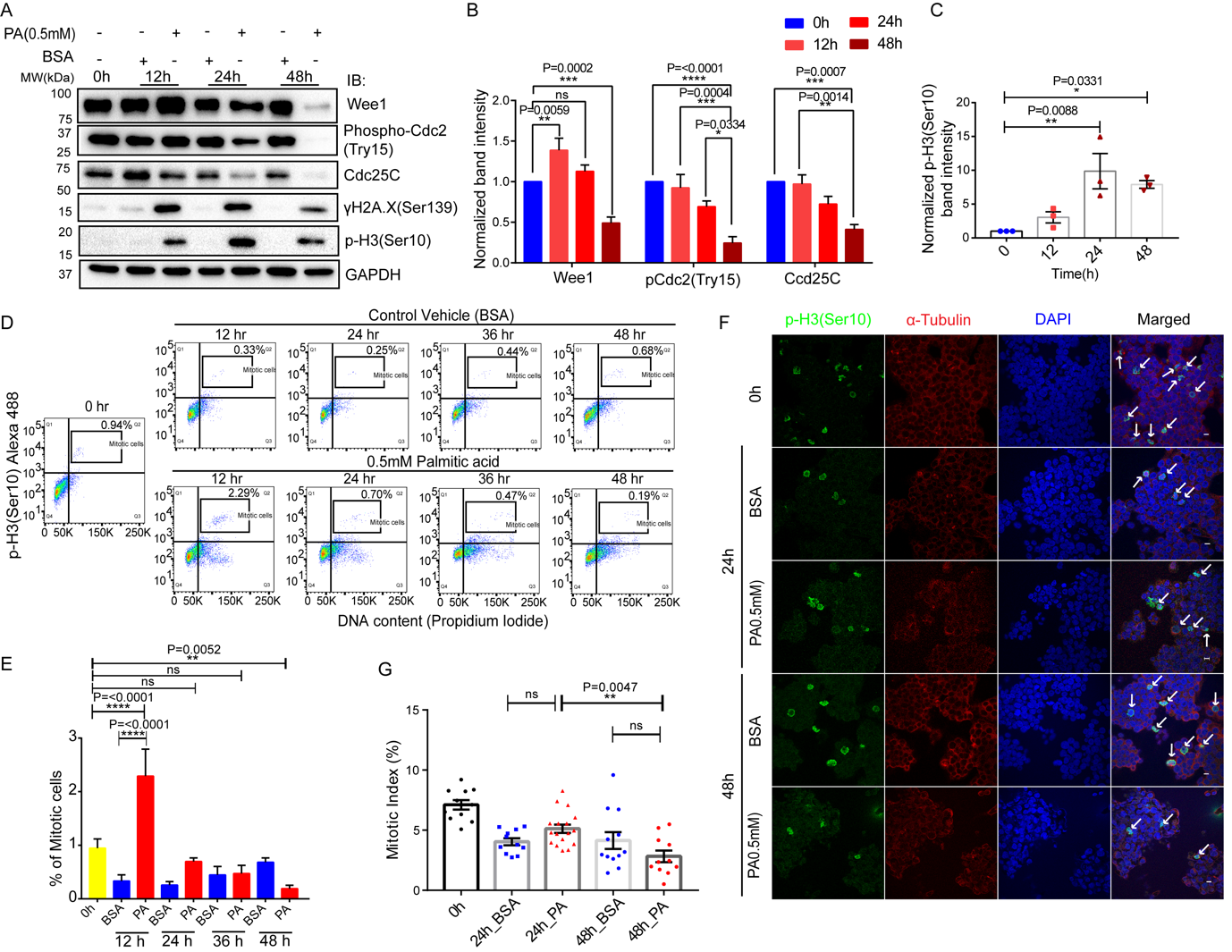
**

**Figure:5**

**
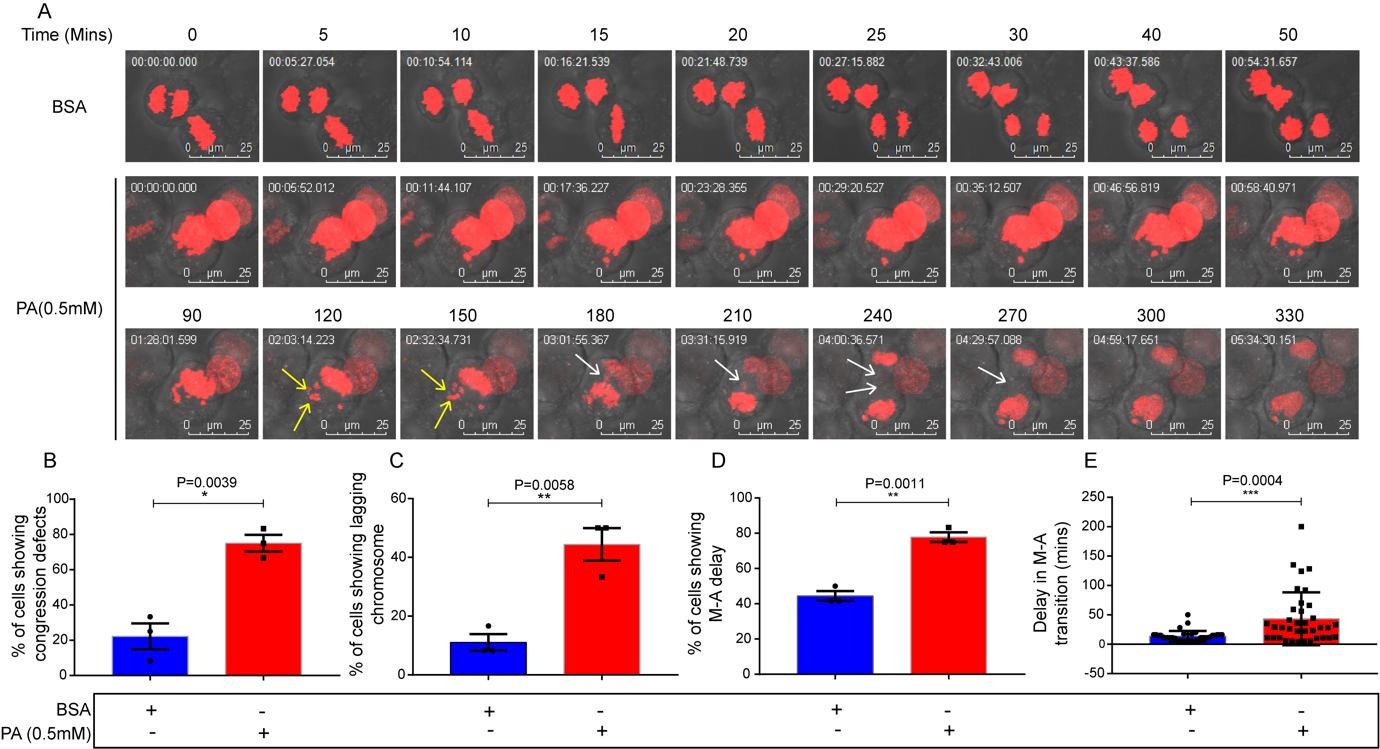
**

**Figure:6**

**
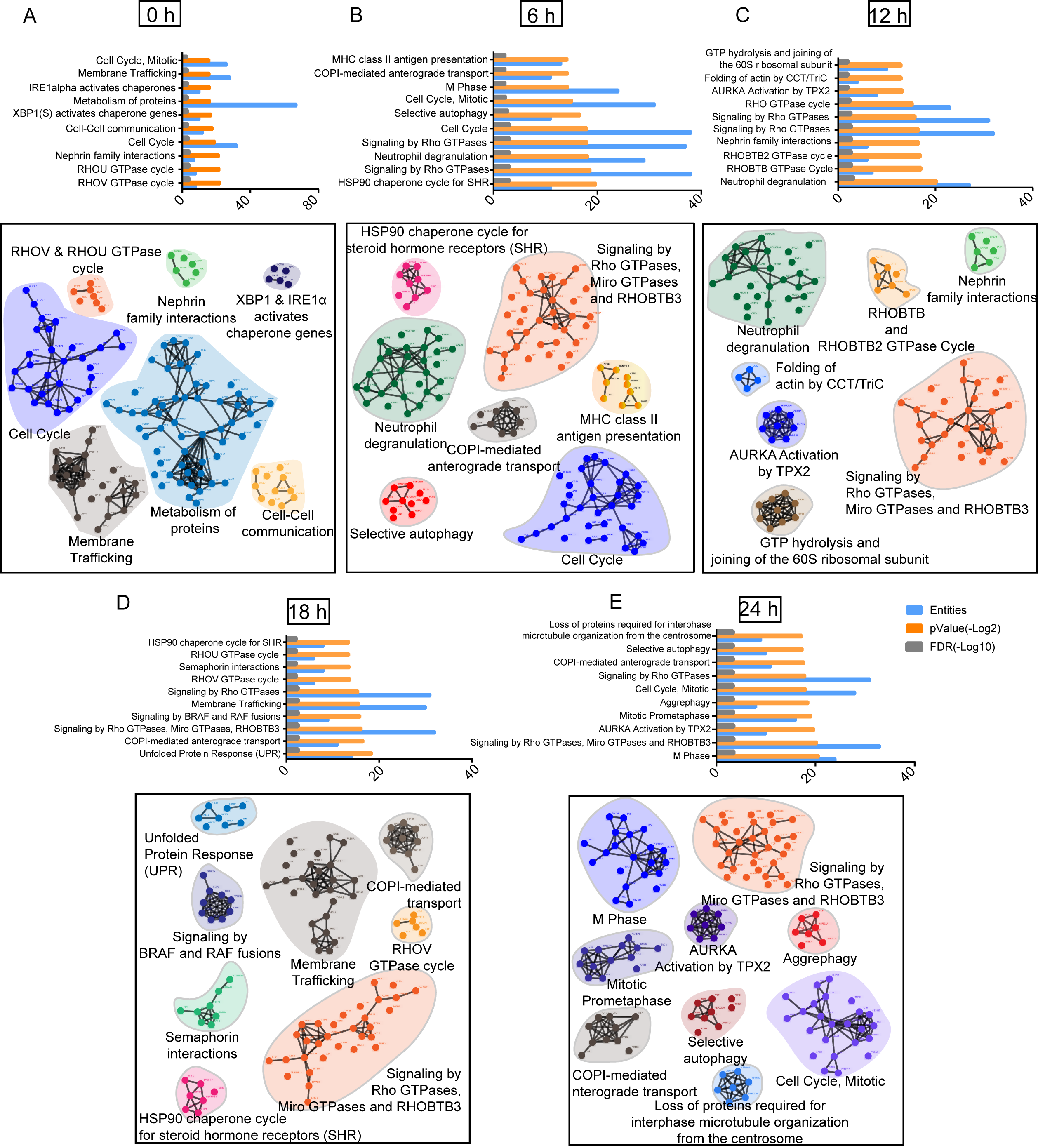
**

**Figure:7**

**
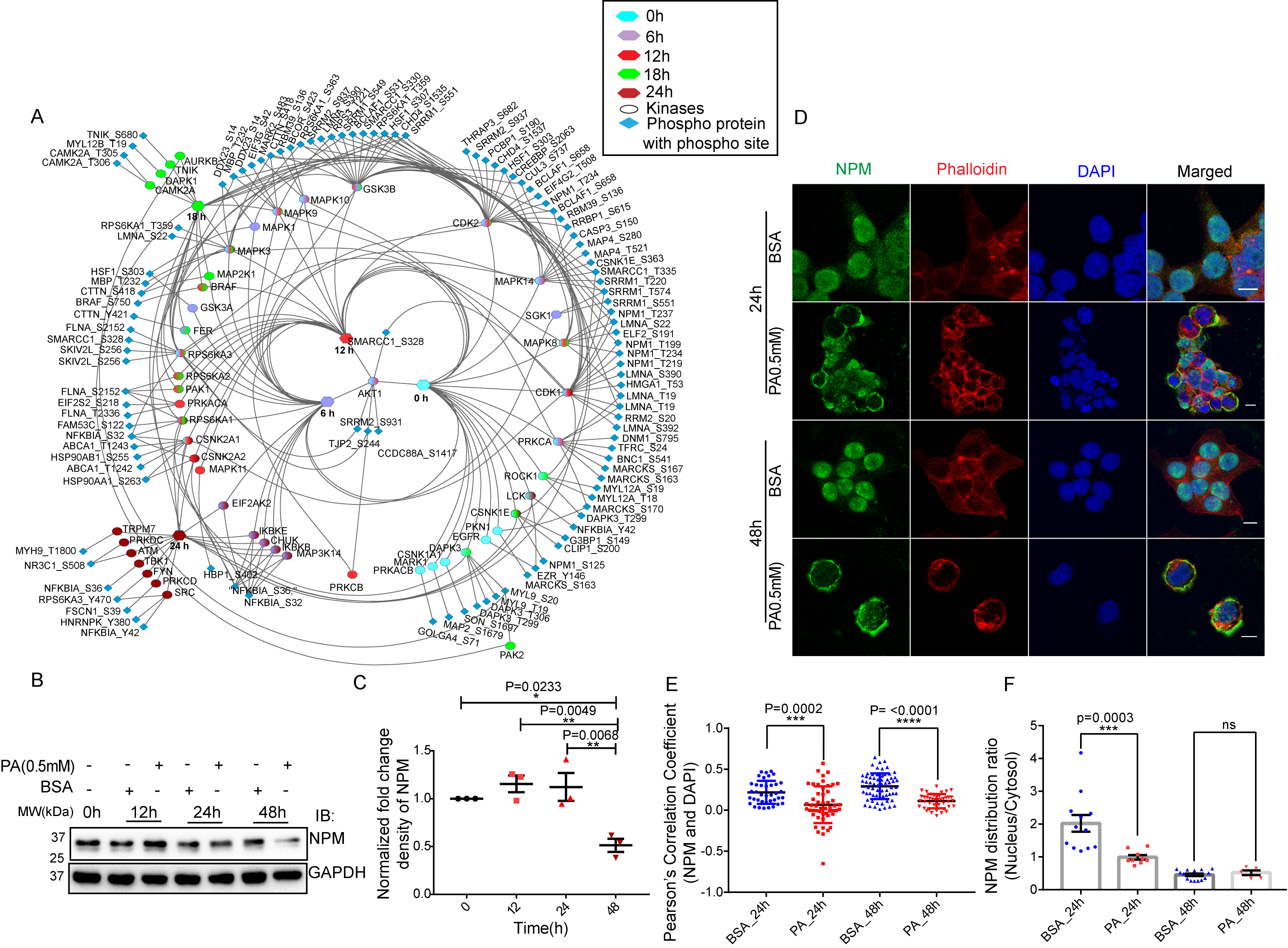
**

**Figure:8**

**
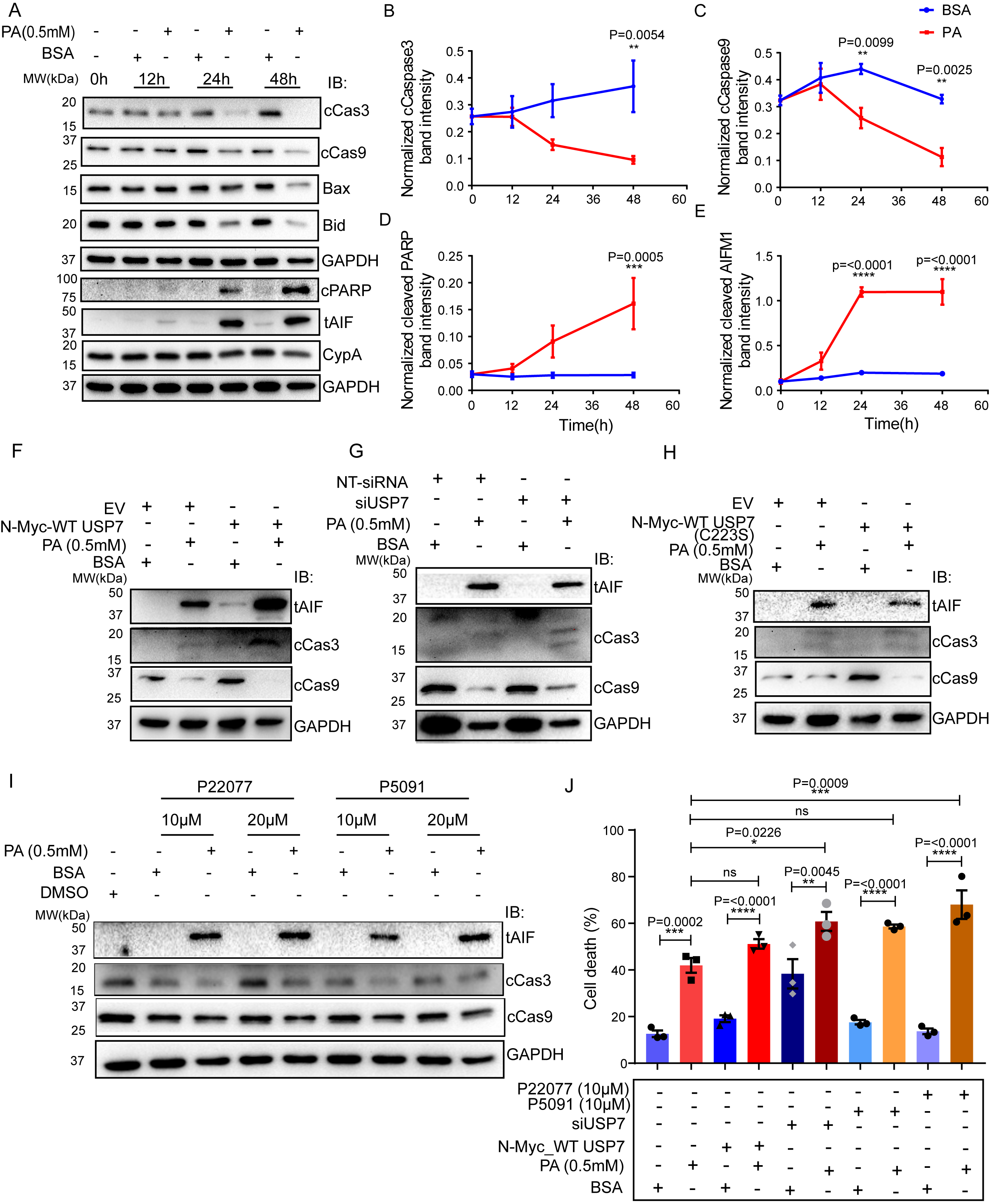
**

**Figure:9**

**
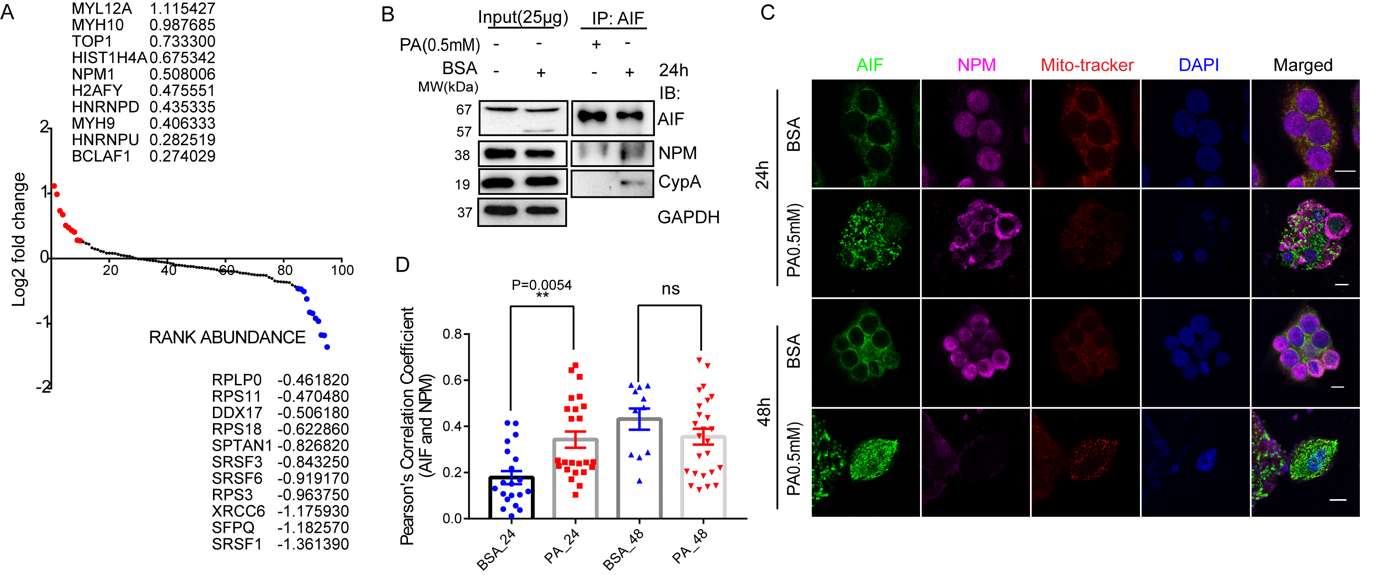
**
